# Hyocholic Acid Species as the Key Modulator for Cecal Epithelial Homeostasis in Low-Birth-Weight Piglets

**DOI:** 10.3390/nu17213415

**Published:** 2025-10-30

**Authors:** Chang Yin, Xuan Liu, Wei Fang, Qingshi Meng, Xiaohui Feng, Weidong Zhang, Guoqi Dang, Ruqing Zhong, Liang Chen, Zirong Wang, Hongfu Zhang

**Affiliations:** 1 College of Animal Science, Xinjiang Agricultural University, Urumqi 830052, China; 2 State Key Laboratory of Animal Nutrition and Feeding, Institute of Animal Science, Chinese Academy of Agricultural Sciences, Beijing 100193, China; 3 Academy of National Food and Strategic Reserves Administration, Beijing 100037, China

**Keywords:** low birth weight, bile acid, hyocholic acid species, gut barrier function

## Abstract

**Background**: Low birth weight (LBW) is correlated with gut microbiota dysbiosis and intestinal barrier function disruption, increasing susceptibility to enteric diseases. These alterations underscore the critical need to identify key regulators of gut homeostasis, among which bile acids are increasingly recognized as pivotal for barrier integrity, microbial ecology, and host metabolism. **Methods**: Eight pairs of LBW (the initial BW was 0.850 ± 0.053 kg) and normal-birth-weight (NBW; 1.488 ± 0.083 kg) piglets were compared to evaluate cecal morphology and bile acid profiles. Subsequently, sixteen LBW piglets and eight NBW piglets were allocated into three groups: NBW (1.563 ± 0.052 kg), LBW control (LBW-CON; 0.950 ± 0.120 kg), and LBW with bile acid supplementation (LBW-bile powder; 0.925 ± 0.116 kg). Piglets in the LBW-bile powder group received 25 mg/kg BW of bile powder (hyodeoxycholic acid-enriched) by daily oral gavage for 14 days. **Results**: LBW piglets exhibited retarded cecal development and lower abundance of hyocholic acid species (*p* = 0.006). Importantly, bile powder supplementation significantly improved cecal length (*p* = 0.009) and mucosal thickness (*p* = 0.020) compared with LBW-CON piglets. Microbial analysis showed that the microbial dysbiosis index was restored to near-normal levels. Transcriptomic analysis revealed impaired extracellular matrix structure and mucus secretion in LBW piglets. Notably, bile powder supplementation markedly upregulated the protein expression of WNT8B (*p* < 0.001) and the bile acid receptors (i.e., GPBAR1 and FXR), alongside enhanced tight junctions and the goblet cell marker mucin-2 expression (*p* < 0.05). **Conclusions**: These findings suggest that specific bile acid supplementation improves gut barrier function and partially supports cecal development in LBW piglets.

## 1. Introduction

Intrauterine growth restriction (IUGR), characterized by an abnormal fetal growth pattern, affects approximately 8–10% of human pregnancies and is a major cause of low birth weight (LBW) [1]. The condition also occurs naturally in other mammals, including pigs, where it is associated with poor fetal growth, impaired development, and increased neonatal mortality [2]. In pig production, IUGR represents a significant challenge for animal health and productivity, leading to economic losses in the livestock industry. Notably, the growth retardation pattern in naturally occurring IUGR piglets closely resembles that of human neonates with IUGR [3]. Given their physiological similarities to humans—particularly in gastrointestinal structure and function—piglets also serve as a valuable animal model, providing insights that may inform strategies to improve early-life gut health and nutritional programming in newborns.

According to several studies, IUGR reduces the weight and length of the gut at birth and damages its morphological structure, function, and microbiota colonization [4]. Excessive apoptosis of intestinal epithelial cells has also been documented to compromise intestinal integrity, facilitating the translocation of luminal antigens into the lamina propria, which subsequently triggers an inflammatory response and the release of pro-inflammatory cytokines [5]. On the other hand, early colonization of microbes in the neonatal gut is crucial for regulating intestinal health. Previous study reported that LBW piglets exhibited a significantly higher abundance of opportunistic pathogens, including *Streptococcus*, *Enterococcus*, and *Moraxella* in the intestine, thereby elevating the risk of bacterial infections [6]. These changes underscore the critical need to understand the key regulators of gut homeostasis. There has been an accumulating interest in bile acids (BAs), which are integral to digestive processes and serve as crucial mediators of intercellular communication within the gut-liver axis [7]. Such molecules function as a key signal molecule to activate cell signaling pathways to not only modulate glucose and lipid metabolism but also protect against the intestinal barrier dysfunction and endoplasmic reticulum [8,9,10,11]. Importantly, BA metabolism is tightly linked to gut microbes, which convert a portion of primary BAs escaping reabsorption in the distal ileum into secondary BAs, such as deoxycholic acid (DCA), lithocholic acid (LCA), and ursodeoxycholic acid (UDCA) [12]. Secondary BAs exert profound beneficial roles in the host due to their pleiotropic actions in metabolism and immune homeostasis [13]. In turn, BAs especially unconjugated types are potent antibacterial compounds and play an important role in inhibiting bile-sensitive germs to shape the gut microbial ecology, as well as affect the host metabolism indirectly [14].

However, LBW piglets are susceptible to abnormal BA metabolism, attributed to delayed hepatic development and deficiency in gut microbiota colonization [15,16]. It was reported that the plasma total BA concentrations were elevated in IUGR pigs at the 25 kg body weight stage and tended to increase at 28-day-old, alongside upregulated hepatic genes involved in BA synthesis (e.g., CYP7A1, CYP27A1) and transporters (e.g., NTCP), suggest potential dysregulation of BA homeostasis [17]. Our prior research demonstrated that gut dysbiosis-induced reductions in secondary BAs, such as LCA and hyodeoxycholic acid (HDCA), are significantly associated with gut barrier dysfunction in diarrhea piglets [18]. Notably, a recent study showed hyocholic acid species (e.g., HCA, HDCA) and UDCA are major intestinal BAs in piglets, and these key components were found to be decreased in the LBW piglets [19]. Additionally, the microbiota from LBW piglets was further transferred to antibiotic-treated mice, resulting in intestinal inflammation. Previous studies also identified elevated contents of pro-inflammatory cytokines, specifically interleukin-1β (IL-1β) and tumor necrosis factor-α (TNF-α) in the intestinal of LBW piglets [20,21]. In the present study, we initially observed notable alterations in gut morphology, accompanied by a strong reduction in the concentration of HCA species (HCAs) within the cecum of LBW piglets. The underlying mechanism between HCAs and gut barrier integrity remains inadequately understood. We therefore hypothesized that HCAs, particularly the secondary form HDCA, may play an essential role in gut health. We subsequently found that oral administration of HCAs modulated expression of key bile acid receptors, altered gut microbiota, and ultimately led to significant improvements in the development and function of the intestinal barrier. This study revealed the critical role of specific bile acids in maintaining gut homeostasis and proposed potential nutritional interventions for addressing LBW-induced disruptions in gut microflora and barrier function in suckling piglets.

## 2. Materials and Methods

### 2.1. Animals and Experimental Design

The piglets used in this study (Duroc × Landrace × Large White crossbreed) were naturally born from multiparous sows (Landrace × Large White, with 3–6 parities) following a gestation period of 113–114 days. The sows were provided with a basic diet that met or exceeded the nutritional requirements outlined in NRC (2012) guidelines, with free access to water. Within each litter, one normal-birth-weight (NBW, 1.35–1.55 kg) piglet and one low-birth-weight (LBW; the initial BW is less than 70% of the NBW range) piglet were tagged on the same day of birth to ensure uniformity in postnatal care and lactation access. The total number of piglets per litter was maintained at 10 through standardized farrowing management to minimize suckling competition between LBW and NBW piglets. Both male and female piglets were included in the study, with equal representation from each sex across all experimental groups. All piglets were housed within their original litter on a plastic-slatted floor during the entire trial period, and efforts were made to maintain a clean environment to mitigate the risk of disease occurrence. All sows and piglets in the experiment were not administered antibiotics or any other drugs.

We first selected eight pairs of neonatal piglets from each of the eight litters that meet the requirements, i.e., LBW group (the initial BW was 0.850 ± 0.053 kg) and NBW group (1.488 ± 0.083 kg). Piglets were euthanized via an overdose of Zoletil on day 7. The intestinal samples and contents were collected, in which the tissue samples were fixed in 4% paraformaldehyde for histological examinations, and the intestinal contents were flash-frozen in liquid nitrogen and stored at −80 °C for BAs detection. Next, a total of sixteen LBW piglets and eight NBW piglets were obtained from sixteen litters according to the above procedures. These piglets were divided into three groups, i.e., NBW group (1.563 ± 0.052 kg), LBW-CON group (0.950 ± 0.120 kg), and LBW-bile powder group (0.925 ± 0.116 kg). An adaptation period of 7 days followed by an experimental period of 14 days was maintained. BA (porcine bile extract, HCAs-enriched powder extracted from pig bile) was administered once daily via oral gavage at a dosage of 25 mg/kg BW (the dose is safe concerning in vivo animal treatment as supported by similar studies) [22,23], with a saline solution containing a small amount of DMSO serving as the control (NBW and LBW-CON). LC-MS/MS analysis revealed that the BA powder compound primarily contains hyodeoxycholic acid (HDCA), with a minor proportion of hyocholic acid (HCA), as detailed in Table S1. On day 21, piglets from each group were weighed and euthanized using the same method described above. The intestinal samples and contents were collected, and the tissue samples were divided into two portions. One portion was flash-frozen in liquid nitrogen and stored at −80 °C for subsequent molecular biological analyses, while the other portion was fixed in 4% paraformaldehyde for histological examinations.

### 2.2. Determination of Total Bile Acid Levels

Total bile acid (TBA) concentrations in serum and liver samples were measured using commercially available assay kit (Jiancheng Bioengineering Institute, Nanjing, China), following the manufacturer’s protocol. For liver analysis, approximately 100 mg of tissue was homogenized in nine volumes (1:9, *w*/*v*) of ice-cold 0.9% saline solution using a tissue grinder. The homogenates were centrifuged at 3000× *g* for 10 min at 4 °C to obtain the clarified supernatants, which were subsequently used for TBA quantification.

### 2.3. Histological Analysis

After 24 h of fixation, intestinal tissues were dehydrated with gradient alcohol, made transparent with xylene, and embedded in paraffin. Hematoxylin and eosin (H&E) staining was performed to assess intestinal morphology (H&E staining kit, Solarbio, Beijing, China). Cross-sections with a thickness of 5 μm were prepared from each tissue sample, stained, and examined using a Leica DM2000 light microscope (Leica Microsystems, Wetzlar, Germany). For each tissue sample, four equidistant points along the same cross-section were selected for quantitative analysis. At each point, the thicknesses of the mucosa, submucosa, and muscularis propria were independently measured using ImageJ software (v1.8.0). The mucosa was defined as the region extending from the luminal surface to the base of the crypts; the submucosa was defined as the connective tissue layer between the base of the mucosa and the beginning of the muscularis propria; and the muscularis propria was measured from the inner circular muscle layer to the outer longitudinal muscle layer. All measurements were performed under consistent magnification and based on standardized criteria to minimize observer bias.

### 2.4. Real-Time Quantitative PCR

The essential genes involved in the synthesis mechanisms of BA were analyzed. Primers (see Table S2) underwent characterization through amplification efficiency assessments and agarose gel electrophoresis. RNA was extracted from liver tissue utilizing a total RNA extraction kit (Cat#R013-50, Gene Better, Beijing, China). RNA integrity and concentration were evaluated using 1.2% agarose gel electrophoresis and a microspectrophotometer (Nanodrop Technologies Inc., Wilmington, DE, USA). First-strand cDNA was made using the High-Capacity cDNA Archive kit (Takara, Dalian, China) following the manufacturer’s protocol. Approximately 2 μg of RNA, with an average A260/A280 ratio of 1.9, was used for transcription. Real-time PCR was conducted on 384-well microplates, and the data were analyzed using the ABI 7900-HT platform (Applied Biosciences, Carlsbad, CA, USA). Gene expression values were determined using the comparative CT method (2^−ΔΔCT^), with GAPDH serving as reference genes.

### 2.5. 16S rRNA Gene Sequencing

DNA extraction, PCR amplification, DNA quantification, and Illumina Nextseq2000 sequencing were performed following the standardized protocol of Shanghai Majorbio Bio-pharm Technology (Shanghai, China). Total bacterial DNA was extracted from cecal contents using the Qiagen DNA Isolation Kit (Qiagen, Hilden, Germany) according to the manufacturer’s instructions. The V3-V4 hypervariable region of the 16S rRNA gene was amplified with primers 338F (5′-ACTCCTACGGGAGGCAGCAG-3′) and 806R (5′-GGACTACHVGGGTWTCTAAT-3′). Amplicons were sequenced using the Illumina Nextseq2000 platform. Raw sequencing data were analyzed on the Majorbio Cloud Platform (www.majorbio.com, accessed on 15 September 2025) to assess gut microbial diversity and composition. Raw sequencing data were processed and analyzed using the Majorbio Cloud Platform (www.majorbio.com). The analysis workflow included sequence quality control using fastp (v0.19.6) and merging of paired-end reads with FLASH (v1.2.7). Then the optimized sequences were clustered into operational taxonomic units (OTUs) using UPARSE 7.1 at a 97% sequence similarity level. The taxonomy of each OTU representative sequence was analyzed using RDP Classifier version 2.2 against the 16S rRNA gene database (e.g., Silva v138) with a confidence threshold of 70%. More detailed information on the sequencing and bioinformatics procedures can be found in references [24,25].

### 2.6. RNA-Seq and Data Analysis

Total RNA from intestinal mucosa samples was isolated using TRIzol^®^ Reagent (Invitrogen, Carlsbad, CA, USA) according to the manufacturer’s instructions. Library construction and sequencing were performed by Majorbio. RNA-seq transcriptome library (sus scrofa) was prepared following Illumina^®^ Stranded mRNA Prep, Ligation (San Diego, CA, USA). Purified libraries were quantified by Qubit 4.0 Fluorometer (Life Technologies, Carlsbad, CA, USA), the sequencing library was then performed on Illumina NovaSeq X Plus platform (PE150) using NovaSeq Reagent Kit (Illumina, San Diego, CA, USA). Raw paired-end reads were trimmed and quality controlled by fastp (v0.20.0) with default parameters. Clean reads were aligned to the Sus scrofa reference genome using HISAT2 (v2.2.1), and uniquely mapped fragments for each gene were counted with StringTie (v1.3.0). The raw read count matrix generated by StringTie was used as the input for DESeq2 to identify differentially expressed genes (DEGs). The TPM and RSEM-normalized expression values were used only for visualization and for selecting highly expressed genes for visualization and descriptive analysis only. Gene abundance was quantified using RSEM (v1.3.3) [26]. KEGG pathway enrichment analysis of DEGs was performed using the scipy package in Python (v3.7).

### 2.7. Quantitative Analysis of Cecal Metabolites

Short-chain fatty acids (SCFAs) were quantified using gas chromatography–mass spectrometry (GC-MS) analysis as described in our previous study [27]. Briefly, the cecal contents were dissolved in distilled water and extracted. The samples were centrifuged at 9000× *g*, and the supernatant was mixed with 25% (*w*/*v*) metaphosphoric acid at a 1:9 ratio. After a second centrifugation at 10,000× *g*, the supernatant was analyzed using an Agilent 6890N GC (Palo Alto, CA, USA).

BAs in cecal contents were profiled as previously described [28]. The samples were desalted utilizing a Bond Elut C18 column (500 mg/6 mL, Agilent Technologies, Santa Clara, CA, USA), which had been pre-activated with 5 mL of methanol, and subsequently eluted in methanol. All analyses were conducted using a Waters ACQUITY UPLC system coupled with a Waters Xevo TQ-S Mass Spectrometer equipped with an ESI source, controlled by MassLynx 4.1 software (Waters, Milford, MA, USA). Chromatographic separations were achieved using a Waters ACQUITY BEH C18 column (1.7 μm, 100 mm × 2.1 mm internal dimensions). The raw data, acquired in negative mode, were processed using Waters TargetLynx Application Manager (v4.1) to derive calibration equations and determine the concentrations of various BA in each sample. Moreover, the calibration curve and the corresponding regression coefficients were established through internal standard adjustment with CA-d4, CDCA-d4, and LCA-d4.

### 2.8. Western Blot (WB) Analysis

Expression of BA receptors and intestinal barrier function and other related proteins in cecal mucosa were determined by WB analysis which was conducted as previously described [29,30]. BA-related proteins were detected by primary antibodies: anti-GPBAR1 (Cat#A25850; ABclonal, Wuhan, China; 1:800 dilution) and anti-NR1H4 (FXR; Cat#bs-12867R; Bioss, Beijing, China; 1:1000 dilution). Tight junction (TJ) proteins were detected by primary antibodies: anti-Occludin (Cat#DF7504; Affinity, Jiangsu, China; 1:2000 dilution), anti-ZO-1 (Cat#21773-1-AP; Proteintech, Wuhan, China; 1:5000 dilution), anti-Claudin-1 (Cat#DF6919, Affinity, 1:1000 dilution), Claudin-4 (Cat#DF5350, Affinity, 1:1000 dilution). And other related proteins including anti-WNT8B (Cat#DF2441, Affinity, 1:1000 dilution), anti-IL-1β (Cat#bs-0812R, Bioss, 1:1000 dilution), anti-STAT-4 (Cat#AF6441, Affinity, 1:1000 dilution), and β-Actin (Cat#D110001; Sangon Biotech, Shanghai, China; 1:2000 dilution). Subsequently, incubated with the secondary antibodies (Cat#D110058, Sangon Biotech, 1:5000 dilution). Finally, the Western ECL-plus reagent was used for chemiluminescence detection, and band densities were quantified using the GelDocTM XR molecular imager and Image Lab software v1.51 (Bio-Rad, Hercules, CA, USA).

### 2.9. Immunofluorescence Staining of Cecal Tissue Sections

Tissue sections were deparaffinized in xylene and rehydrated through a graded ethanol series (100% to 70%, 3 min per bath). Epitope retrieval was performed by incubating the slides in 1 mM EDTA buffer (pH 8.0), brought to a boil, and then kept at sub-boiling temperature for 30–40 min. The slides were blocked with normal goat serum and incubated overnight at 4 °C with an anti-MUC2 antibody (Cat#AB4767, ABclonal, 1:150 dilution). After washes using PBS-T (PBS with 0.1% Triton X-100), the slides were incubated for 60 min at room temperature with a secondary antibody (Cat#A0516; Beyotime, Shanghai, China; 1:500 dilution) and 1 µg/mL DAPI in PBS for 5 min. After that, the slides were washed three times with PBS-T and sealed with coverslips. Stained tissues were analyzed with an inverted Leica DM2000 microscope (Wetzlar, Germany) equipped with epifluorescence filters and photographed using a 20× objective. Finally, the integrated fluorescence intensity of MUC2 was measured using ImageJ software. Background-corrected values were normalized to the mean fluorescence intensity of the NBW group (set as 1).

### 2.10. Statistical Analysis

Statistical analyses were performed using JMP 10.0 (SAS Institute, Cary, NC, USA) and GraphPad Prism v8.0 (GraphPad Software Inc., San Diego, CA, USA). Data are expressed as mean ± standard deviation (SD in tables) or mean ± standard error (SE in figures). The Shapiro–Wilk test and Levene’s test were used to assess data normality and homogeneity of variance, respectively. All datasets satisfied the assumptions of normality and equal variance.

For comparisons between two groups (in Exp.1), including alterations in bile acid profiles, statistical significance was assessed using an unpaired two-tailed Student’s *t*-test. For three group comparisons (in Exp.2), including BW, cecal morphological parameters, SCFA contents and proteins expression, one-way analysis of variance (ANOVA) followed by least squares means pairwise comparisons with multiple testing adjustment was applied to assess group differences. A *p*-value of less than 0.05 was considered statistically significant, with trends noted for 0.05 < *p* ≤ 0.10. Significance levels are indicated as follows: * *p* < 0.05, ** *p* < 0.01, *** *p* < 0.001.

Subsequently, other multivariate analyses were used. Alpha diversity and the relative abundances of cecal mucosal bacterial phyla and genera were assessed using the non-parametric Wilcoxon rank-sum test. Beta diversity of microbial communities was evaluated using Principal Coordinates Analysis (PCoA), and differences between groups were tested using Analysis of Similarity (ANOSIM). For differential microbial analysis across multiple groups, the Kruskal–Wallis rank-sum test was applied, with multiple testing corrections performed using the false discovery rate (FDR) method, and significant differences were indicated with asterisks (*) on the bar plots at both the phylum and genus levels. The linear discriminant analysis (LDA) was employed to estimate the impact of differentially abundant species on group distinctions. The microbial dysbiosis index (MDI) was calculated as follows:

$$MDI=log10[\frac{Total\;abundance\;in\;genera\;increased\;in\;LBW\;group}{Total\;abundance\;in\;genera\;decreased\;in\;LBW\;group}]$$

Transcriptomic differential expression analysis was performed using DESeq2, based on the raw count data. The resulting *p*-values were adjusted for multiple testing using the Benjamini–Hochberg false discovery rate (FDR) correction. However, as the number of DEGs passing the stringent FDR threshold of <0.05 was limited, genes with *p* < 0.05 and a 1.2-fold change were also considered as putative DEGs for exploratory pathway analysis. Finally, KEGG pathway enrichment analysis of DEGs was conducted using the scipy.stats module in Python, with FDR correction applied to enrichment *p*-values. Detailed statistical parameters are provided in the figure legends.

## 3. Results

### 3.1. HCAs Deficiency Was Found in the Cecum of LBW Piglets

**Figure 1:**
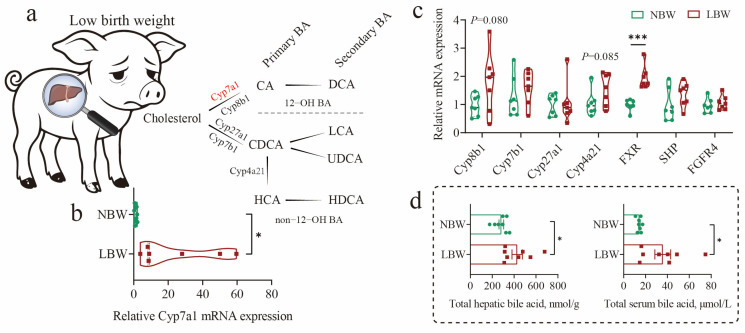

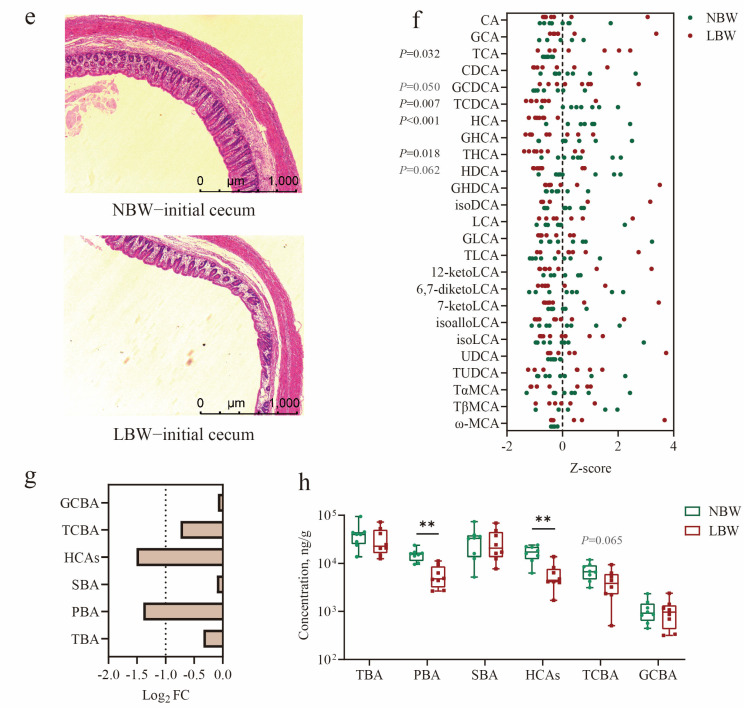
Dysregulation of bile acid metabolism and altered cecal morphology in low-birth-weight piglets. (**a**–**d**) Diagram of the bile acid (BA) synthetic pathway in pigs (**a**), and relative mRNA expression levels of key hepatic enzymes involved in BA synthesis (**b**,**c**), along with total BA concentrations in liver and serum (**d**). (**e**) Representative images of cecal morphology at day 7 evaluated by H&E staining (scale bars represent 1000 μm). (**f**) BA composition in cecal contents. And Log_2_ fold change (**g**) and concentrations (**h**) of individual BA species in the cecum. Data are presented as mean ± SE or median (minimum to maximum), *n* = 8 per group. * *p* < 0.05, ** *p* < 0.01, *** *p* < 0.001.

In this study, we first observed that early low birth weight was accompanied by dysregulation of BA metabolism. This was characterized by a significant upregulation of the classical hepatic BA synthesis pathway, as indicated by elevated mRNA expression of Cyp7a1 (*p* < 0.05; Figure 1a,b), a key rate-limiting enzyme. And its negative feedback regulator, such as FXR, was also upregulated (*p* < 0.0001; Figure 1c). Consequently, total BA levels were markedly increased in both the liver and serum of LBW piglets (*p* < 0.05; Figure 1d). Additionally, LBW piglets significantly reduced gut weight and length at birth and showed delayed intestinal development and compromised function, as previously reported [4]. We also found that LBW piglets exhibited developmental retardation in cecal morphology (Figure 1e). Further analysis revealed obvious alterations in BA profiles within the cecum **(**Figure 1f; Table S3). There was a notable reduction in the levels of HCAs (5884.742 versus 16,759.006 ng/g, 0.351-fold) and primary BAs (PBA, 5800.731 versus 15,201.451 ng/g, 0.382-fold) in the cecum of LBW piglets (Figure 1b,c; Table S4. Among them, the contents of HCA (*p* = 0.0002) and THCA (*p* = 0.018) were significantly decreased, and HDCA showed a decreased trend (*p* = 0.062).

### 3.2. Oral Gavage of HCAs Restores the Cecal Length and Mucosal Thickness of LBW Piglets to Healthy Levels

**Figure 2:**
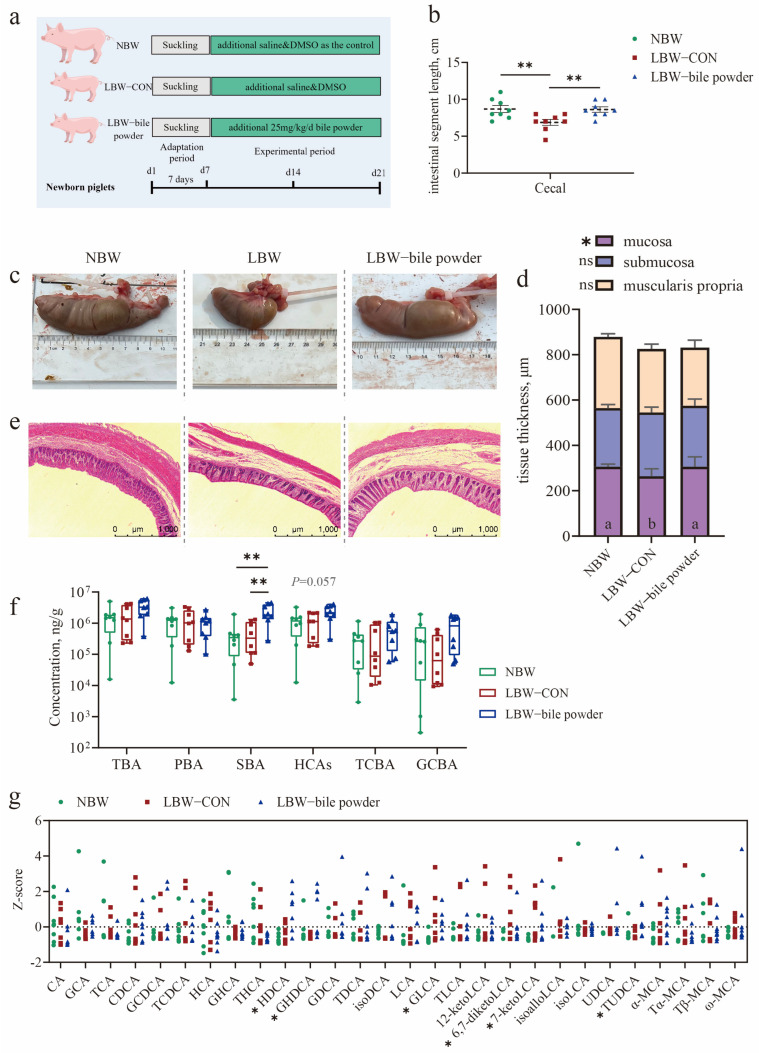
Hyocholic acid species (HCAs) restore cecal length and mucosal thickness in low-birth-weight piglets. (**a**) Schematic timeline of the experimental procedure. (**b**,**d**) Statistical analysis of cecal length (**b**) and mucosal thickness (**d**) among the three groups. (**c**) Representative images of cecum tissue. (**e**) H&E staining of cecal sections (scale bars represent 1000 μm). (**f**) Concentrations of individual bile acid (BA) species in cecal contents. (**g**) BA composition in the cecum. Data are presented as mean ± SE or median (minimum to maximum), *n* = 8 per group. * *p* < 0.05, ** *p* < 0.01; Bars with different lowercase letters are significantly different (*p* < 0.05).

To identify BAs especially HCAs are the key regulators of gut homeostasis in pig neonates. Following a 7-day acclimatization period (Figure 2a), a cohort of sixteen LBW piglets and eight NBW piglets were used in the study. The mean birth weight of LBW piglets was also significantly lower than that of NBW piglets (*p* < 0.0001; Table 1). And then, HCAs were administered by oral gavage to LBW piglets on the 7-day-old. Our results showed that although HCAs supplementation did not significantly affect the growth of suckling LBW piglets, it notably restored cecum length to near-normal levels after 14 days of trial (*p* < 0.01; Figure 2b,c; Table S5). In addition, cecal mucosal thickness was significantly increased in the LBW-bile powder group compared to the LBW-CON group (*p* < 0.05; Figure 2d,e; Table S5).

To confirm whether the supplemented BAs effectively reached the lower gut, we also performed quantitative analysis using LC-MS. The results showed a significant increase in SBAs in the cecum of LBW piglets receiving BA supplementation (*p* < 0.05; Figure 2f; Table S6), along with a trend towards elevated HCAs (*p* = 0.057). BA profiling further revealed notable changes in several specific BAs, including HDCA, GHDCA, GLCA, TUDCA, 6,7-diketoLCA, and 7-ketoLCA (*p* < 0.05; Figure 2g; Table S7). Thus, our initial findings imply that HCAs are essential for early gut development and health.

### 3.3. Supplementation of HCAs Induces Alterations in Gut Microbial Ecology and Composition

**Figure 3:**
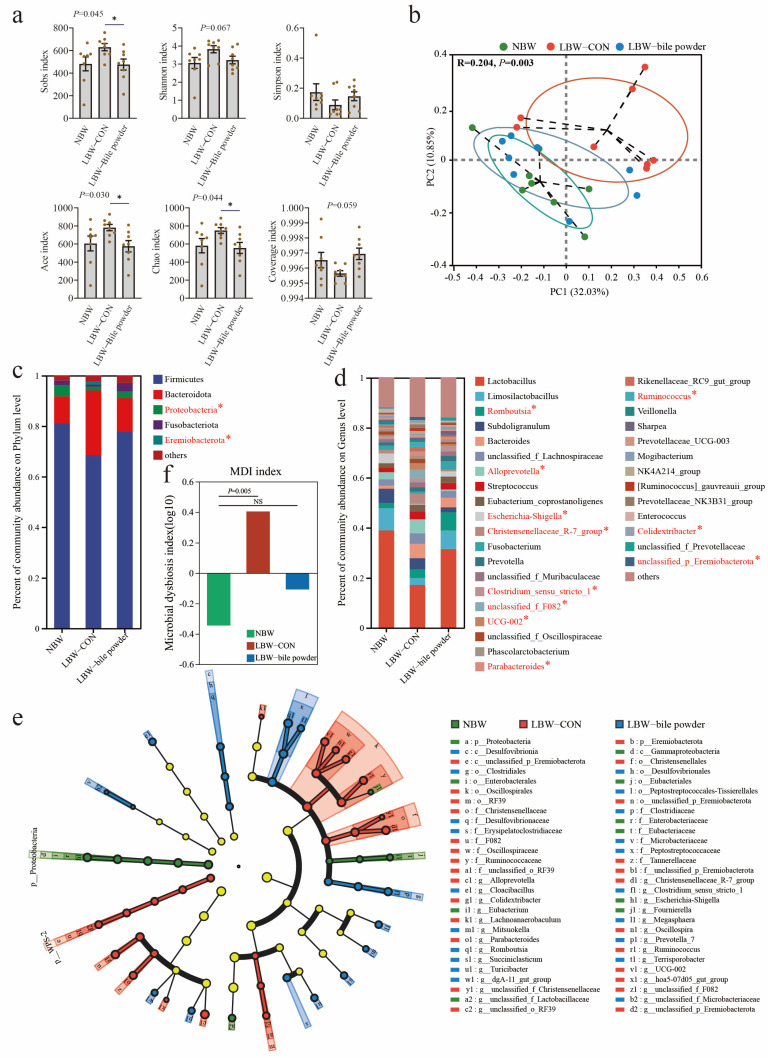
Hyocholic acid species (HCAs) alter cecal microbial composition. (**a**) Alpha diversity is represented by Sobs, Shannon, Simpson, ACE, Chao, and coverage indexes. (**b**) Beta diversity using PCoA plots based on Bray–Curtis dissimilarity matrices. The average phylum (**c**) and genus (**d**) distribution of gut microbiota, * indicates *p* < 0.05. (**e**) Discriminating taxa between the gut microbiota were analyzed using the linear discriminant analysis (LDA) effect size (LEfSe) method (Kruskal–Wallis test with *p* < 0.05 and LDA scores > 3.0). And (**f**) microbial dysbiosis index (MDI) on the Genus levels are presented (*n* = 8).

Prior research has demonstrated a marked disparity in the intestinal microbiome between LBW and NBW piglets, especially the dominant genus *Lactobacillus*, which was notably deficient in the LBW piglets [31]. We determined that LBW significantly impacted the cecal mucosal microbiota based on the statistical analysis of alpha and beta diversity (Figure 3a,b). In particular, beta diversity measures the between-group differences in diversity. Based on operational taxonomic unit (OTU) variance using Bray–Curtis distance matrices, there was a notable separation between LBW and NBW groups with the R^2^ value of 0.204 and *p* = 0.003 (ANOSIM). Unexpectedly, LBW piglets supplemented with HCAs appeared to modulate the diversity of cecal microbiota, rendering it comparable to that observed in healthy individuals.

To investigate the specific changes in gut microbiota among these groups, the bar chart for phylum and genus levels, as well as the linear discriminant effect size (LEfSe) cladograms, are displayed in Figure 3c–e. Relative abundance of Proteobacteria decreased while the Eremiobacterota increased at the phylum level was observed in the LBW-CON group. Moreover, there were several significantly different genera found among NBW, LBW-CON, and LBW-bile powder groups (Figure 3d shows only genera with over 1% relative abundance) including *Romboutsia* (BA-enriched), *Alloprevotella* (LBW-enriched), *Escherichia-Shigella* (NBW-enriched), *Christensenellaceae R-7* group (LBW-enriched), *Clostridium sensu stricto 1* (BA-enriched), unclassified *F082* (LBW-enriched), *UCG-002* (LBW-enriched), *Parabacteroides* (LBW-enriched), *Ruminococcus* (LBW-enriched), *Colidextribacter* (LBW-enriched), unclassified *Eremiobacterota* (LBW-enriched). Similarly, our findings also indicated a reduction in the abundance of the *Lactobacillus* genus in LBW piglets. This reduction was partially ameliorated through the supplementation of HCAs. However, the statistical analysis revealed that the difference between the three groups was insignificant (*p* > 0.05), potentially due to the high inter-individual variability within the three groups (38.92 ± 16.15 versus 17.18 ± 20.13 versus 31.59 ± 19.07%). Notably, the microbial dysbiosis index (MDI) at the genus level was significantly elevated in the LBW-CON group, whereas it exhibited substantial recovery in the LBW-bile powder group (Figure 3f).

SCFAs are critical metabolites generated by the microbiota, and their production directly reflects variations in gut microbiome colonization and development. However, the GC-MS analysis revealed no significant changes in SCFA contents including acetic acid, propionic acid, butyric acid, isovaleric acid, and valeric acid among the three groups (Table 2). This may suggest that LBW piglets supplemented with HCAs positively affect cecal development and barrier function, independent of microbial fermentation capacity during early neonatal stages.

### 3.4. Transcriptome Profiling Reveals Changes in Cecal Barrier Function in LBW Piglets

**Figure 4:**
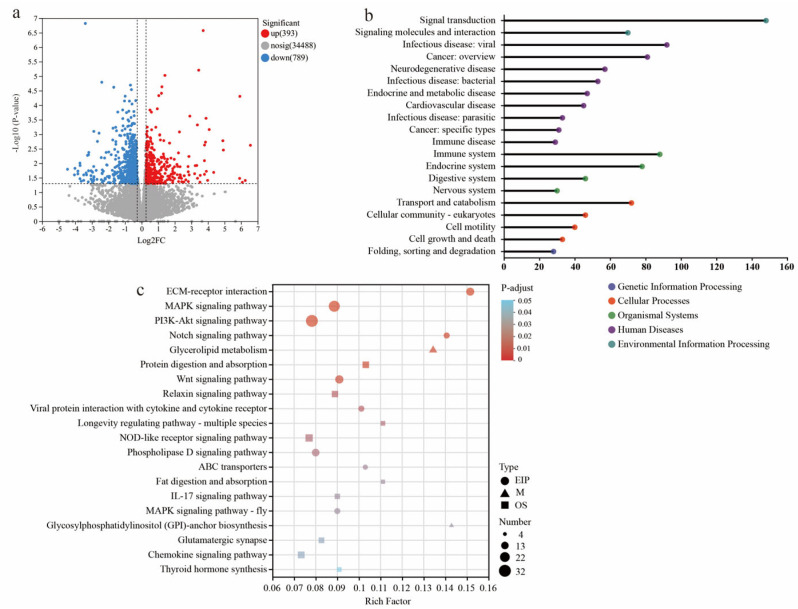
Transcriptome sequencing analysis of cecal mucosa between NBW and LBW-CON groups. (**a**) The volcano plot of transcriptional analysis shows the distribution of differentially expressed genes (DEGs). Red and blue points correspond to 1.2-fold changes in the two groups (*p* < 0.05, LBW-CON versus NBW, *n* = 5). KEGG annotation (**b**) and KEGG pathway enrichment (**c**) analyses of DEGs are presented.

Compared to NBW piglets, the KEGG functional annotation analysis revealed that the differentially expressed genes (DEGs) in LBW piglets were mainly enriched in pathways related to signal transduction, viral and bacterial infections, and the immune system (Figure 4a,b; see Table S8 for the detailed list of DEGs). Functional analyses also suggested perturbations in cellular processes including transport and catabolism, cellular community-eukaryotes, cell motility, and cell growth and death. The pathway enrichment revealed DEGs are mainly involved in environmental information processing, especially in the extracellular matrix (ECM)–receptor interaction, MAPK signaling pathway, PI3K-Akt signaling pathway, Notch signaling pathway, and Wnt signaling pathway (Figure 4c). These pathways are intricately associated with the maintenance of gut barrier integrity.

**Figure 5:**
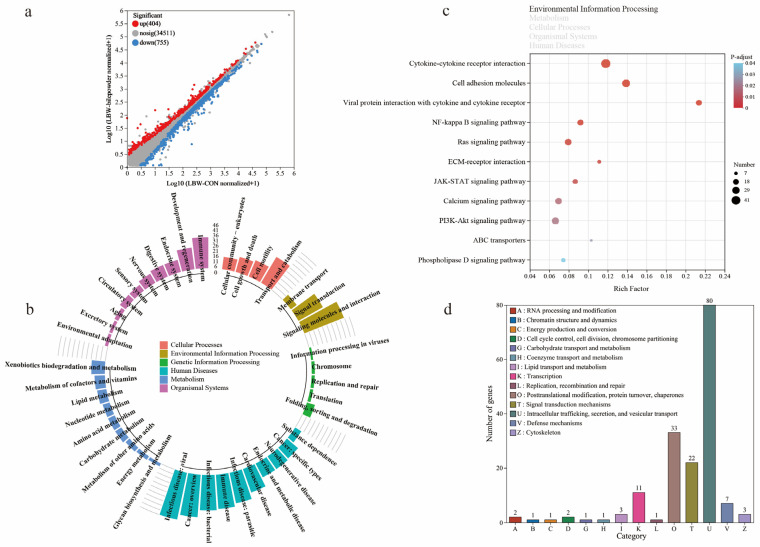
Transcriptome sequencing analysis of cecal mucosa between LBW-CON and LBW-bile powder groups. (**a**) The scatter plot of transcriptional analysis shows the distribution of differentially expressed genes (DEGs). Red and blue points correspond to 1.2-fold changes in the two groups (*p* < 0.05, LBW-bile powder versus LBW-CON, *n* = 5). KEGG annotation (**b**) and KEGG pathway enrichment (**c**) analyses of DEGs, specifically highlighting the primary category of environmental information processing pathways, and functional classification results of eggNOG annotation (**d**) are presented.

On the other hand, the DEGs in cecal mucosa between LBW-bile powder and LBW-CON groups were mostly categorized into viral infectious pathways, immune response pathways, and signaling molecules and interaction (Figure 5a,b; see Table S9 for the detailed list of DEGs). As signaling molecules, BAs have a direct effect on the gut environment through modulating immune responses, as well as maintaining mucosal epithelial barrier function. Based on the above findings, environmental information processing was identified as a key factor in distinguishing between gut barrier dysfunction and homeostasis. Among these pathways, the DEGs between LBW-bile powder and LBW-CON groups were primarily enriched in cytokine–cytokine receptor interaction, cell adhesion molecules, viral protein interaction with cytokine and cytokine receptors, etc. Interestingly, numerous of these genes involved intracellular trafficking, secretion, and vesicular transport pathways (Figure 5c,d).

### 3.5. Potential Mechanisms Underlying Intestinal Barrier Function Alterations in LBW Piglets

**Figure 6:**
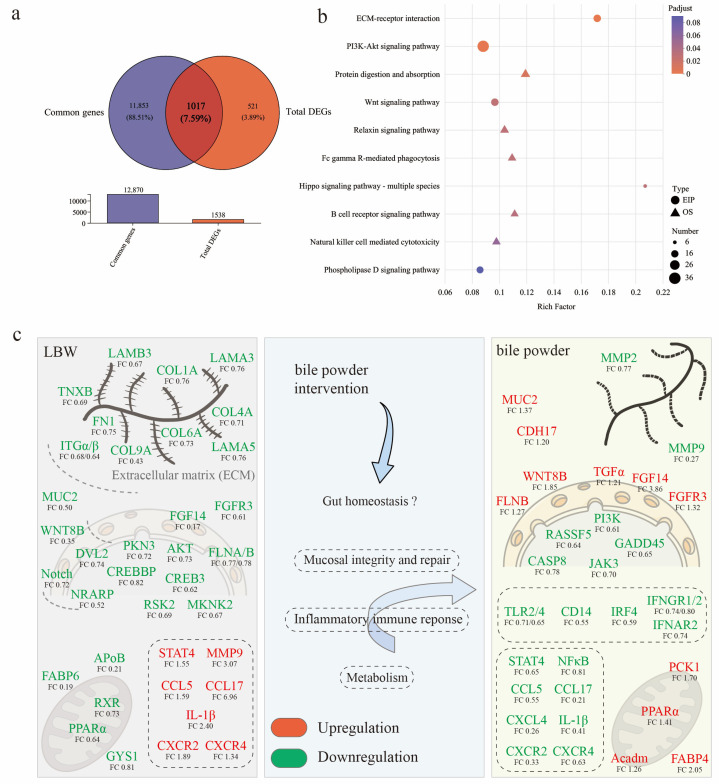
Comparison of differentially expressed genes (DEGs) among the three groups. (**a**) Venn diagram of the overlap between the common genes and total DGEs. (**b**) KEGG enrichment analysis of DEGs identified in the three groups. (**c**) The diagram illustrates potential molecular mechanisms driving intestinal barrier function alterations observed in LBW piglets. Note, the positive regulation (red) and negative regulation (green) of genes are represented by colors in the figure to illustrate the changes in gene expression under different regulatory states. Note: AKT, protein kinase B; APoB, apolipoprotein B; Acadm, acyl-CoA dehydrogenase medium chain; CASP8, caspase 8; CCL, C-C motif chemokine ligand; CDH17, cadherin 17; COL1A, collagen type I alpha 1 chain; CREB3, cAMP responsive element binding protein 3; CXCR, C-X-C motif chemokine receptor; DVL2, dishevelled segment polarity protein 2; FABP, fatty acid binding protein; FGF14, fibroblast growth factor 14; FGFR3, fibroblast growth factor receptor 3; FLNA, filamin A; FN1, fibronectin 1; GADD45, growth arrest and DNA damage inducible; GYS1, glycogen synthase 1; IFNGR, interferon gamma receptor; IL-1β, interleukin-1β; IRF4, interferon regulatory factor 4; ITGα, integrin subunit alpha; JAK3, janus kinase 3; LAMA5, laminin subunit 5; MKNK2, MAPK interacting serine/threonine kinase 2; MMP9, matrix metallopeptidase 9; MUC2, mucin 2; NF-κB, nuclear factor-kappa B; NRAPR, Notch regulated ankyrin repeat protein; PCK1, phosphoenolpyruvate carboxykinase 1; PI3K, phosphoinositide 3-kinase; PKN3, protein kinase N3; PPAR, peroxisome proliferator activated receptor; RASSF5, Ras association domain family member 5; RSK2, ribosomal protein S6 kinase A3; RXR, retinoid x receptor; STAT4, signal transducer and activator of transcription 4; TGFα, transforming growth factor alpha; TLR, toll like receptor; TNXB, tenascin XB; WNT8B, Wnt family member 8B.

As shown in Figure 6a,b, KEGG enrichment analysis was conducted using the intersecting genes between the common genes and total DEGs across the three groups, to investigate the regulatory role of HCAs on the cecal mucosa of LBW piglets. This transcriptomic analysis further revealed key molecular pathways potentially involved in the altered intestinal barrier function observed in LBW piglets (Figure 6c, mainly references the significantly enriched KEGG pathways). For instance, the expressions of multiple genes related to ECM (including *FN1*, *COL1A*, *COL6A*, etc.), and matrix metalloproteinases (such as *MMP9*) were altered, suggesting impaired ECM structure and intercellular adhesion function. Downregulation of laminin subunits (*LAMA3*, *LAMA5*, and *LAMB3*) indicated disrupted cell–matrix interactions. Mucin 2 (*MUC2*), a critical component of the intestinal mucus barrier, was significantly downregulated in LBW piglets, further highlighting mucosal barrier dysfunction. In addition, inflammatory dysregulation was implied through upregulation of *CCL5*, *IL-1β*, *CXCR2*, *CXCR4*, and *STAT4*, alongside abnormalities in lipid and energy metabolism-related genes, including *PPARα*, *RXR*, *FABP6*, etc. HCAs supplementation ameliorated these changes, reducing inflammation-related gene expression (such as *TLR4*, *CXCL4*, *CCL5*, *IL-1β*) and enhancing lipid metabolism through upregulation of *PPARα*, *FABP4*, *PCK1*, and *Acadm*. Notably, the restored expression of *MUC2* was observed in LBW piglets, suggesting improved mucus barrier integrity. Moreover, reduced expression of matrix metalloproteinase genes (*MMP-2*, *MMP-9*) suggested ECM repair and improved barrier integrity. Overall, HCAs mitigated the mucosal abnormalities in LBW piglets, partially restoring ECM remodeling, mucus barrier function, and metabolic homeostasis.

### 3.6. Supplementation of HCAs Improves Epithelial Barrier Function in the Cecal Mucosa of LBW Piglets

**Figure 7:**
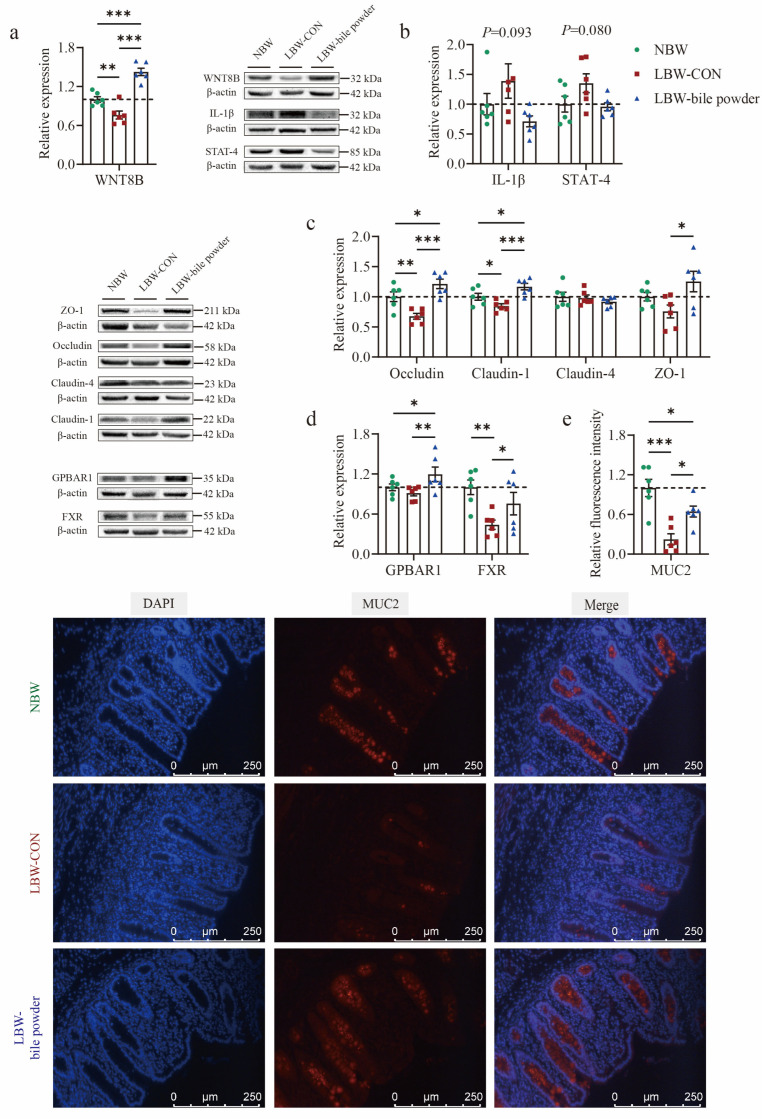
Hyocholic acid species (HCAs) improve cecal mucosa barrier function in low-birth-weight piglets. Proteins expression of (**a**) Wnt family member 8B (WNT8B); (**b**) interleukin-1β (IL-1β) and signal transduction and activator of transcription-4 (STAT-4); (**c**) bile acid main receptors G protein-coupled bile acid receptor (GPBAR1) and farnesoid x receptor (FXR); (**d**) tight junction proteins including Occludin, Claudin-1, Claudin-4, and zonula occludens-1 (ZO-1). (**e**) Representative immunofluorescence images of mucin 2 (MUC-2, red) and the nuclei stained with DAPI (blue). Full-length blots are presented in the Supporting Information, *n* = 6 per group. * *p* < 0.05, ** *p* < 0.01, *** *p* < 0.001.

Subsequently, the cecal mucosa underwent Western blot analysis to assess the expression level of representative proteins among the three groups. Wnt family member 8B (WNT8B), a canonical Wnt ligand involved in various developmental processes, was found to be downregulated in the cecal mucosa of LBW piglets (*p* < 0.01; Figure 7a). Protein expressions of IL-1β and STAT4 showed an increasing tendency in the LBW-CON group (*p* = 0.093 and *p* = 0.080, respectively; Figure 7b). The downregulation of tight junction proteins, including Occludin (*p* < 0.01) and Claudin-1 (*p* < 0.05), was also observed at the protein level in the LBW-CON group (Figure 7c). Notably, increasing evidence has shown that BAs are critical in regulating the intestinal epithelial barrier, cell death, and mucus secretion [32]. Among the main receptors involved, FXR was markedly downregulated in the LBW-CON group (*p* < 0.01; Figure 7d). Its function appeared to be partially restored following the administration of BAs (*p* < 0.05). In addition, HCAs supplementation led to the upregulation of the major membrane receptor GPBAR1 for BAs, (*p* < 0.05). These changes were accompanied by significantly upregulated WNT8B protein expression (*p* < 0.001), as well as restored both Occludin and Claudin-1 protein expression (*p* < 0.001). LBW piglets supplemented with HCAs also significantly improved the level of ZO-1 protein expression compared to LBW-CON piglets. The cecal tissues were further subjected to immunofluorescence staining for DAPI (blue) to label nuclei and MUC2 (red) to visualize the goblet cell marker mucin 2 in the cecal mucosa. Representative images are shown in Figure 7e. Quantitative analysis of fluorescence intensity revealed a strong MUC2 signal within the cecal mucosa of the NBW and LBW-bile powder groups, whereas the LBW-CON group displayed a markedly weaker signal (*p* < 0.001).

In sum, these findings indicate that the supplementation of HCAs can significantly improve gut barrier function and alleviate the negative consequences associated with developmental delays in the cecal mucosa of LBW piglets.

## 4. Discussion

Neonatal LBW piglets are characterized by higher mortality rates and damaged intestinal morphology and function, which together impede their growth and increase their vulnerability to infections. According to several studies, LBW pig neonates exhibit signs of delayed development in both intestinal barrier and immune system structures at approximately one week of age [33,34]. In the present study, we initially observed obvious morphological alterations in the cecum, which were concomitant with a distinct intestinal BA profile between LBW and healthy piglets. This may also imply that the specific BAs play a key role in maintaining gut homeostasis in the neonatal period. Generally, BAs are cholesterol derivatives composed of four steroid rings arranged in a hydrocarbon lattice, featuring hydrophobic convex and hydrophilic concave faces. Their acidic five-carbon side chains are amidated with taurine or glycine [32]. This amphipathic structure grants BAs detergent-like properties, allowing them to form micelles. It is classically posited that BAs play a significant role in facilitating nutrient absorption [35]. Importantly, BAs serve as endogenous ligands that regulate energy metabolism and immune function via the main receptors, including FXR and GPBAR1 (also known as TGR5) [13]. Its function also includes regulating gut epithelial barrier, cell death and proliferation, and mucus secretion [30]. Following the administration of HCAs via oral gavage in LBW piglets, a significant increase in cecal length and mucosal thickness was observed, effectively restoring cecal development to a healthy state.

BAs are typically secreted into the intestinal lumen and continuously transformed into unconjugated forms by gut microbiota, which is important for gastrointestinal homeostasis [36]. Previous research showed that LBW piglets experience a disruption in the gut micro-ecological balance, disrupting the normal metabolic processes [37]. Our findings also indicated a strong elevation in the microbial dysbiosis index at the genus level in the LBW piglets. As mentioned before, prior research has demonstrated a marked disparity in the intestinal microbiome between LBW and NBW piglets, especially the dominant genus *Lactobacillus*, which was notably deficient in the LBW piglets [19,31]. Although these results appear somewhat like ours, the cecal microbiota composition in this study showed no significant difference between LBW-CON and NBW groups (abundance accounted for 38.92 versus 17.18%). *Lactobacillus* contains bile salt hydrolase (BSH) activity, previously shown to be necessary for producing secondary BA metabolites [36]. The relative abundance of 7α- and 7β-hydroxysteroid dehydrogenase-producing bacteria, such as *Escherichia Shigella*, was also reduced in the LBW-CON group. Thus, alterations in the BA pool, particularly those commonly referred to as secondary BAs derived from microbiota, in the gut have been linked to dysbiosis.

Our results indicated that supplementation of HCAs restored the abundance of *Lactobacillus* to 31.59% in the cecum of LBW piglets. The abundances of *Romboutsia* and *Clostridium sensu stricto 1*, both of which are closely associated with the production of SCFAs [38], were also significantly elevated in the LBW-bile powder group. Recent study has demonstrated that *Clostridium butyricum* SLZX19-05, a novel strain classified under *Clostridium sensu stricto 1* and isolated from Tibetan piglets, significantly enhances gut morphology, immune defense, and barrier function in neonatal pigs during the weaning period [39]. Combined with these results in Figure 3b,f, the supplementation of HCAs in LBW piglets appeared to mitigate the between-group differences in diversity and restored the microbial dysbiosis index of cecal microbiota, rendering it comparable to that observed in healthy piglets. These findings initially suggest that BAs especially HDCA play a crucial role in shaping gut microbial ecology and directly or indirectly influence host health [14,40,41].

The gut microbiota is known to modulate host intestinal homeostasis, partly through metabolic pathways including SCFA and BA metabolism [42]. SCFAs mainly include acetate, propionate, butyrate, etc., and their role is now well recognized in the association between the gut microbiota and host [43]. It has previously been reported that LBW piglets showed decreased colonic SCFA content at the post-weaning stage [44]. However, our results showed that supplementation of HCAs did not affect total SCFA concentrations in the cecum of LBW piglets. In neonatal mice infected with *Escherichia coli*, UDCA was found to inhibit bacterial growth and invasion processes, resulting in improved hindgut microflora structure and SCFA production [45]. Another study demonstrated that dietary bile extract supplementation in LBW piglets positively influenced colonic microbial ecology and epithelial integrity, but it resulted in reduced concentrations of butyrate, propionate, and total SCFA [46]. The possible reason for the discrepancy is that 1) the bacteria lack fermentative substrates, e.g., dietary fiber and indigestible oligosaccharides, due to the absence of solid food ingestion for these suckling piglets in the neonatal stage; and 2) the concentrations of SCFA within the gut lumen are influenced by microbial synthesis and the metabolic activity of gut epithelial cells.

Besides this, HCAs classified as non-12-OH BAs are the predominant BAs in pigs and are also present in trace amounts in humans [47]. Notably, HCAs were observed to be lacking in cecum of LBW piglets. This BA species has previously been indicated to simultaneously modulate FXR and GPBAR1 in enteroendocrine L-cells, a unique mechanism not observed in other BA species [48]. In addition, the receptor GPBAR1 has recently gained increasing attention due to its strong affinity for secondary BAs [49,50]. For instance, patients with ulcerative colitis exhibit a reduction in endogenous levels of secondary BAs and that reconstitution of physiological levels of BAs by rectal administration of LCA mitigated murine colitis through a mechanism involving GPBAR1-dependent mitigation actions [51]. Although its role is well established in particular subpopulations of the intestinal epithelium, such as in the enteroendocrine L-cells and immune regulatory cells, the involvement of the BA-GPBAR1 axis in maintaining the physical barrier formed by gut epithelial cells and their tight junction binding, particularly in LBW neonates, remains largely unknown. Importantly, a prior work utilizing Lgr5-Cre-driven GPBAR1 conditional knockout mice revealed that intestinal stem cells exhibited impaired self-renewal, reduced differentiation capacity, and delayed post-colitis regeneration, accompanied by disrupted YAP/SRC signaling pathway activation [52]. This finding directly establishes GPBAR1 as a key secondary BA—driven mediator of epithelial repair through the regulation of intestinal stem cell activity. Herein, our results demonstrate that HCAs supplementation upregulated the GPBAR1 expression in the cecal mucosa. Herein, our results demonstrate that HCAs significantly upregulated GPBAR1 expression and restored FXR expression in the cecal mucosa of LBW piglets.

Intestinal epithelial cells are affected by their microenvironment through multiple mechanisms that govern their proliferation, survival, and differentiation [53,54]. The basement membrane plays a critical role in maintaining cellular homeostasis by modulating the availability of growth factors and other signaling molecules [55]. Based on transcriptome analysis of the cecal mucosa between LBW and NBW piglets, the pathway enrichment revealed the differentially expressed genes are mainly involved in environmental information processing, e.g., ECM–receptor interaction, MAPK signaling pathway, PI3K-Akt signaling pathway, Notch signaling pathway, and Wnt signaling pathway. These results indicate that the intestinal integrity of LBW piglets may be compromised. For instance, the expressions of multiple genes related to ECM (such as *FN1*, *COL1A*, *COL6A*, etc., downregulation) and matrix metalloproteinases (such as *MMP9*, upregulation), laminin subunits (such as *LAMA3*, *LAMA5*, *LAMB3*, downregulation) were changed in cecal mucosa of LBW piglets, suggesting that abnormal interactions between cells and matrix. Among these, the loss of LAMA5 within the epithelium leads to defects in cell adhesion and organization [56]. Another study observed an upregulation of caspase-3 and a downregulation of proliferating cell nuclear antigen (PCNA) in the small intestine of weaned LBW piglets [20]. Here, we found that WNT8B was downregulated while STAT-4 exhibited a tendency to increase in the cecum.

Previous work has revealed that the classical CYP8B1-CA metabolic pathway is significantly activated in both ulcerative colitis patients and experimental colitis mouse models [30]. Notably, in vitro supplementation of CA was found to downregulate key transcripts associated with the cell cycle, cellular differentiation, and Wnt signaling pathways. In contrast, HCA species, a subclass of non-12-OH BAs, have exhibited distinct beneficial effects on ECM remodeling, mucus barrier function, and metabolic homeostasis. Environmental information processing was mentioned as a critical factor differentiating gut barrier dysfunction or homeostasis. Between LBW-CON and LBW-bile powder groups, the majority of differentially expressed genes were associated with intracellular trafficking, secretion, and vesicular transport pathways. These pathways are essential for the efficient processing and transport of proteins, lipids, and other molecules across epithelial cells, contributing to the defense mechanisms and overall homeostasis of the intestinal mucosa [57]. Following the oral gavage of HCAs, a strong upregulation of the WNT8B at both transcript and protein levels was found in the cecal mucosa of LBW piglets, suggesting that HCAs are involved in the modulation of intestinal development, and the differentiation and proliferation of intestinal epithelial cells. Furthermore, the supplementation of HCAs markedly enhanced mucosal integrity and barrier function, as reflected by the restoration of expression levels of MUC2 and tight junction proteins including Occludin, Claudin-1, and ZO-1. This also serves as a validation of our previous findings, in which pectin supplementation in piglets enhanced intestinal tight junction integrity and reduced intestinal hyperpermeability through modulation of the bile acid pool, i.e., an increase in HCAs such as HCA, HDCA, and taurine-conjugated HDCA [28]. While HCAs intervention effectively restored gut homeostasis, the lack of immediate weight gain underscores the need for longer-term investigations to assess its impact on growth trajectories [46]. Future studies should incorporate comprehensive metabolic profiling (e.g., energy expenditure, hormonal regulation) and extended observational periods to better capture potential temporal discrepancies between molecular alterations and phenotypic manifestations. Given that the bile powder used in this study was a natural mixture extracted from porcine bile, it remains unclear which specific BA serves as the key effector mediating the observed improvements in intestinal barrier function and development of LBW piglets. As bile powder supplementation restored FXR expression and upregulated GPBAR1 in the intestines of LBW piglets, further in vitro experiments using intestinal epithelial cells with GPBAR1 or FXR knockdown are warranted to elucidate the specific signaling pathways through which HCA species—particularly HDCA—promote intestinal barrier repair. Moreover, studies using purified individual BAs are needed to delineate their distinct effects and interactions with gut microbiota, ultimately aiding the identification of targeted nutritional interventions for LBW-induced gut dysregulation.

## 5. Conclusions

This study identified a distinct bile acid imbalance in low-birth-weight (LBW) piglets, characterized by reduced levels of hyocholic acid species (HCAs), which were associated with impaired cecal development and barrier function. Oral supplementation with HCAs during the neonatal period improved cecal morphology and partially restored gut barrier integrity, potentially via modulation of the key bile acid-activated receptors and microbial composition. These findings highlight the potential of targeted bile acid restoration as a nutritional strategy to mitigate gut dysfunction in low-birth-weight neonates.

## Figures and Tables

**Table 1:** Comparison of body weights of piglets between low- and normal-birth-weight piglets.

Items (Unit: kg)	Groups			*p*-Value
	NBW	LBW-CON	LBW-Bile Powder	
Initial BW	1.563 ± 0.052 ^a^	0.950 ± 0.120 ^b^	0.925 ± 0.116 ^b^	*p* < 0.0001
BW-7d	2.600 ± 0.177 ^a^	1.550 ± 0.267 ^b^	1.600 ± 0.293 ^b^	*p* < 0.0001
BW-14d	4.375 ± 0.410 ^a^	3.013 ± 0.533 ^b^	3.513 ± 0.673 ^b^	*p* = 0.0003
BW-21d	6.300 ± 0.760 ^a^	4.338 ± 0.703 ^b^	4.538 ± 0.769 ^b^	*p* < 0.0001
ADG	0.264 ± 0.052 ^a^	0.199 ± 0.039 ^b^	0.210 ± 0.039 ^b^	*p* = 0.016

Note: average daily gain (ADG) refers to the period from day 7 to 21. All data are expressed as mean ± SD (*n* = 8 per group). Values with different lowercase letter superscripts within the same row indicate a significant difference (*p* < 0.05).

**Table 2:** The alteration in short-chain fatty acid (SCFA) contents in the cecum.

Items (Unit: μmol/g)	Groups			*p*-Value
	NBW	LBW-CON	LBW-Bile Powder	
Acetic acid	145.212 ± 85.099	108.132 ± 23.934	90.955 ± 36.764	*p* = 0.159
Propionic acid	26.678 ± 16.485	24.071 ± 6.685	19.564 ± 8.656	*p* = 0.465
Butyric acid	15.542 ± 8.733	12.171 ± 4.342	13.238 ± 6.264	*p* = 0.596
Isovaleric acid	6.805 ± 4.207	6.109 ± 1.527	4.755 ± 1.840	*p* = 0.347
Valeric acid	6.082 ± 4.420	5.127 ± 1.243	4.406 ± 1.562	*p* = 0.498
Total SCFAs	200.319 ± 118.062	155.610 ± 34.882	132.918 ± 53.471	*p* = 0.232

All data are expressed as mean ± SD (*n* = 8 per group).

## Data Availability

The RNA sequencing (RNA-seq) data generated in this study have been archived in the NCBI Sequence Read Archive (SRA) database under the Bio Project accession number PRJNA1171435 for the piglet cecal mucosa. Additionally, the raw sequencing data of the gut microbiota in the cecal mucosa are accessible under the accession number PRJNA1171211.

## Supplementary Materials

The following supporting information can be downloaded at: https://www.mdpi.com/article/10.3390/nu17213415/s1, Table S1: Ingredient composition of bile powder. Table S2: Primers used for quantitative real-time PCR assay. Table S3: Cecal bile acid profiles in LBW and NBW piglets. Table S4: Contents of bile acid category in LBW and NBW piglets. Table S5: The alterations in cecum length and thickness among the three groups. Table S6: Contents of the bile acid category among the three groups. Table S7: Cecal bile acid profiles among the three groups. Table S8: List of differentially expressed genes (DEGs) between the LBW and NBW groups. Table S9: List of differentially expressed genes (DEGs) between the LBW-bile powder and LBW-CON groups.

## Abbreviations

BA	bile acid
CYP7A1	cholesterol 7 alpha-hydroxylase
CYP7B1	oxysterol 7 alpha-hydroxylase
CYP8B1	sterol 12 alpha-hydroxylase
CYP27A1	sterol 27-hydroxylase
CYP4A21	taurochenodeoxycholic acid 6alpha-hydroxylase
DEGs	differentially expressed genes
ECM	extracellular matrix
FXR	farnesoid x receptor
GCBA	glycine-conjugated bile acid
GC-MS	gas chromatography–mass spectrometry
GPBAR1	G-protein-coupled bile acid receptor 1
HCA	hyocholic acid
HDCA	hyodeoxycholic acid
LBW	low birth weight
LCA	lithocholic acid
LC-MS/MS	liquid chromatography–tandem mass spectrometry
MUC2	mucin 2
NBW	normal birth weight
OTU	operational taxonomic unit
PBA	primary bile acid
PCoA	principal coordinate analysis
SBA	secondary bile acid
SCFA	short-chain fatty acid
TCBA	taurine-conjugated bile acid
UDCA	ursodeoxycholic acid
WNT8B	Wnt family member 8B
ZO-1	zonula occluden-1
