# Supplementary material for: Hyocholic Acid Species as the Key Modulator for Cecal Epithelial Homeostasis in Low-Birth-Weight Piglets
Source: Nutrients. 2025 Oct 30;17(21):3415. doi: 10.3390/nu17213415 (PMC12608154; doi:10.3390/nu17213415)
Supplement: Supplementary file 1 [file nutrients-17-03415-s001.zip › nutrients-3906552-supplementary.pdf]

Table S1. Ingredient composition of bile powder.

Ingredients（mg/g)	replicate 1	replicate 2
12-Ketolithocholic_acid_KLCA	0.000	1.056
6,7-Diketolithocholic_acid_DiketoLCA	0.064	0.000
7,12-Diketolithocholic_acid_DiketoLCA	0.000	0.000
7-Ketolithocholic_acid_7-KLCA	0.551	0.453
IsoalloLCA	0.000	0.000
Chenodeoxycholic_acid_CDCA	20.583	15.723
Cholic_acid_CA	0.133	0.110
Deoxycholic_acid_DCA	0.000	0.000
Glycochenodeoxycholic_acid_GCDCA	0.213	0.181
Glycocholic_acid_GCA	0.000	0.000
Glycodeoxycholic_acid_GDCA	0.000	0.000
Glycohyocholic_ acid_ GHCA	0.000	0.000
Glycohyodeoxycholic_acid_GHDCA	0.203	0.196
Glycolithocholic_acid_GLCA	0.049	0.025
Glycoursodeoxycholic_acid_GUDCA	0.000	0.000
Hyocholic_acid_HCA	67.951	58.390
Hyodeoxycholic_acid_HDCA	909.631	815.584
Isodeoxycholic_acid_isoDCA	0.000	0.000
Isolithocholic_acid_isoLCA	0.000	0.000
Lithocholic_acid_LCA	0.228	0.189
Taurochenodexycholic_acid_TCDCA	0.000	0.000
Taurocholic_acid_TCA	0.000	0.000
Taurodeoxycholic_acid_TDCA	0.000	0.000
Taurohydrocholic_acid_THCA	0.000	0.001
Taurolithocholic_acid_TLCA	0.000	0.000
Tauroursodexycholic_acid_TUDCA	0.046	0.045
Tauro-α-muricholic_acid_Tα MCA	0.001	0.000
Tauro-β-muricholic_acid_Tβ MCA	0.000	0.000
Tauro-ω-muricholic_acid_Tω MCA	0.000	0.000
Ursodeoxycholic_acid_UDCA	0.032	0.026
α-Muricholic_acid_MCA	0.033	0.030
β-Muricholic_acid_MCA	0.000	0.000
ω-Muricholic_acid_ MCA	0.416	0.362

Note: qualitative analysis of the ingredient composition of bile powder using liquid chromatography-mass spectrometry (LC-MS).

Table S2. Primers used for quantitative real-time PCR assay.

Genes	Accession No.	Primer sequences
FXR	NM_001287412.1	F：TGAGCTTTGTGTCGTTTGCG<br>R：ACATTCAGCCAACATTCCCA
Cyp7a1	NM_001005352.3	F：GAAAGAGAGACCACATCTCGG<br>R：GAATGGTGTTGGCTTGCGAT
Cyp8b1	NM_214426.1	F：CCGGAAGAATATGTTGGAAT<br>R：AAGTCTAGTTTTCTCTTCGC
Cyp27a1	NM_001243304.1	F：TCGAAGTTGGTGGCTTCCTC<br>R：ACTGCTGGATCAGCCTTGTC
Cyp7b1	AK233030	F：CTGCGGAAGGGAGACTTTGT<br>R：GCCCGGACATTTGCTGATTC
Cyp4a21	NM_214425.1	F：TTTCCCGCTTGAGGAGTGCC<br>R：CAGCTGGATCACTCGGTCTG
SHP	AH014861.3	F：CCCCAAGGAATACGCCTACC<br>R：ACAACGGGTGTCAAGCCTTT
FGFR4	XM_013987555.2	F：GCTCAGAGGTGGAGGTCCTA<br>R：GCCTGCCAGACAGGTGTATT

Table S3. Cecal bile acid profiles in LBW and NBW piglets.

Ingredients (ng/g)	Groups		*P*-value
	NBW	LBW	
CA	150.062±138.834	154.532±219.397	*P*=0.962
GCA	5.931±7.795	14.778±15.647	*P*=0.174
TCA	108.795±32.508[b]	395.804±338.070[a]	*P*=0.032
CDCA	1123.002±733.684	645.610±649.477	*P*=0.190
GCDCA	197.502±117.316	372.069±198.602	*P*=0.050
TCDCA	2328.649±949.146[a]	876.650±896.525[b]	*P*=0.007
HCA	8084.9822±3358.326[a]	1881.223±1190.962[b]	*P*=0.0002
GHCA	576.644±353.774	353.244±283.392	*P*=0.185
THCA	2625.885±1255.351[a]	1106.821±1006.266[b]	*P*=0.018
HDCA	5326.998±3417.582	2317.862±2433.317	*P*=0.062
GHDCA	144.498±120.915	225.593±326.564	*P*=0.521
IsoDCA	524.905±373.255	511.628±922.403	*P*=0.970
LCA	10958.992±13588.969	14432.581±14594.628	*P*=0.630
GLCA	170.869±160.114	66.580±63.796	*P*=0.109
TLCA	54.214±49.375	90.692±65.249	*P*=0.228
12-ketoLCA	188.615±99.057	235.032±282.674	*P*=0.668
6,7-diketoLCA	184.197±113.366	129.261±74.212	*P*=0.271
7-ketoLCA	195.035±153.884	287.748±467.832	*P*=0.603
IsoalloLCA	2829.692±2469.953	2388.841±2375.840	*P*=0.721
IsoLCA	2826.534±3431.082	2687.845±2310.330	*P*=0.926
UDCA	363.606±180.757	1044.630±1686.433	*P*=0.275
TUDCA	1867.807±1560.918	1705.482±1485.857	*P*=0.834
TαMCA	54.057±27.754	39.010±24.437	*P*=0.269
TβMCA	6.788±6.814	3.749±4.077	*P*=0.297
ω-MCA	30.405±48.808	440.706±808.093	*P*=0.174

All data are expressed as mean ± SD (n = 8 per group). Values with different lowercase letter superscripts within the same row indicate a significant difference (*P*<0.05).

Table S4. Content of bile acid category in LBW and NBW piglets.

Ingredients （ng/g)	Groups		*P*-value
	NBW	LBW	
TBA	40928.665±24018.344	32407.968±21125.525	*P*=0.464
PBA	15201.451±4387.251[a]	5800.731±3093.912[b]	*P*=0.0002
SBA	31054.210±21204.237	28925.099±20880.320	*P*=0.843
HCAs	16759.006±5982.953[a]	5884.741±3695.900[b]	*P*=0.0006
TCBA	7046.194±2841.245	4281.208±2798.821	*P*=0.065
GCBA	1095.444±608.825	1032.264±670.879	*P*=0.847

All data are expressed as mean ± SD (n = 8 per group). Values with different lowercase letter superscripts within the same row indicate a significant difference (*P*<0.05).

Table S5. The alterations in cecum length and thickness among the three groups.

Items	Groups			*P*-value
	NBW	LBW-CON	LBW-bile powder	
Cecum length, cm	8.688±1.361[a]	6.875±1.157[b]	8.625±1.026[a]	*P*=0.009
Mucosa, μm	305.678±31.512[a]	258.841±44.398[b]	314.246±38.934[a]	*P*=0.020
Submucosa, μm	263.345±93.146	281.488±67.290	280.540±59.895	*P*=0.862
Muscularis propria, μm	305.455±123.315	269.505±82.619	256.874±91.421	*P*=0.613
Tissue thickness, μm	874.476±196.080	809.834±147.581	851.656±128.974	*P*=0.719

All data are expressed as mean ± SD (n = 8 per group). Values with different lowercase letter superscripts within the same row indicate a significant difference (*P*<0.05).

Table S6. Content of the bile acid category among the three groups.

Ingredients（μg/g)	Groups			*P*-value
	NBW	LBW-CON	LBW-bile powder	
TBA	1650.460±536.653	1860.666±595.223	3443.029±723.278	*P*=0.1086
PBA	1183.885±333.913	1323.154±430.609	1079.889±287.412	*P*=0.8895
SBA	466.575±214.404[b]	537.514±181.667[b]	2363.140±528.056[a]	*P*=0.0010
HCAs	1208.653±352.900[b]	1165.696±308.791[b]	2355.481±442.150[a]	*P*=0.0572
TCBA	324.370±132.328	360.595±161.884	667.718±218.542	*P*=0.3099
GCBA	461.709±231.673	192.486±84.828	841.175±267.513	*P*=0.1146

All data are expressed as mean ± SD (n = 8 per group). Values with different lowercase letter superscripts within the same row indicate a significant difference ($P<0.05$).

Table S7. Cecal bile acid profiles among the three groups.

Ingredients (μg/g)	Groups			*P*-value
	NBW	LBW-CON	LBW-bile powder	
CA	6.818±2.118	5.073±1.713	4.230±1.849	*P*=0.6243
GCA	1.325±0.667	0.291±0.128	0.720±0.174	*P*=0.2167
TCA	2.420±1.221	1.120±0.474	0.465±0.245	*P*=0.2117
CDCA	81.142±29.864	214.199±86.695	179.024±49.033	*P*=0.2887
GCDCA	72.163±46.721	95.578±57.189	170.319±76.528	*P*=0.5092
TCDCA	160.419±70.994	246.938±125.84	215.943±72.260	*P*=0.8032
HCA	577.228±133.967	697.916±170.477	425.365±92.055	*P*=0.4269
GHCA	223.714±92.154	40.498±15.622	69.628±21.784	*P*=0.0633
THCA	58.658±17.656	39.543±15.257	14.194±3.034	*P*=0.0908
HDCA	184.948±64.453[b]	350.532±113.418[b]	1249.159±239.799[a]	*P*=0.0002

GHDCA	164.104±106.469[b]	55.206±23.256[b]	597.136±193.118[a]	*P*=0.0166
GDCA	0.304±0.164	0.295±0.186	0.659±0.409	*P*=0.5762
TDCA	0.725±0.298	0.659±0.357	1.235±0.643	*P*=0.6288
IsoDCA	0.581±0.192	1.294±0.834	2.149±0.943	*P*=0.3392
LCA	0.764±0.528	1.663±0.501	1.483±0.410	*P*=0.3952
GLCA	0.096±0.044[b]	0.619±0.204[a]	0.413±0.115[ab]	*P*=0.0436
TLCA	1.084±0.452	2.803±1.486	2.814±1.207	*P*=0.4763
12-ketoLCA	0.863±0.332	2.826±1.177	0.986±0.339	*P*=0.1299
6,7-diketoLCA	0.033±0.022[b]	0.358±0.125[a]	0.149±0.084a[b]	*P*=0.0486
7-ketoLCA	2.140±1.055[b]	29.816±9.548[a]	22.613±9.112[ab]	*P*=0.0478
IsoalloLCA	0.046±0.046	0.125±0.065	0.041±0.019	*P*=0.3929
IsoLCA	0.066±0.156	0.023±0.007	0.028±0.006	*P*=0.5846
UDCA	0.318±0.092	1.808±1.144	9.160±5.687	*P*=0.1607
TUDCA	100.486±52.021[b]	68.836±29.710[b]	442.643±169.313[a]	*P*=0.0338
αMCA	3.920±1.378	9.306±4.414	10.893±2.656	*P*=0.2665
TαMCA	0.519±0.141	0.613±0.282	0.326±0.131	*P*=0.5863
TβMCA	0.048±0.024	0.036±0.018	0.035±0.012	*P*=0.8711
ω-MCA	5.483±1.696	10.288±3.491	18.373±11.152	*P*=0.4167

All data are expressed as mean ± SD (n = 8 per group). Values with different lowercase letter superscripts within the same row indicate a significant difference (*P*<0.05).

Table S8. List of differentially expressed genes (DEGs) between the LBW and NBW groups.

Gene ID	Gene Name	FC	Log2FC	Pvalue	Padjust	Significant	Regulate
ENSSSCG00000000006	PPARA	0.640815736	-0.64201852	0.011149834	0.617982236	yes	down
ENSSSCG00000000024	PARVB	0.665603873	-0.587264267	0.00425934	0.475451096	yes	down
ENSSSCG00000000111	BAIAP2L2	0.631703199	-0.662681216	0.019117907	0.67109181	yes	down
ENSSSCG00000000116	POLR2F	1.304180871	0.383143964	0.026790381	0.69011278	yes	up
ENSSSCG00000000118	MICALL1	0.731907697	-0.450266377	0.024273343	0.67597251	yes	down
ENSSSCG00000000145	MYH9	0.802050617	-0.318234808	0.040894746	0.715680171	yes	down
ENSSSCG00000000206	FAIM2	0.612732791	-0.706670033	0.007069984	0.539617246	yes	down
ENSSSCG00000000258	RARG	0.744276607	-0.426089204	0.024880221	0.67891626	yes	down
ENSSSCG00000000260	SOAT2	0.677790092	-0.561089548	0.029733597	0.697179556	yes	down
ENSSSCG00000000263	TNS2	0.770155982	-0.376777426	0.027666256	0.693082926	yes	down
ENSSSCG00000000359	BLOC1S1	1.249644274	0.321517473	0.027076787	0.690849599	yes	up
ENSSSCG00000000401	GLS2	1.311722016	0.391462012	0.020220648	0.67109181	yes	up
ENSSSCG00000000517	CAPS2	0.411270695	-1.281839819	0.024350831	0.676100101	yes	down
ENSSSCG00000000584	SLCO1A2	0.127087535	-2.976105556	0.019741932	0.67109181	yes	down
ENSSSCG00000000610	SMCO3	3.905022642	1.965330913	0.007313748	0.551414975	yes	up
ENSSSCG00000000659	A2ML	5.296639517	2.405077324	0.033065335	0.703664106	yes	up
ENSSSCG00000000664	A2ML1	0.301665143	-1.728980092	0.000619103	0.255166648	yes	down
ENSSSCG00000000668	APOBEC1	1.336156059	0.418088521	0.031463324	0.703664106	yes	up
ENSSSCG00000000720	AKAP3	0.674103856	-0.568957217	0.046926375	0.72213448	yes	down
ENSSSCG00000000892	HAL	0.501750725	-0.994957299	0.048669224	0.72213448	yes	down
ENSSSCG00000000968	SBF1	0.741103775	-0.432252521	0.019166	0.67109181	yes	down
ENSSSCG00000000970	PPP6R2	0.718846515	-0.47624433	0.022473468	0.673830277	yes	down
ENSSSCG00000000991	FOXQ1	0.133391262	-2.906263935	0.040498901	0.715680171	yes	down
ENSSSCG00000000999	CDYL	0.778169542	-0.361843581	0.006764782	0.528701837	yes	down
ENSSSCG00000001004	SLC22A23	0.650739942	-0.619846988	0.035468373	0.707740306	yes	down
ENSSSCG00000001021	RREB1	0.748322643	-0.418267665	0.009832539	0.603302839	yes	down
ENSSSCG00000001025	DSP	0.745360483	-0.423989762	0.013919697	0.651736454	yes	down
ENSSSCG00000001041	PAK1IP1	1.230534671	0.299285307	0.019513272	0.67109181	yes	up
ENSSSCG00000001078	MBOAT1	1.287883882	0.365002524	0.036118211	0.707740306	yes	up
ENSSSCG00000001209	ZSCAN12	1.408045488	0.493693942	0.023962791	0.67597251	yes	up
ENSSSCG00000001252	UBD	2.04829677	1.034424757	0.007723466	0.565052652	yes	up
ENSSSCG00000001362	MDC1	0.771757849	-0.373779843	0.008220026	0.578565651	yes	down
ENSSSCG00000001418	NEU1	0.616343826	-0.698192716	0.04661554	0.72213448	yes	down
ENSSSCG00000001422	C2	0.704562207	-0.505201005	0.037166261	0.707740306	yes	down
ENSSSCG00000001427	C4A	0.626313261	-0.67504367	0.022457413	0.673830277	yes	down
ENSSSCG00000001456	MHC2	1.722427007	0.784442846	0.014166463	0.651736454	yes	up
ENSSSCG00000001483	FAM83B	1.300773478	0.379369747	0.033871154	0.703664106	yes	up
ENSSSCG00000001513	SYNGAP1	0.74648765	-0.421809703	0.021309837	0.67241349	yes	down

ENSSSCG00000001518	ITPR3	0.750991687	-0.413131157	0.033784415	0.703664106	yes	down
ENSSSCG00000001549	FKBP5	1.232517582	0.301608227	0.033649814	0.703664106	yes	up
ENSSSCG00000001570	PI16	0.348672066	-1.520057308	0.012982778	0.635895234	yes	down
ENSSSCG00000001595	DAAM2	0.789744477	-0.340542152	0.008741255	0.589351395	yes	down
ENSSSCG00000001596	MOCS1	0.552398285	-0.856219254	0.000989509	0.310915441	yes	down
ENSSSCG00000001619	FOXP4	0.731049989	-0.451958034	0.033839815	0.703664106	yes	down
ENSSSCG00000001657	CUL7	0.679236888	-0.558013283	0.000424823	0.229108458	yes	down
ENSSSCG00000001680	ABCC10	0.779328991	-0.35969561	0.018927467	0.67109181	yes	down
ENSSSCG00000001688	TJAP1	0.732554036	-0.448992914	0.000228782	0.163200506	yes	down
ENSSSCG00000001695	VEGFA	0.698549244	-0.517566274	0.044569301	0.72213448	yes	down
ENSSSCG00000001727	TNFRSF21	0.724213551	-0.465512922	0.000164067	0.133112815	yes	down
ENSSSCG00000001765	ADAMTS7	0.767697068	-0.381390956	0.038801195	0.714516217	yes	down
ENSSSCG00000001865	SCAPER	1.273866767	0.349214395	0.036302414	0.707740306	yes	up
ENSSSCG00000001977	STXBP6	1.249384764	0.321217842	0.020135988	0.67109181	yes	up
ENSSSCG00000001979	GZMB	2.795515201	1.48311419	0.016744717	0.67109181	yes	up
ENSSSCG00000001984	KHNYN	0.81882301	-0.288376451	0.029588659	0.697179556	yes	down
ENSSSCG00000002004	PSME2	1.387746958	0.472744531	0.01816387	0.67109181	yes	up
ENSSSCG00000002026	EFS	0.622777144	-0.683212096	0.016379456	0.67109181	yes	down
ENSSSCG00000002142	TEP1	0.73311491	-0.447888747	0.046994474	0.72213448	yes	down
ENSSSCG00000002276	PLEKHG3	0.798833369	-0.324033497	0.002501321	0.379220559	yes	down
ENSSSCG00000002392	IRF2BPL	0.774625755	-0.368428626	0.008091648	0.578565651	yes	down
ENSSSCG00000002440	CCDC88C	0.735899143	-0.44242004	0.010745777	0.617982236	yes	down
ENSSSCG00000002520	SLC25A20_29	0.661283275	-0.596659682	0.036231755	0.707740306	yes	down
ENSSSCG00000002523	CDC42BPB	0.79909326	-0.323564208	0.029409464	0.696976005	yes	down
ENSSSCG00000002528	TECPR2	0.738318884	-0.437684036	0.045466851	0.72213448	yes	down
ENSSSCG00000002557	NUDT14	0.744748197	-0.42517537	0.019924237	0.67109181	yes	down
ENSSSCG00000002558	CEP170B	0.607185781	-0.719790089	0.010621993	0.617982236	yes	down
ENSSSCG00000002622	TMEM14A	1.597907132	0.676183563	0.005176266	0.513485708	yes	up
ENSSSCG00000002632	SLC28A1	0.135474171	-2.883910273	0.000795973	0.278545508	yes	down
ENSSSCG00000002640	DEF8	0.757962137	-0.399802312	0.031983865	0.703664106	yes	down
ENSSSCG00000002666	KIAA0513	0.707050399	-0.50011504	0.027440154	0.693082926	yes	down
ENSSSCG00000002707	GABARAPL2	1.227206319	0.295377817	0.009730684	0.603302839	yes	up
ENSSSCG00000002716	MLKL	0.817301535	-0.291059651	0.048102741	0.72213448	yes	down
ENSSSCG00000002805	CFAP20	1.208226753	0.272891237	0.00496586	0.513485708	yes	up
ENSSSCG00000002806	MMP15	0.740966702	-0.432519383	0.040005956	0.714830695	yes	down
ENSSSCG00000002937	KRAB	1.340779971	0.423072503	0.046200097	0.72213448	yes	up
ENSSSCG00000002950	SIPA1L3	0.737544595	-0.439197811	0.031387097	0.703664106	yes	down
ENSSSCG00000003024	PRR19	1.630232008	0.705077298	0.049420976	0.725498277	yes	up
ENSSSCG00000003038	GRIK5	0.540436347	-0.887803389	0.005766419	0.523737709	yes	down
ENSSSCG00000003083	CBLC	0.741333299	-0.43180578	0.028516581	0.695002653	yes	down
ENSSSCG00000003092	FOXA3	0.679173052	-0.558148879	0.008664155	0.588622996	yes	down

ENSSSCG00000003103	FKRP	0.751399047	-0.412348809	0.031834156	0.703664106	yes	down
ENSSSCG00000003104	STRN4	0.775065893	-0.367609127	0.020186391	0.67109181	yes	down
ENSSSCG00000003114	DHX34	0.71395824	-0.486088403	0.022300597	0.673830277	yes	down
ENSSSCG00000003146	NTN5	0.713124731	-0.487773658	0.001190811	0.312531581	yes	down
ENSSSCG00000003154	GYS1	0.811179862	-0.301906259	0.031146849	0.703664106	yes	down
ENSSSCG00000003193	TBC1D17	0.777012516	-0.363990257	0.046902771	0.72213448	yes	down
ENSSSCG00000003194	AKT1S1	0.787132275	-0.345321999	0.02285246	0.675846472	yes	down
ENSSSCG00000003195	PNKP	0.797288332	-0.326826539	0.024822933	0.67891626	yes	down
ENSSSCG00000003201	ATF5	0.758888159	-0.398040811	0.01704505	0.67109181	yes	down
ENSSSCG00000003204	VRK3	0.765622705	-0.38529448	0.033015986	0.703664106	yes	down
ENSSSCG00000003214	KCNC3	0.467704165	-1.096331818	0.008849296	0.589351395	yes	down
ENSSSCG00000003218	MYBPC2	0.324223726	-1.624938428	0.003309307	0.445536806	yes	down
ENSSSCG00000003251	NLRP12	3.460404362	1.790940632	0.023448601	0.675846472	yes	up
ENSSSCG00000003272	CD85	2.715737896	1.441344248	0.015309281	0.657271396	yes	up
ENSSSCG00000003329	ACAP3	0.733971604	-0.446203846	0.024686926	0.67891626	yes	down
ENSSSCG00000003334	UBE2J2	1.222492771	0.289825934	0.047673478	0.72213448	yes	up
ENSSSCG00000003338	PUSL1	1.49632672	0.581425219	0.002935406	0.41873231	yes	up
ENSSSCG00000003371	GPR153	0.779955111	-0.358537	0.014713182	0.652664121	yes	down
ENSSSCG00000003379	KLHL21	0.652399336	-0.616172782	2.89E-05	0.059497261	yes	down
ENSSSCG00000003407	CASZ1	0.74647823	-0.421827907	0.046834977	0.72213448	yes	down
ENSSSCG00000003486	ARHGEF10L	0.73448311	-0.445198779	0.043483838	0.72213448	yes	down
ENSSSCG00000003569	SLC9A1	0.769094985	-0.37876631	0.007483153	0.557389706	yes	down
ENSSSCG00000003572	SYTL1	0.527135963	-0.923752975	0.005258885	0.513485708	yes	down
ENSSSCG00000003573	MAP3K6	0.716483558	-0.480994498	0.022699513	0.675846472	yes	down
ENSSSCG00000003590	PTPRU	0.616061734	-0.698853169	0.034712254	0.707740306	yes	down
ENSSSCG00000003592	SDC3	0.783790084	-0.351460774	0.044507824	0.72213448	yes	down
ENSSSCG00000003604	ADGRB2	0.704164297	-0.506016013	0.046610351	0.72213448	yes	down
ENSSSCG00000003645	UTP11	1.251505969	0.323665172	0.007615445	0.562723707	yes	up
ENSSSCG00000003663	HPCAL1	0.78655361	-0.346382995	0.031840339	0.703664106	yes	down
ENSSSCG00000003709	LAMA3	0.761887542	-0.39235003	0.019080999	0.67109181	yes	down
ENSSSCG00000003741	SCAN	1.339216378	0.421389076	0.017157827	0.67109181	yes	up
ENSSSCG00000003779	TYW3	1.370913206	0.455137235	0.046888763	0.72213448	yes	up
ENSSSCG00000003839	PLPP3	0.782004389	-0.35475139	0.003493058	0.447112352	yes	down
ENSSSCG00000003875	TTC39A	0.562084253	-0.831141698	0.001196406	0.312531581	yes	down
ENSSSCG00000003882	SLC5A9	0.451547061	-1.14705174	0.040119501	0.714830695	yes	down
ENSSSCG00000003941	SZT2	0.70014011	-0.514284436	0.022193842	0.673830277	yes	down
ENSSSCG00000004018	AFDN	0.735043272	-0.444098911	0.01794383	0.67109181	yes	down
ENSSSCG00000004020	SFT2D1	1.313600203	0.393526255	0.002782971	0.413957683	yes	up
ENSSSCG00000004022	RPS6KA2	0.690072664	-0.535179812	0.001035759	0.310915441	yes	down
ENSSSCG00000004033	AGPAT4	0.797210487	-0.326967406	0.033730942	0.703664106	yes	down
ENSSSCG00000004044	IGF2R	0.749949337	-0.415134958	0.044040769	0.72213448	yes	down

ENSSSCG00000004093	IYD	0.532095798	-0.910242086	0.029773099	0.697224317	yes	down
ENSSSCG00000004101	LRP11	0.638152147	-0.648027666	0.01463354	0.652664121	yes	down
ENSSSCG00000004170	spindlin 1	1.336200228	0.41813621	0.030577292	0.700958898	yes	up
ENSSSCG00000004340	FHL5	1.327624769	0.408847451	0.041001675	0.715680171	yes	up
ENSSSCG00000004436	COL10A1	2.160614424	1.111441636	0.046009766	0.72213448	yes	up
ENSSSCG00000004506	CTIF	0.71070329	-0.492680717	0.022410678	0.673830277	yes	down
ENSSSCG00000004625	LEO1	1.262474316	0.336254037	0.001161371	0.312531581	yes	up
ENSSSCG00000004670	C15orf48	1.227542537	0.295773018	0.002020344	0.353910414	yes	up
ENSSSCG00000004687	B2M	1.544239538	0.626896556	0.010653822	0.617982236	yes	up
ENSSSCG00000004705	MAP1A	0.792793531	-0.334982904	0.044391087	0.72213448	yes	down
ENSSSCG00000004732	PLA2G4F	0.708947369	-0.496249566	0.03365508	0.703664106	yes	down
ENSSSCG00000004738	MAPKBP1	0.767563742	-0.381641531	0.02367806	0.675846472	yes	down
ENSSSCG00000004774	IVD	0.76407477	-0.388214272	0.020480144	0.67109181	yes	down
ENSSSCG00000004928	CILP	0.422089602	-1.244378807	0.032774478	0.703664106	yes	down
ENSSSCG00000004948	SMAD6	0.628755303	-0.669429432	0.006150593	0.527035893	yes	down
ENSSSCG00000005030	NID2	0.767802015	-0.381193748	0.024348693	0.676100101	yes	down
ENSSSCG00000005083	DHRS7	1.53709907	0.620210154	0.005118793	0.513485708	yes	up
ENSSSCG00000005208	RIC1	1.234396255	0.30380559	0.042875945	0.72213448	yes	up
ENSSSCG00000005291	TLE1	0.762228843	-0.391703894	0.020361278	0.67109181	yes	down
ENSSSCG00000005303	PIGO	0.747965777	-0.418955834	0.045776676	0.72213448	yes	down
ENSSSCG00000005350	FBXO10	0.536400039	-0.898618753	0.004184472	0.475451096	yes	down
ENSSSCG00000005361	ALDH1B1	0.780678943	-0.357198739	0.047852739	0.72213448	yes	down
ENSSSCG00000005376	TBC1D2	0.724271972	-0.465396548	0.012821071	0.635895234	yes	down
ENSSSCG00000005380	COL15A1	0.817443975	-0.290808238	0.003002997	0.419992148	yes	down
ENSSSCG00000005453	TXN	1.361593201	0.445295738	0.005229217	0.513485708	yes	up
ENSSSCG00000005455	SVEP1	0.789488137	-0.341010506	0.034031544	0.704233345	yes	down
ENSSSCG00000005498	PAPPA	0.43036667	-1.216361742	0.009558124	0.603302839	yes	down
ENSSSCG00000005510	PHF19	0.780160884	-0.35815643	0.014117789	0.651736454	yes	down
ENSSSCG00000005524	DAB2IP	0.777016946	-0.363982032	0.037853653	0.709061263	yes	down
ENSSSCG00000005593	OLFML2A	1.261491078	0.335130003	0.003531692	0.447112352	yes	up
ENSSSCG00000005609	GARNL3	0.722335483	-0.469259054	0.029691005	0.697179556	yes	down
ENSSSCG00000005617	STXBP1	0.699951077	-0.514674007	0.00900882	0.591348223	yes	down
ENSSSCG00000005638	LCN2	2.305079789	1.204816689	0.029408737	0.696976005	yes	up
ENSSSCG00000005657	PKN3	0.718874508	-0.476188151	0.010298095	0.616343837	yes	down
ENSSSCG00000005680	PRRX2	0.559973391	-0.836569821	0.022939557	0.675846472	yes	down
ENSSSCG00000005743	VAV2	0.740210018	-0.433993433	0.03093343	0.703664106	yes	down
ENSSSCG00000005751	COL5A1	0.701269746	-0.511958605	0.01921589	0.67109181	yes	down
ENSSSCG00000005761	PPP1R26	0.621852148	-0.685356489	0.016353406	0.67109181	yes	down
ENSSSCG00000005832	MAMDC4	0.741319562	-0.431832512	0.024516147	0.67891626	yes	down
ENSSSCG00000005844	NRARP	0.52132849	-0.939735391	0.002149493	0.362946196	yes	down
ENSSSCG00000005855	EXD3	0.687713102	-0.540121263	0.049737385	0.728012358	yes	down

ENSSSCG00000005859	NDOR1	0.741080326	-0.432298169	0.048665789	0.72213448	yes	down
ENSSSCG00000005896	LRRC24	0.59287375	-0.754203173	0.016945454	0.67109181	yes	down
ENSSSCG00000005905	SLC39A4	0.759493134	-0.396891172	0.015157003	0.656815274	yes	down
ENSSSCG00000005911	OPLAH	0.612079578	-0.708208862	0.032985637	0.703664106	yes	down
ENSSSCG00000005918	DGAT1	0.722576562	-0.468777636	0.02577658	0.683048327	yes	down
ENSSSCG00000005924	MROH1	0.726086096	-0.461787468	0.038431995	0.71278147	yes	down
ENSSSCG00000005934	TRAPPC9	0.759838063	-0.396236112	0.037285538	0.707740306	yes	down
ENSSSCG00000005941	KHDRBS3	0.429692863	-1.218622279	0.044279852	0.72213448	yes	down
ENSSSCG00000005946	CCN4	0.744130317	-0.426372797	0.024700582	0.67891626	yes	down
ENSSSCG00000005997	COL14A1	0.801164249	-0.319830051	0.047362464	0.72213448	yes	down
ENSSSCG00000006141	CA3	0.519426509	-0.94500845	0.036930052	0.707740306	yes	down
ENSSSCG00000006164	WNT8B	0.349661438	-1.515969395	0.000755702	0.274823785	yes	down
ENSSSCG00000006166	NDUFB8	1.269345409	0.344084702	0.003358034	0.445536806	yes	up
ENSSSCG00000006245	SDR16C5	0.100081876	-3.320747357	0.024995403	0.679826869	yes	down
ENSSSCG00000006266	ST18	1.48685828	0.572267143	0.041548625	0.715680171	yes	up
ENSSSCG00000006274	PRKDC	0.828968317	-0.270611132	0.012418972	0.632336999	yes	down
ENSSSCG00000006298	XCL1	2.288986954	1.19470924	3.87E-05	0.070265172	yes	up
ENSSSCG00000006302	GPR161	0.614186403	-0.70325152	0.035000938	0.707740306	yes	down
ENSSSCG00000006390	CASQ1	1.934654364	0.952075844	0.049780508	0.728012358	yes	up
ENSSSCG00000006398	SLAMF8	1.702242911	0.767436925	0.034182478	0.704424911	yes	up
ENSSSCG00000006423	OR6N2	3.682743132	1.880780774	0.039944548	0.714830695	yes	up
ENSSSCG00000006472	CRABP2	0.416453651	-1.263772156	0.017523938	0.67109181	yes	down
ENSSSCG00000006489	TMEM79	0.629848343	-0.666923601	0.008783608	0.589351395	yes	down
ENSSSCG00000006502	ARHGEF2	0.800888659	-0.320326404	0.008387538	0.578565651	yes	down
ENSSSCG00000006525	MUC1	1.563431322	0.644715847	0.002124863	0.362946196	yes	up
ENSSSCG00000006588	S100A9	2.340040163	1.226533292	0.037207714	0.707740306	yes	up
ENSSSCG00000006626	PI4KB	0.811024317	-0.302182923	0.020887559	0.67109181	yes	down
ENSSSCG00000006651	ADAMTSL4	0.774672995	-0.368340647	0.04928037	0.724249616	yes	down
ENSSSCG00000006702	FMO5	1.355908342	0.439259657	0.002218894	0.364193212	yes	up
ENSSSCG00000006732	TRIM45	0.807261203	-0.308892538	0.020786203	0.67109181	yes	down
ENSSSCG00000006766	PHTF1	0.728556688	-0.456886865	0.014878697	0.6537276	yes	down
ENSSSCG00000006811	SLC6A17	0.369990099	-1.43444143	0.004065435	0.475451096	yes	down
ENSSSCG00000006828	ATXN7L2	0.636305863	-0.652207681	0.001776846	0.335087945	yes	down
ENSSSCG00000006923	GBP2	1.712573502	0.776165909	0.006518719	0.527035893	yes	up
ENSSSCG00000006926	GTF2B	1.21722683	0.283598039	0.005665717	0.523737709	yes	up
ENSSSCG00000006935	CLCA2	0.647362006	-0.6273554	0.032717938	0.703664106	yes	down
ENSSSCG00000006950	DNAI3	0.149289445	-2.743815929	0.017625806	0.67109181	yes	down
ENSSSCG00000006956	ZC3H3	0.694641437	-0.525659622	0.014417861	0.652664121	yes	down
ENSSSCG00000006981	MICU3	3.199802646	1.677982927	0.032754425	0.703664106	yes	up
ENSSSCG00000007133	ACSS1	0.761738713	-0.392631877	0.027116974	0.690849599	yes	down
ENSSSCG00000007147	HSPA12B	0.773940084	-0.369706212	0.013208165	0.635895234	yes	down

ENSSSCG00000007157	SLC4A11	0.516891311	-0.952067143	0.032282012	0.703664106	yes	down
ENSSSCG00000007286	ACSS2	0.705090426	-0.504119803	0.030714117	0.702410257	yes	down
ENSSSCG00000007324	SOGA1	0.788091854	-0.343564306	0.049917681	0.728012358	yes	down
ENSSSCG00000007382	PABPC1L	0.377951692	-1.403726247	0.029101037	0.696976005	yes	down
ENSSSCG00000007384	WFDC5	4.857030811	2.280074638	0.000831552	0.285607165	yes	up
ENSSSCG00000007433	PCIF1	0.804597827	-0.313660253	0.036358995	0.707740306	yes	down
ENSSSCG00000007436	MMP9	3.066820878	1.616743907	0.012916679	0.635895234	yes	up
ENSSSCG00000007437	SLC12A5	2.163512893	1.113375718	0.036316228	0.707740306	yes	up
ENSSSCG00000007466	SLC9A8	0.825354065	-0.276914948	0.034689682	0.707740306	yes	down
ENSSSCG00000007478	ATP9A	0.777626044	-0.362851556	0.038438624	0.71278147	yes	down
ENSSSCG00000007481	ZFP64	0.721259101	-0.471410478	0.044524397	0.72213448	yes	down
ENSSSCG00000007512	PPP4R1	0.758841198	-0.398130089	0.009274111	0.598675994	yes	down
ENSSSCG00000007530	PPP1R3D	0.596975284	-0.744256892	0.002789923	0.413957683	yes	down
ENSSSCG00000007531	FAM217B	0.664789262	-0.589031016	0.004306669	0.475451096	yes	down
ENSSSCG00000007543	DNAAF5	0.736137677	-0.441952482	0.023039754	0.675846472	yes	down
ENSSSCG00000007547	ADAP1	0.732882233	-0.448346705	0.031102826	0.703664106	yes	down
ENSSSCG00000007557	MICALL2	0.658812274	-0.602060661	0.029517242	0.697179556	yes	down
ENSSSCG00000007558	INTS1	0.77369015	-0.370172189	0.046292913	0.72213448	yes	down
ENSSSCG00000007574	SDK1	0.659862357	-0.599762975	0.04831216	0.72213448	yes	down
ENSSSCG00000007576	FOXK1	0.805486577	-0.312067548	0.038909871	0.714516217	yes	down
ENSSSCG00000007577	AP5Z1	0.755304835	-0.404869072	0.025847994	0.683249021	yes	down
ENSSSCG00000007642	AZGP1	0.588512297	-0.764855534	0.011195337	0.617982236	yes	down
ENSSSCG00000007648	GAL3ST4	67.32068458	6.072977942	0.045183644	0.72213448	yes	up
ENSSSCG00000007675	EPHB4	0.729391555	-0.455234599	0.007761106	0.565052652	yes	down
ENSSSCG00000007805	ATP2A1	0.72254107	-0.4688485	0.042457225	0.72177283	yes	down
ENSSSCG00000007806	RABEP2	0.735146283	-0.443896742	0.034706349	0.707740306	yes	down
ENSSSCG00000007940	C3H16orf96	0.383796583	-1.381586229	0.032381567	0.703664106	yes	down
ENSSSCG00000007951	CREBBP	0.817010337	-0.291573763	0.036406737	0.707740306	yes	down
ENSSSCG00000007958	ZNF174	0.772885814	-0.371672808	0.021164288	0.67109181	yes	down
ENSSSCG00000007967	ZNF213	0.68642284	-0.542830536	0.03293451	0.703664106	yes	down
ENSSSCG00000007968	RHBDF1	0.764662521	-0.387104932	0.009788074	0.603302839	yes	down
ENSSSCG00000007980	FAM234A	0.774856783	-0.367998413	0.043596975	0.72213448	yes	down
ENSSSCG00000007987	DECR2	0.730379547	-0.45328173	0.049813647	0.728012358	yes	down
ENSSSCG00000007992	PIGQ	0.717775941	-0.478394529	0.023626354	0.675846472	yes	down
ENSSSCG00000007993	CAPN15	0.719430547	-0.475072678	0.038672422	0.713688958	yes	down
ENSSSCG00000008022	TELO2	0.71261928	-0.488796579	0.025054138	0.679826869	yes	down
ENSSSCG00000008024	CLCN7	0.781096877	-0.356426603	0.034867008	0.707740306	yes	down
ENSSSCG00000008026	CRAMP1	0.755881153	-0.403768677	0.016631938	0.67109181	yes	down
ENSSSCG00000008032	mastin-like	2.074974485	1.053093596	0.040731093	0.715680171	yes	up
ENSSSCG00000008040	TSC2	0.748017385	-0.418856293	0.025003836	0.679826869	yes	down
ENSSSCG00000008041	PKD1	0.708817785	-0.496513292	0.019308835	0.67109181	yes	down

ENSSSCG00000008047	E4F1	0.733023539	-0.448068567	0.045406187	0.72213448	yes	down
ENSSSCG00000008051	ABCA3	0.710729033	-0.49262846	0.009703193	0.603302839	yes	down
ENSSSCG00000008073	OMD	0.423674927	-1.238970343	0.024655794	0.67891626	yes	down
ENSSSCG00000008115	CKLF-like	2.558048129	1.355043408	0.012499306	0.632336999	yes	up
ENSSSCG00000008117	ZNF2	1.228985951	0.297468424	0.033926903	0.703664106	yes	up
ENSSSCG00000008141	ST6GAL2	0.562124985	-0.831037154	0.004280653	0.475451096	yes	down
ENSSSCG00000008185	MITD1	1.443415609	0.529486761	0.025308736	0.679826869	yes	up
ENSSSCG00000008228	GNLY	2.646738844	1.40421585	9.39E-06	0.043529441	yes	up
ENSSSCG00000008397	EFEMP1	1.605473122	0.682998513	0.010504109	0.617982236	yes	up
ENSSSCG00000008400	CFAP36	1.321660647	0.402351794	0.010994578	0.617982236	yes	up
ENSSSCG00000008440	PIGF	1.240717142	0.311174248	0.039059668	0.714830695	yes	up
ENSSSCG00000008449	SLC3A1	0.530210352	-0.915363257	0.032521439	0.703664106	yes	down
ENSSSCG00000008486	GALM	0.830150377	-0.268555398	0.048288079	0.72213448	yes	down
ENSSSCG00000008554	ABHD1	1.474578325	0.560302456	0.020318263	0.67109181	yes	up
ENSSSCG00000008555	CGREF1	0.492165331	-1.02278506	0.005017531	0.513485708	yes	down
ENSSSCG00000008572	GAREM2	0.298882801	-1.742348212	0.001565838	0.335087945	yes	down
ENSSSCG00000008587	SF3B6	1.228342218	0.296712554	0.040330706	0.715680171	yes	up
ENSSSCG00000008595	APOB	0.212389971	-2.235212448	0.006570048	0.527035893	yes	down
ENSSSCG00000008617	CYRIA	1.47321061	0.558963692	0.03061286	0.700958898	yes	up
ENSSSCG00000008675	LETM1	0.717328804	-0.479293532	0.01101331	0.617982236	yes	down
ENSSSCG00000008697	HTT	0.784604809	-0.349961918	0.030887379	0.703664106	yes	down
ENSSSCG00000008722	SH3TC1	0.749017949	-0.416927803	0.00739868	0.553319813	yes	down
ENSSSCG00000008976	ART3	2.463744546	1.300852678	0.047195999	0.72213448	yes	up
ENSSSCG00000008981	STBD1	0.727529173	-0.458922996	0.020800433	0.67109181	yes	down
ENSSSCG00000008983	CCDC158	0.384847705	-1.377640451	0.011652688	0.623024075	yes	down
ENSSSCG00000008990	RP-L1	1.256833695	0.329793764	0.006236997	0.527035893	yes	up
ENSSSCG00000009004	SFRP2	0.502293515	-0.993397445	0.01269894	0.635895234	yes	down
ENSSSCG00000009009	MND1	1.484935108	0.570399887	0.018006004	0.67109181	yes	up
ENSSSCG00000009011	FHDC1	0.700861109	-0.512799525	0.016484535	0.67109181	yes	down
ENSSSCG00000009053	RNF150	0.805693134	-0.311697634	0.016131331	0.67109181	yes	down
ENSSSCG00000009101	PRDM5	1.520810796	0.604840678	0.042100914	0.719673409	yes	up
ENSSSCG00000009145	OSTC	1.271841038	0.346918365	0.043526276	0.72213448	yes	up
ENSSSCG00000009179	MTTP	0.103968274	-3.265784736	0.005339818	0.513485708	yes	down
ENSSSCG00000009316	PRHOXNB	0.563427843	-0.827697236	0.013723731	0.651736454	yes	down
ENSSSCG00000009330	ALOX5AP	1.450520772	0.536570956	0.008285861	0.578565651	yes	up
ENSSSCG00000009364	FREM2	0.65015542	-0.621143458	0.037096617	0.707740306	yes	down
ENSSSCG00000009379	NEK3	0.67524229	-0.566522833	0.001806696	0.335087945	yes	down
ENSSSCG00000009403	ITM2B	1.202483646	0.266017273	0.01182974	0.623024075	yes	up
ENSSSCG00000009407	ESD	1.23026913	0.29897395	0.025779578	0.683048327	yes	up
ENSSSCG00000009422	TSC22D1	1.289988862	0.36735861	0.017069175	0.67109181	yes	up
ENSSSCG00000009466	UCHL3	1.249500495	0.321351472	0.022906176	0.675846472	yes	up

ENSSSCG00000009486	SLITRK6	1.496161933	0.58126633	0.01752125	0.67109181	yes	up
ENSSSCG00000009527	FGF14	0.168648774	-2.56790626	0.000916023	0.308899506	yes	down
ENSSSCG00000009535	EFNB2	0.792379479	-0.335736577	0.028971697	0.696976005	yes	down
ENSSSCG00000009545	COL4A2	0.715104315	-0.483774386	0.021167235	0.67109181	yes	down
ENSSSCG00000009551	ARHGEF7	0.826934492	-0.274155048	0.036533972	0.707740306	yes	down
ENSSSCG00000009592	NFIL3	0.713671859	-0.486667209	0.017927542	0.67109181	yes	down
ENSSSCG00000009593	ROR2	0.756931336	-0.401765661	0.033101249	0.703664106	yes	down
ENSSSCG00000009610	NPM2	0.128265448	-2.962795501	0.020531548	0.67109181	yes	down
ENSSSCG00000009616	HR	0.739772669	-0.434846095	0.048816755	0.722589262	yes	down
ENSSSCG00000009649	NEFL	0.659308476	-0.600974467	0.026429877	0.685939631	yes	down
ENSSSCG00000009668	CLU	0.581019423	-0.783341702	0.000515742	0.239136655	yes	down
ENSSSCG00000009672	SCARA5	0.644616876	-0.633486137	0.00932859	0.598675994	yes	down
ENSSSCG00000009761	NCOR2	0.758619272	-0.398552073	0.024122563	0.67597251	yes	down
ENSSSCG00000009784	ABCB9	0.691099219	-0.533035245	0.001038249	0.310915441	yes	down
ENSSSCG00000009823	VPS29	1.201693599	0.265069092	0.035823165	0.707740306	yes	up
ENSSSCG00000009832	PHETA1	0.726210693	-0.461539922	0.046507019	0.72213448	yes	down
ENSSSCG00000009836	ACAD10	0.583900204	-0.776206281	0.000160084	0.133112815	yes	down
ENSSSCG00000009842	CCDC60	0.06025313	-4.052819998	0.025137993	0.679826869	yes	down
ENSSSCG00000009856	NOS1	0.454593383	-1.137351411	0.024844725	0.67891626	yes	down
ENSSSCG00000009919	HNF1A	0.766832644	-0.383016342	0.037179972	0.707740306	yes	down
ENSSSCG00000009931	MVK	0.709610285	-0.494901176	0.018624237	0.67109181	yes	down
ENSSSCG00000009940	ALKBH2	1.334363325	0.416151542	0.013014004	0.635895234	yes	up
ENSSSCG00000009967	PITPNB	1.201979545	0.265412345	0.023167294	0.675846472	yes	up
ENSSSCG00000010009	GAL3ST1	0.62628483	-0.675109161	0.046356249	0.72213448	yes	down
ENSSSCG00000010017	SMTN	0.791172806	-0.337935257	0.022324702	0.673830277	yes	down
ENSSSCG00000010097	LZTR1	0.754056208	-0.407256027	0.025449946	0.679826869	yes	down
ENSSSCG00000010101	P2RX6	0.471582369	-1.084418314	0.027974885	0.693082926	yes	down
ENSSSCG00000010107	MED15	0.788580593	-0.342669888	0.036301739	0.707740306	yes	down
ENSSSCG00000010133	ARVCF	0.72987424	-0.454280192	0.015305859	0.657271396	yes	down
ENSSSCG00000010184	AGT	0.661277886	-0.596671439	0.023810446	0.67597251	yes	down
ENSSSCG00000010196	ZNF25	1.382486211	0.467265091	0.031455649	0.703664106	yes	up
ENSSSCG00000010258	AIFM2	0.724036223	-0.465866219	0.028242127	0.695002653	yes	down
ENSSSCG00000010291	PLA2G12B	0.366845792	-1.446754359	0.012317872	0.631103805	yes	down
ENSSSCG00000010302	USP54	0.797621314	-0.326224133	0.013234246	0.635895234	yes	down
ENSSSCG00000010308	CHCHD1	1.206142583	0.270400465	0.040311283	0.715680171	yes	up
ENSSSCG00000010334	SFTPD	1.770327832	0.824016545	0.045179414	0.72213448	yes	up
ENSSSCG00000010446	STAMBPL1	1.202795935	0.266391898	0.029659665	0.697179556	yes	up
ENSSSCG00000010478	FFAR4	0.648461419	-0.624907352	0.000503539	0.239136655	yes	down
ENSSSCG00000010481	LGI1	1.696471944	0.762537572	0.022077226	0.673830277	yes	up
ENSSSCG00000010496	TCTN3	1.275384175	0.350931885	0.040094199	0.714830695	yes	up
ENSSSCG00000010529	SFRP5	1.424236993	0.510189231	0.005302423	0.513485708	yes	up

ENSSSCG00000010533	PYROXD2	0.606517336	-0.721379213	0.005320096	0.513485708	yes	down
ENSSSCG00000010566	FBXW4	0.783898849	-0.351260588	0.037020029	0.707740306	yes	down
ENSSSCG00000010570	KCNIP2	0.475772721	-1.071655541	0.044365724	0.72213448	yes	down
ENSSSCG00000010604	SH3PXD2A	0.787814112	-0.344072835	0.028596138	0.69504785	yes	down
ENSSSCG00000010629	ADRA2A	0.40554452	-1.302067797	0.027893666	0.693082926	yes	down
ENSSSCG00000010640	NRAP	0.749269177	-0.416443992	0.023421881	0.675846472	yes	down
ENSSSCG00000010732	FAM53B	0.44358417	-1.172720213	0.00074262	0.274823785	yes	down
ENSSSCG00000010832	HHIPL2	0.460182796	-1.119721046	0.000512857	0.239136655	yes	down
ENSSSCG00000010861	COQ8A	0.557594894	-0.842710744	0.000536202	0.241964073	yes	down
ENSSSCG00000010925	PPP1R12B	0.77449635	-0.368669655	0.02051067	0.67109181	yes	down
ENSSSCG00000010948	CTSL	0.5707532	-0.809061052	0.016688788	0.67109181	yes	down
ENSSSCG00000010957	AGTPBP1	1.294553778	0.372454899	0.002489542	0.379220559	yes	up
ENSSSCG00000010992	AQP7	0.238676847	-2.066869469	0.028631587	0.69504785	yes	down
ENSSSCG00000011056	FRMD4A	0.826840878	-0.27431838	0.047611865	0.72213448	yes	down
ENSSSCG00000011075	KIAA1217	0.83130964	-0.266542154	0.044098285	0.72213448	yes	down
ENSSSCG00000011076	OTUD1	0.548007308	-0.867732961	0.042818164	0.72213448	yes	down
ENSSSCG00000011119	ECHDC3	0.647479385	-0.627093835	0.013913944	0.651736454	yes	down
ENSSSCG00000011234	CMTM7	1.418221993	0.504083374	0.006656066	0.527035893	yes	up
ENSSSCG00000011241	DCLK3	0.135127618	-2.887605529	0.031201788	0.703664106	yes	down
ENSSSCG00000011246	VILL	0.799261795	-0.323259966	0.031587871	0.703664106	yes	down
ENSSSCG00000011258	EXOG	1.91055	0.933988014	0.030314868	0.700958898	yes	up
ENSSSCG00000011270	EIF1B	1.222605703	0.289959202	0.014779691	0.652664121	yes	up
ENSSSCG00000011316	CCR9	1.595870993	0.674344032	0.037924666	0.709061263	yes	up
ENSSSCG00000011346	PLXNB1	0.77957705	-0.359236475	0.027808992	0.693082926	yes	down
ENSSSCG00000011358	TREX1	1.645158875	0.718226914	0.030468716	0.700958898	yes	up
ENSSSCG00000011423	GRM2	0.616299932	-0.698295464	0.024133292	0.67597251	yes	down
ENSSSCG00000011441	TNNC1	3.942149388	1.978982448	0.013214458	0.635895234	yes	up
ENSSSCG00000011471	FLNB	0.781215852	-0.356206871	6.90E-05	0.083559738	yes	down
ENSSSCG00000011482	CFAP20DC	0.796006625	-0.329147657	0.039970068	0.714830695	yes	down
ENSSSCG00000011494	PSMD6	1.213668807	0.279374784	0.025360553	0.679826869	yes	up
ENSSSCG00000011643	AMOTL2	0.799160758	-0.323442353	0.036201251	0.707740306	yes	down
ENSSSCG00000011673	TSPAN6	0.653958672	-0.612728631	0.001249679	0.313270149	yes	down
ENSSSCG00000011700	CP	2.701651366	1.433841515	0.048137323	0.72213448	yes	up
ENSSSCG00000011714	MED12L	1.525500796	0.609282933	0.04065934	0.715680171	yes	up
ENSSSCG00000011739	PDCD10	1.251450643	0.323601393	0.013873405	0.651736454	yes	up
ENSSSCG00000011826	TMEM44	0.757501207	-0.400679908	0.006562148	0.527035893	yes	down
ENSSSCG00000011860	SLC12A8	0.487795784	-1.035650806	7.13E-05	0.083559738	yes	down
ENSSSCG00000011862	MUC13	0.628611909	-0.669758491	0.010472083	0.617982236	yes	down
ENSSSCG00000011946	ALCAM	1.241131743	0.311656262	0.044431208	0.72213448	yes	up
ENSSSCG00000011956	TRMT10C	1.246702102	0.318116777	0.040911836	0.715680171	yes	up
ENSSSCG00000012066	KCNJ15	3.649780958	1.867809883	0.029691945	0.697179556	yes	up

ENSSSCG00000012119	TMSB4X	1.261694591	0.33536273	0.023065662	0.675846472	yes	up
ENSSSCG00000012161	MAP7D2	2.31254935	1.209484153	0.006493736	0.527035893	yes	up
ENSSSCG00000012182	PCYT1B	0.371944369	-1.42684124	0.028158018	0.695002653	yes	down
ENSSSCG00000012251	USP9X	1.274318749	0.349726188	0.013782679	0.651736454	yes	up
ENSSSCG00000012262	KDM6A	1.403385534	0.488911397	0.01976651	0.67109181	yes	up
ENSSSCG00000012273	USP11	0.78043467	-0.357650226	0.044377219	0.72213448	yes	down
ENSSSCG00000012295	MAGIX	0.674002182	-0.569174833	0.037387713	0.707740306	yes	down
ENSSSCG00000012315	SYNJ2	0.801543249	-0.319147729	0.017002219	0.67109181	yes	down
ENSSSCG00000012352	KLF8	0.541302292	-0.885493599	0.025670123	0.682097083	yes	down
ENSSSCG00000012363	HEPH	0.744411511	-0.425827731	0.00043235	0.229108458	yes	down
ENSSSCG00000012448	ITM2A	1.515447984	0.599744334	0.011203117	0.617982236	yes	up
ENSSSCG00000012504	NAP1L3	1.276107284	0.351749624	0.048932739	0.722841414	yes	up
ENSSSCG00000012551	RNF128	1.276196543	0.351850531	0.001214897	0.312954017	yes	up
ENSSSCG00000012618	SOWAHD	0.70221364	-0.510018075	0.04155861	0.715680171	yes	down
ENSSSCG00000012651	XPNPEP2	0.635373577	-0.654323	0.020805862	0.67109181	yes	down
ENSSSCG00000012766	BGN	0.663819778	-0.591136482	0.032708226	0.703664106	yes	down
ENSSSCG00000012767	HAUS7	0.78905769	-0.341797312	0.004882258	0.513485708	yes	down
ENSSSCG00000012793	HCFC1	0.756794473	-0.402026542	0.039499796	0.714830695	yes	down
ENSSSCG00000012795	IRAK1	0.742105503	-0.43030379	0.034070298	0.704233345	yes	down
ENSSSCG00000012838	TSPAN4	0.698602542	-0.517456202	0.014565109	0.652664121	yes	down
ENSSSCG00000012843	PIDD1	0.707295001	-0.49961603	0.007529269	0.558581442	yes	down
ENSSSCG00000012862	OSBPL5	0.748203033	-0.418498282	0.028684289	0.69504785	yes	down
ENSSSCG00000012878	IGHMBP2	0.756354168	-0.402866151	0.027797093	0.693082926	yes	down
ENSSSCG00000012885	LRP5	0.732300557	-0.449492202	0.0489228	0.722841414	yes	down
ENSSSCG00000012903	PITPNM1	0.756352125	-0.402870048	0.020663259	0.67109181	yes	down
ENSSSCG00000012975	SNX32	0.406356607	-1.299181742	0.019839423	0.67109181	yes	down
ENSSSCG00000012983	PCNX3	0.753540497	-0.408243047	0.047496099	0.72213448	yes	down
ENSSSCG00000013039	NUDT22	1.297228488	0.375432611	0.02419889	0.67597251	yes	up
ENSSSCG00000013060	SCGB1A1	4.788982773	2.259719246	0.031817741	0.703664106	yes	up
ENSSSCG00000013079	DAGLA	0.703882474	-0.506593531	0.029994811	0.700647043	yes	down
ENSSSCG00000013117	CBLIF	0.257505768	-1.957323344	0.03122602	0.703664106	yes	down
ENSSSCG00000013248	LRP4	0.791080696	-0.338103227	0.038581342	0.71278147	yes	down
ENSSSCG00000013286	LRRC4C	1.904601101	0.929488872	0.008587174	0.587698606	yes	up
ENSSSCG00000013369	SAA2-like	11.57938711	3.533486989	0.026037526	1	yes	up
ENSSSCG00000013382	PLEKHA7	0.781352288	-0.355954932	0.001633151	0.335087945	yes	down
ENSSSCG00000013433	ADAMTSL5	0.660114051	-0.599212788	0.019494209	0.67109181	yes	down
ENSSSCG00000013448	MKNK2	0.668562608	-0.580865426	0.016083855	0.67109181	yes	down
ENSSSCG00000013457	DOT1L	0.789140017	-0.341646795	0.003329207	0.445536806	yes	down
ENSSSCG00000013497	ANKRD24	0.660425308	-0.598532689	0.023846071	0.67597251	yes	down
ENSSSCG00000013501	CREB3L3	0.618650065	-0.692804505	0.008237681	0.578565651	yes	down
ENSSSCG00000013653	ICAM5	0.2166775	-2.206378743	0.032803871	0.703664106	yes	down

ENSSSCG00000013754	CACNA1A	0.624829064	-0.678466532	0.019238518	0.67109181	yes	down
ENSSSCG00000013829	SYDE1	0.760041227	-0.395850419	0.017106654	0.67109181	yes	down
ENSSSCG00000013836	WIZ	0.71382719	-0.48635324	0.004953974	0.513485708	yes	down
ENSSSCG00000013867	SIN3B	0.776925173	-0.364152438	0.045376659	0.72213448	yes	down
ENSSSCG00000013915	CRTC1	0.789596986	-0.340811613	0.034648378	0.707740306	yes	down
ENSSSCG00000013931	GMIP	0.744176512	-0.426283239	0.016919685	0.67109181	yes	down
ENSSSCG00000013932	LPAR2	0.68276595	-0.550536983	0.032173158	0.703664106	yes	down
ENSSSCG00000014038	FAM193B	0.794256323	-0.332323425	0.037476574	0.707740306	yes	down
ENSSSCG00000014048	HK3	1.682355633	0.750482709	0.011604222	0.623024075	yes	up
ENSSSCG00000014149	MEF2C	1.250218232	0.322179947	0.028507873	0.695002653	yes	up
ENSSSCG00000014182	SLCO4C1	2.255084954	1.173181784	0.006203051	0.527035893	yes	up
ENSSSCG00000014211	YTHDC2	1.272308713	0.347448769	0.018954156	0.67109181	yes	up
ENSSSCG00000014283	KIF3A	1.240494498	0.310915336	0.027866794	0.693082926	yes	up
ENSSSCG00000014303	JADE2	0.648455965	-0.624919487	0.005647377	0.523737709	yes	down
ENSSSCG00000014399	ARHGAP26	0.805425203	-0.312177478	0.005745374	0.523737709	yes	down
ENSSSCG00000014410	SH3RF2	0.726657145	-0.460653269	0.009670055	0.603302839	yes	down
ENSSSCG00000014437	PPARGC1B	0.744517082	-0.425623145	0.03745815	0.707740306	yes	down
ENSSSCG00000014440	HMGXB3	0.746779757	-0.421245274	0.039276573	0.714830695	yes	down
ENSSSCG00000014561	NLRP6	0.465091843	-1.104412457	0.013901971	0.651736454	yes	down
ENSSSCG00000014567	TRIM66	0.708199114	-0.497773056	0.018496161	0.67109181	yes	down
ENSSSCG00000014570	NRIP3	0.449543513	-1.153467328	0.039597349	0.714830695	yes	down
ENSSSCG00000014632	FHIP1B	0.762881242	-0.390469605	0.020864982	0.67109181	yes	down
ENSSSCG00000014811	INPPL1	0.774471776	-0.368715431	0.03363754	0.703664106	yes	down
ENSSSCG00000014827	PLEKHB1	0.493882439	-1.017760423	0.031743708	0.703664106	yes	down
ENSSSCG00000014834	UCP3	0.541198428	-0.885770447	0.013341283	0.63938186	yes	down
ENSSSCG00000014850	SPCS2	1.224386704	0.292059284	0.028778663	0.695903344	yes	up
ENSSSCG00000014874	B3GNT6	0.510136944	-0.971043511	0.005134368	0.513485708	yes	down
ENSSSCG00000014894	TENM4	0.511762155	-0.966454633	0.007018172	0.537876203	yes	down
ENSSSCG00000014933	NAALAD2	0.075848539	-3.72073479	0.034160646	1	yes	down
ENSSSCG00000014960	AMOTL1	0.733435427	-0.447258141	0.019926963	0.67109181	yes	down
ENSSSCG00000015098	CXCR5	0.613623855	-0.704573525	0.030053158	0.700714589	yes	down
ENSSSCG00000015111	C2CD2L	0.701957297	-0.510544826	0.017046733	0.67109181	yes	down
ENSSSCG00000015125	NECTIN1	0.729688905	-0.454646578	0.042182199	0.720398934	yes	down
ENSSSCG00000015138	JHY	2.145411505	1.101254394	0.017679255	0.67109181	yes	up
ENSSSCG00000015268	FMO1	0.668372692	-0.581275306	0.029533547	0.697179556	yes	down
ENSSSCG00000015277	SOX13	0.768928423	-0.379078787	0.034526518	0.707740306	yes	down
ENSSSCG00000015281	PLEKHA6	0.761364488	-0.393340814	0.024103951	0.67597251	yes	down
ENSSSCG00000015290	CDK18	0.696805472	-0.52117214	0.044256918	0.72213448	yes	down
ENSSSCG00000015293	CD46	1.586491884	0.665840141	0.036553216	0.707740306	yes	up
ENSSSCG00000015380	CDCA7L	1.204296303	0.268190394	0.035612156	0.707740306	yes	up
ENSSSCG00000015522	ANGPTL1	2.818075399	1.494710212	0.008810848	0.589351395	yes	up

ENSSSCG00000015538	KIAA1614	0.531243296	-0.912555365	0.000179426	0.133112815	yes	down
ENSSSCG00000015588	ANGEL2	1.217601127	0.284041599	0.01436171	0.652664121	yes	up
ENSSSCG00000015590	FLVCR1	1.415784583	0.50160177	0.000146695	0.133112815	yes	up
ENSSSCG00000015592	TATDN3	1.924540276	0.944513863	0.000132761	0.133112815	yes	up
ENSSSCG00000015618	LAMB3	0.666428721	-0.585477517	0.011002067	0.617982236	yes	down
ENSSSCG00000015632	COBL	0.695879486	-0.523090616	0.037275505	0.707740306	yes	down
ENSSSCG00000015673	KYNU	1.337547243	0.41958985	0.033585847	0.703664106	yes	up
ENSSSCG00000015716	MARCO	0.456015405	-1.132845532	0.023177476	0.675846472	yes	down
ENSSSCG00000015769	SPCS3	1.267264969	0.341718206	0.018579891	0.67109181	yes	up
ENSSSCG00000015796	PDLIM3	1.545664318	0.628227033	0.002861516	0.417894019	yes	up
ENSSSCG00000015828	ZNF703	0.736862435	-0.440532787	0.029387696	0.696976005	yes	down
ENSSSCG00000015840	WRN	1.545574396	0.628143099	0.003049988	0.422150242	yes	up
ENSSSCG00000015850	DUSP4	0.812455816	-0.299638738	0.01920465	0.67109181	yes	down
ENSSSCG00000015864	MYO7B	0.67302722	-0.57126324	0.008865533	0.589351395	yes	down
ENSSSCG00000015914	SCN7A	1.547888676	0.630301717	0.036158998	0.707740306	yes	up
ENSSSCG00000015923	NOSTRIN	0.822924566	-0.281167903	0.048620238	0.72213448	yes	down
ENSSSCG00000015935	CFAP210	2.160754895	1.11153543	0.02333234	0.675846472	yes	up
ENSSSCG00000015985	HOXD3	0.720642231	-0.472644896	0.009313374	0.598675994	yes	down
ENSSSCG00000015988	HNRNPA1_3	1.224663541	0.292385443	0.040649299	0.715680171	yes	up
ENSSSCG00000016035	COL5A2	0.772685149	-0.372047425	0.014944762	0.6537276	yes	down
ENSSSCG00000016059	STAT4	1.551212876	0.633396684	0.016604498	0.67109181	yes	up
ENSSSCG00000016166	SPAG16	1.960774237	0.971423433	0.007357943	0.552501087	yes	up
ENSSSCG00000016174	FN1	0.746793546	-0.421218636	0.017104169	0.67109181	yes	down
ENSSSCG00000016184	CXCR2	1.889524352	0.918023112	0.032345547	0.703664106	yes	up
ENSSSCG00000016216	TUBA4A	1.309392315	0.388897417	0.049864941	0.728012358	yes	up
ENSSSCG00000016256	SPHKAP	0.424574227	-1.235911296	0.017922662	0.67109181	yes	down
ENSSSCG00000016295	NGEF	0.710369189	-0.493359086	0.005784555	0.523737709	yes	down
ENSSSCG00000016317	AGAP1	0.780439646	-0.357641027	0.04423407	0.72213448	yes	down
ENSSSCG00000016328	RAB17	0.725003143	-0.463940846	0.044868415	0.72213448	yes	down
ENSSSCG00000016340	ASB1	0.789656196	-0.340703433	0.048314254	0.72213448	yes	down
ENSSSCG00000016346	MAD2L1BP	1.243919259	0.314892845	0.020047169	0.67109181	yes	up
ENSSSCG00000016367	RNPEPL1	0.675025616	-0.566985844	0.031366492	0.703664106	yes	down
ENSSSCG00000016368	FARP2	0.644972732	-0.632689928	0.003543728	0.447112352	yes	down
ENSSSCG00000016437	WDR86	0.601992908	-0.732181604	0.002106555	0.362946196	yes	down
ENSSSCG00000016492	AGK	0.815402094	-0.294416434	0.042399549	0.721453604	yes	down
ENSSSCG00000016522	PTN	0.626410744	-0.674819138	2.01E-05	0.055287609	yes	down
ENSSSCG00000016642	GPR85	0.340157935	-1.555723352	0.002210592	0.364193212	yes	down
ENSSSCG00000016665	BMPER	0.751987384	-0.411219636	0.018971935	0.67109181	yes	down
ENSSSCG00000016726	ADCY1	0.51272072	-0.963754893	0.009023106	0.591348223	yes	down
ENSSSCG00000016754	AEBP1	0.754886485	-0.405668377	0.00831083	0.578565651	yes	down
ENSSSCG00000016794	MYO10	0.800262928	-0.321454016	0.033217978	0.703664106	yes	down

ENSSSCG00000016827	BRIX1	1.247607919	0.319164615	0.027081571	0.690849599	yes	up
ENSSSCG00000016832	IL7R	1.403130508	0.488649203	0.004182977	0.475451096	yes	up
ENSSSCG00000016883	ISL1	0.476297859	-1.07006403	0.033099455	0.703664106	yes	down
ENSSSCG00000016893	NDUFS4	1.22408719	0.291706323	0.02063021	0.67109181	yes	up
ENSSSCG00000016902	GZMA	1.868644611	0.901992215	0.019373664	0.67109181	yes	up
ENSSSCG00000017037	FABP6	0.189113543	-2.402675414	1.61E-05	0.055287609	yes	down
ENSSSCG00000017042	UBLCP1	1.207642566	0.272193513	0.036687749	0.707740306	yes	up
ENSSSCG00000017044	IL12B	3.956031236	1.984053817	0.033017446	0.703664106	yes	up
ENSSSCG00000017121	SLC6A3	0.468841015	-1.092829309	0.03350361	0.703664106	yes	down
ENSSSCG00000017128	HEXD	0.575060992	-0.798213116	0.009093971	0.591810091	yes	down
ENSSSCG00000017134	FN3KRP	0.788996459	-0.34190927	0.033159768	0.703664106	yes	down
ENSSSCG00000017136	TBCD	0.73409099	-0.4459692	0.027720063	0.693082926	yes	down
ENSSSCG00000017139	RPTOR	0.706533448	-0.501170235	0.021846106	0.673830277	yes	down
ENSSSCG00000017156	GAA	0.723908261	-0.466121214	0.03852601	0.71278147	yes	down
ENSSSCG00000017168	SEPTIN9	0.76917498	-0.378616261	0.012448773	0.632336999	yes	down
ENSSSCG00000017172	ST6GALNAC1	0.541752179	-0.884295043	0.019593159	0.67109181	yes	down
ENSSSCG00000017195	TRIM65	0.771156779	-0.3749039	0.013510073	0.64580238	yes	down
ENSSSCG00000017197	FBF1	0.7245081	-0.464926276	0.013587863	0.647851163	yes	down
ENSSSCG00000017201	UNK	0.785469191	-0.348373406	0.024178758	0.67597251	yes	down
ENSSSCG00000017204	ITGB4	0.641354467	-0.640806162	0.000263034	0.174231894	yes	down
ENSSSCG00000017237	CD300LB	2.919081329	1.545514407	0.012743118	0.635895234	yes	up
ENSSSCG00000017238	TTYH2	0.824975896	-0.277576127	0.04389221	0.72213448	yes	down
ENSSSCG00000017245	CDC42EP4	0.766507792	-0.383627637	0.017273753	0.67109181	yes	down
ENSSSCG00000017258	FAM20A	0.565934251	-0.821293642	0.001382311	0.324528025	yes	down
ENSSSCG00000017261	ARSG	1.833275107	0.874423297	0.023646977	0.675846472	yes	up
ENSSSCG00000017273	PSMD12	1.288362259	0.365538305	0.044097442	0.72213448	yes	up
ENSSSCG00000017297	CYB561	0.74816497	-0.418571677	0.012186165	0.629573257	yes	down
ENSSSCG00000017300	MRC2	0.727330625	-0.459316771	0.001276383	0.313270149	yes	down
ENSSSCG00000017518	OSBPL7	0.740131782	-0.434145927	0.039714435	0.714830695	yes	down
ENSSSCG00000017534	HOXB3	0.479576201	-1.060168029	0.003363086	0.445536806	yes	down
ENSSSCG00000017539	HOXB6	0.719495753	-0.474941923	0.01166706	0.623024075	yes	down
ENSSSCG00000017548	NGFR	0.618209961	-0.693831196	0.046629859	0.72213448	yes	down
ENSSSCG00000017565	SPATA20	0.738605037	-0.437124994	0.041754537	0.717056849	yes	down
ENSSSCG00000017567	RSAD1	0.600241152	-0.736385862	7.21E-05	0.083559738	yes	down
ENSSSCG00000017578	ITGA3	0.702835694	-0.508740634	0.001425495	0.326403208	yes	down
ENSSSCG00000017585	SAMD14	0.634052595	-0.657325578	0.048588716	0.72213448	yes	down
ENSSSCG00000017645	TEX14	0.396350039	-1.335152977	0.014761603	0.652664121	yes	down
ENSSSCG00000017696	C17orf78	2.274136014	1.185318543	0.014059136	0.651736454	yes	up
ENSSSCG00000017700	CCL3L1	2.209634615	1.143807825	0.033291855	0.703664106	yes	up
ENSSSCG00000017705	CCL5	1.594569293	0.673166792	0.049620533	0.726944728	yes	up
ENSSSCG00000017753	KSR1	0.759931804	-0.396058137	0.001728411	0.335087945	yes	down

ENSSSCG00000017781	PIPOX	0.157543852	-2.666174639	0.001762033	0.335087945	yes	down
ENSSSCG00000017783	MYO18A	0.81303604	-0.29860879	0.033525182	0.703664106	yes	down
ENSSSCG00000017829	SGSM2	0.80535791	-0.312298019	0.044544817	0.72213448	yes	down
ENSSSCG00000017879	SPNS2	0.57975466	-0.786485583	0.010301752	0.616343837	yes	down
ENSSSCG00000017902	CAMTA2	0.734970705	-0.444241348	0.017941127	0.67109181	yes	down
ENSSSCG00000017929	RNASEK	1.298323841	0.37665028	0.001785511	0.335087945	yes	up
ENSSSCG00000017932	ASGR2	0.541024853	-0.886233225	0.00534333	0.513485708	yes	down
ENSSSCG00000017968	CNTROB	0.72742565	-0.459128298	0.014147432	0.651736454	yes	down
ENSSSCG00000017995	USP43	0.787355476	-0.344912963	0.037996326	0.709685665	yes	down
ENSSSCG00000018015	DNAH9	1.808118469	0.854489207	0.026670347	0.69011278	yes	up
ENSSSCG00000018058	ARHGAP23	0.74996883	-0.415097459	0.035220988	0.707740306	yes	down
ENSSSCG00000020750	IGKV2-30	94.53997546	6.562852585	0.002396233	0.379220559	yes	up
ENSSSCG00000020803	BRPF3	0.747494338	-0.419865443	0.009723027	0.603302839	yes	down
ENSSSCG00000020813	FAM20C	0.594227932	-0.750911673	0.003907377	0.474147655	yes	down
ENSSSCG00000020858	KIF13A	0.811836542	-0.300738815	0.032032608	0.703664106	yes	down
ENSSSCG00000021007	MRPL19	1.212831206	0.278378779	0.031552135	0.703664106	yes	up
ENSSSCG00000021158	MCF2L	0.647558487	-0.626917593	0.003465533	0.447112352	yes	down
ENSSSCG00000021322	ZFX Isoform X1	1.454033292	0.540060302	0.01928582	0.67109181	yes	up
ENSSSCG00000021427	DCTN4	0.806583022	-0.310105057	0.038074298	0.709706918	yes	down
ENSSSCG00000021467	EME2	0.708496713	-0.497166936	0.013162805	0.635895234	yes	down
ENSSSCG00000021475	SLC4A2	0.745205521	-0.424289732	0.038089041	0.709706918	yes	down
ENSSSCG00000021514	OCLN	1.239222577	0.309435333	0.048260303	0.72213448	yes	up
ENSSSCG00000021598	EVA1C	1.373756519	0.458126328	0.023090967	0.675846472	yes	up
ENSSSCG00000021624	Ladinin 1	0.693104608	-0.528854986	0.019874913	0.67109181	yes	down
ENSSSCG00000021646	KLF9	1.274182953	0.349572442	0.044352571	0.72213448	yes	up
ENSSSCG00000021702	XRRA1	0.611941963	-0.708533261	0.045197141	0.72213448	yes	down
ENSSSCG00000021825	RPS21	1.259982071	0.333403205	0.000785973	0.278545508	yes	up
ENSSSCG00000021997	ALS2CL	0.669891312	-0.578001054	0.009800207	0.603302839	yes	down
ENSSSCG00000022011	NMI	1.276426122	0.352110038	0.029824241	0.697541228	yes	up
ENSSSCG00000022031	CHST2	0.700140889	-0.514282832	0.018338837	0.67109181	yes	down
ENSSSCG00000022151	B3GALT6	0.678133653	-0.560358454	0.010794215	0.617982236	yes	down
ENSSSCG00000022162	RAB11FIP5	0.567523639	-0.817247608	0.001288418	0.313270149	yes	down
ENSSSCG00000022190	UBE2V2	1.214713571	0.280616167	0.046892405	0.72213448	yes	up
ENSSSCG00000022195	ZKSCAN7	1.378978855	0.463600335	0.030813614	0.703664106	yes	up
ENSSSCG00000022302	BICRA	0.735830249	-0.44255511	0.02770486	0.693082926	yes	down
ENSSSCG00000022310	VPS9D1	0.688951834	-0.53752497	0.029699069	0.697179556	yes	down
ENSSSCG00000022333	C16orf87	1.572625081	0.653174768	0.023685782	0.675846472	yes	up
ENSSSCG00000022380	SH2D3A	0.781206234	-0.356224633	0.016536067	0.67109181	yes	down
ENSSSCG00000022387	TBPL1	1.265843585	0.340099148	0.000565775	0.241964073	yes	up
ENSSSCG00000022395	SLC25A35	0.666716382	-0.584854919	0.010258337	0.616343837	yes	down

ENSSSCG00000022417	HNF1B	0.741450282	-0.431578139	0.030211893	0.700714589	yes	down
ENSSSCG00000022423	KRAB	0.566838818	-0.818989536	0.036956518	0.707740306	yes	down
ENSSSCG00000022490	GPR83	5.042049274	2.334010217	0.028379726	0.695002653	yes	up
ENSSSCG00000022550	DGKG	0.60749067	-0.719065843	0.01136205	0.617982236	yes	down
ENSSSCG00000022553	TNRC18	0.724078287	-0.465782406	0.023413218	0.675846472	yes	down
ENSSSCG00000022614	DQX1	0.562795828	-0.82931646	0.007901307	0.571776715	yes	down
ENSSSCG00000022722	CARMIL2	0.737157244	-0.439955699	0.045038805	0.72213448	yes	down
ENSSSCG00000022741	PDGFRB	0.7618269	-0.392464865	0.009705194	0.603302839	yes	down
ENSSSCG00000022968	ARFGEF3	0.63915396	-0.645764604	0.006555448	0.527035893	yes	down
ENSSSCG00000023001	CCDC149	0.722100861	-0.469727732	0.02625634	0.684917502	yes	down
ENSSSCG00000023045	BAHCC1	0.659041043	-0.601559781	0.001317468	0.313270149	yes	down
ENSSSCG00000023133	OSBPL6	0.622245229	-0.68444483	0.014984263	0.653913233	yes	down
ENSSSCG00000023228	NUMBL	0.725769531	-0.462416603	0.014196448	0.651736454	yes	down
ENSSSCG00000023235	MAN1C1	0.753882129	-0.407589122	0.001065149	0.310915441	yes	down
ENSSSCG00000023322	COL9A	0.430502324	-1.21590707	0.021017383	0.67109181	yes	down
ENSSSCG00000023329	APC2	0.581863914	-0.781246319	0.006020028	0.526667284	yes	down
ENSSSCG00000023371	EHD2	0.804413148	-0.313991433	0.001959555	0.352853088	yes	down
ENSSSCG00000023400	ZNF598	0.723209603	-0.467514261	0.041533791	0.715680171	yes	down
ENSSSCG00000023483	PLXNA3	0.688512863	-0.538444488	0.016488472	0.67109181	yes	down
ENSSSCG00000023520	PCSK5	0.708904078	-0.496337666	0.017374004	0.67109181	yes	down
ENSSSCG00000023611	TNXB	0.688109532	-0.539289866	0.023658506	0.675846472	yes	down
ENSSSCG00000023681	PWWP2B	0.720538946	-0.472851683	0.041075045	0.715680171	yes	down
ENSSSCG00000023852	CCL17	6.963572858	2.799827712	0.012944003	0.635895234	yes	up
ENSSSCG00000023907	AFAP1	0.679276533	-0.55792908	0.002919423	0.41873231	yes	down
ENSSSCG00000023909	THPO	0.527059513	-0.923962221	0.036900815	0.707740306	yes	down
ENSSSCG00000023936	MYRFL	0.108252722	-3.207524796	0.018125344	0.67109181	yes	down
ENSSSCG00000023971	H3-3A	1.225238757	0.293062908	0.000990969	0.310915441	yes	up
ENSSSCG00000024064	RNF6	0.804355347	-0.3140951	0.047051254	0.72213448	yes	down
ENSSSCG00000024084	SNORD12C	0.705212244	-0.503870572	0.004180126	0.475451096	yes	down
ENSSSCG00000024166	SLC2A6	0.428721766	-1.221886432	0.035479051	0.707740306	yes	down
ENSSSCG00000024179	NAGS	0.777978515	-0.362197781	0.046285181	0.72213448	yes	down
ENSSSCG00000024184	FASTK	0.812865723	-0.298911042	0.026251808	0.684917502	yes	down
ENSSSCG00000024223	ARHGEF16	0.734031211	-0.446086687	0.026368198	0.685905976	yes	down
ENSSSCG00000024320	TFF2	0.292953703	-1.771255409	0.017506851	0.67109181	yes	down
ENSSSCG00000024467	—	3.646953978	1.866691994	0.046926247	0.72213448	yes	up
ENSSSCG00000024476	CES3	0.649690496	-0.622175495	0.004653143	0.505429436	yes	down
ENSSSCG00000024481	PDE4DIP	0.788288448	-0.343204462	0.029424285	0.696976005	yes	down
ENSSSCG00000024570	KDM4B	0.667026648	-0.584183696	0.030122219	0.700714589	yes	down
ENSSSCG00000024578	PHLDB1	0.758214154	-0.399322707	0.00953759	0.603302839	yes	down
ENSSSCG00000024622	ZNF667	1.236154349	0.305858893	0.043504591	0.72213448	yes	up
ENSSSCG00000024665	TRAPPC12	0.668793561	-0.580367137	0.0004636	0.238844184	yes	down

ENSSSCG00000024697	TMEM125	0.817162186	-0.29130565	0.043290001	0.72213448	yes	down
ENSSSCG00000024777	HDAC4	0.750630338	-0.413825495	0.022953171	0.675846472	yes	down
ENSSSCG00000024860	SLC5A1	0.311635318	-1.682069349	2.38E-05	0.055287609	yes	down
ENSSSCG00000024872	CPT1C	1.567368795	0.64834468	0.008995549	0.591348223	yes	up
ENSSSCG00000024881	TCP11L2	1.210010831	0.275019961	0.041043322	0.715680171	yes	up
ENSSSCG00000024927	STX12	1.219662235	0.286481673	0.007764419	0.565052652	yes	up
ENSSSCG00000024973	GBP1	1.697870982	0.763726835	0.010778006	0.617982236	yes	up
ENSSSCG00000025020	PCSK9	0.559652678	-0.837396331	0.028026753	0.693082926	yes	down
ENSSSCG00000025071	BTNL2	0.365832111	-1.450746383	0.019995721	0.67109181	yes	down
ENSSSCG00000025152	CEP89	0.805173671	-0.312628098	0.025971346	0.683249021	yes	down
ENSSSCG00000025308	IL17D	0.328655772	-1.605350771	0.038883275	0.714516217	yes	down
ENSSSCG00000025393	SUSD2	0.662727635	-0.593512016	0.006572588	0.527035893	yes	down
ENSSSCG00000025434	CGA	3.97349548	1.990408703	0.021678842	0.67241349	yes	up
ENSSSCG00000025602	DOCK4	1.321825984	0.402532262	0.026753064	0.69011278	yes	up
ENSSSCG00000025610	AGAP3	0.757349006	-0.40096981	0.03751058	0.707740306	yes	down
ENSSSCG00000025689	DVL2	0.742760946	-0.429030134	0.019261746	0.67109181	yes	down
ENSSSCG00000025770	ST6GAL1	1.386754611	0.471712522	0.039704283	0.714830695	yes	up
ENSSSCG00000025794	FBRS	0.78804539	-0.343649366	0.026443459	0.685939631	yes	down
ENSSSCG00000025826	BOC	0.641030141	-0.641535902	0.001467227	0.331861665	yes	down
ENSSSCG00000025855	RNMT	1.220390715	0.287343108	0.011082068	0.617982236	yes	up
ENSSSCG00000026043	TGM3	0.279854055	-1.837253441	0.005048822	0.513485708	yes	down
ENSSSCG00000026068	GRHL1	0.39337553	-1.346020878	0.008991679	0.591348223	yes	down
ENSSSCG00000026078	GLCE	1.263223969	0.337110451	0.007768827	0.565052652	yes	up
ENSSSCG00000026092	SREBF2	0.776379987	-0.365165166	0.038126444	0.709706918	yes	down
ENSSSCG00000026108	CDC42EP1	0.729376489	-0.455264399	0.048324988	0.72213448	yes	down
ENSSSCG00000026142	TNK1	0.738368283	-0.437587512	0.048546618	0.72213448	yes	down
ENSSSCG00000026152	PRKD2	0.782618647	-0.353618611	0.006015027	0.526667284	yes	down
ENSSSCG00000026248	PAQR4	0.713732587	-0.486544451	0.049178709	0.724153784	yes	down
ENSSSCG00000026268	DLG5	0.727257902	-0.459461027	0.006226617	0.527035893	yes	down
ENSSSCG00000026387	REEP5_6	0.601263081	-0.733931719	0.025959363	0.683249021	yes	down
ENSSSCG00000026392	BSDC1	0.796952411	-0.327434517	0.005901827	0.523737709	yes	down
ENSSSCG00000026422	GPER1	0.554828916	-0.849885115	0.001161151	0.312531581	yes	down
ENSSSCG00000026454	PMAIP1	1.29224404	0.369878548	0.022963795	0.675846472	yes	up
ENSSSCG00000026498	R3HDM2	0.80234978	-0.317696786	0.01296692	0.635895234	yes	down
ENSSSCG00000026512	CALC	1.798144853	0.846509244	0.043754723	0.72213448	yes	up
ENSSSCG00000026729	TMEM150C	0.775687052	-0.366453375	0.004002891	0.475451096	yes	down
ENSSSCG00000026819	NID1	0.76811059	-0.380614054	0.033431976	0.703664106	yes	down
ENSSSCG00000026849	CCNO	0.422605934	-1.242615069	0.017412329	0.67109181	yes	down
ENSSSCG00000026951	PSMB8	1.399708461	0.485126366	0.030432635	0.700958898	yes	up
ENSSSCG00000026959	NPAT	1.340401421	0.422665121	0.011610577	0.623024075	yes	up
ENSSSCG00000026969	KRAS	1.227900701	0.296193896	0.04809872	0.72213448	yes	up

ENSSSCG00000026978	ROS1	0.655792802	-0.608688029	0.043321948	0.72213448	yes	down
ENSSSCG00000027016	C19orf47	0.800168934	-0.321623477	0.046579498	0.72213448	yes	down
ENSSSCG00000027060	TBX2	0.749184413	-0.416607211	0.003936957	0.474147655	yes	down
ENSSSCG00000027076	ZFTRAF1	0.728815738	-0.456373981	0.004119115	0.475451096	yes	down
ENSSSCG00000027189	PPP1R12C	0.734740477	-0.44469334	0.040994574	0.715680171	yes	down
ENSSSCG00000027278	ARL6	1.239587934	0.309860617	0.03523023	0.707740306	yes	up
ENSSSCG00000027299	PANK4	0.666699589	-0.584891257	0.003834923	0.471035234	yes	down
ENSSSCG00000027302	PKP3	0.774511983	-0.368640534	0.027459408	0.693082926	yes	down
ENSSSCG00000027322	PGLYRP3	0.639924312	-0.644026817	0.049944346	0.728012358	yes	down
ENSSSCG00000027331	COL6A3	0.726327985	-0.461306928	0.002360811	0.379220559	yes	down
ENSSSCG00000027371	PCDHGA4	0.770389473	-0.376340106	0.019569918	0.67109181	yes	down
ENSSSCG00000027480	KLF10	1.241023776	0.311530755	0.006239335	0.527035893	yes	up
ENSSSCG00000027506	POLD3	1.287952629	0.365079532	0.011325778	0.617982236	yes	up
ENSSSCG00000027515	FCGBP	0.573945771	-0.801013664	0.022299296	0.673830277	yes	down
ENSSSCG00000027902	ZMPSTE24	1.232606533	0.301712343	0.030608832	0.700958898	yes	up
ENSSSCG00000027916	PEX10	0.711771711	-0.490513499	0.010650501	0.617982236	yes	down
ENSSSCG00000027986	ABCB8	0.77787463	-0.362390441	0.041679479	0.717056849	yes	down
ENSSSCG00000027992	MST1R	0.809803497	-0.304356221	0.036435459	0.707740306	yes	down
ENSSSCG00000028036	MAP3K10	0.692944303	-0.529188699	0.025455635	0.679826869	yes	down
ENSSSCG00000028076	ZBTB7C	0.824522566	-0.278369117	0.030442705	0.700958898	yes	down
ENSSSCG00000028185	FGD3	0.772940292	-0.371571122	0.026105806	0.684843544	yes	down
ENSSSCG00000028210	NT5C	1.298012755	0.37630456	0.010871911	0.617982236	yes	up
ENSSSCG00000028278	POLR2A	0.724210437	-0.465519127	0.033820589	0.703664106	yes	down
ENSSSCG00000028327	RHOBTB1	0.805520254	-0.312007231	0.018293156	0.67109181	yes	down
ENSSSCG00000028559	LZTS1	0.743702096	-0.427203257	0.016978852	0.67109181	yes	down
ENSSSCG00000028593	TEPSIN	0.699138867	-0.516349054	0.017229022	0.67109181	yes	down
ENSSSCG00000028612	PTPRS	0.732898716	-0.448314259	0.002383478	0.379220559	yes	down
ENSSSCG00000028646	MMADHC	1.212336174	0.277789806	0.010933195	0.617982236	yes	up
ENSSSCG00000028674	CR2/CD21	0.448623519	-1.156422838	0.021785192	0.673416588	yes	down
ENSSSCG00000028804	CCDC181	2.332847233	1.222091835	2.31E-05	0.055287609	yes	up
ENSSSCG00000028806	RPS6KA4	0.735994013	-0.442234064	0.008277099	0.578565651	yes	down
ENSSSCG00000028858	FAM83C	0.462788881	-1.111573893	0.003311527	0.445536806	yes	down
ENSSSCG00000028905	TNIK	0.815688391	-0.293909975	0.040121785	0.714830695	yes	down
ENSSSCG00000028908	DYNLT3	1.234370467	0.30377545	0.046694751	0.72213448	yes	up
ENSSSCG00000028995	GRO	0.675585008	-0.565790781	0.006812948	0.528701837	yes	down
ENSSSCG00000029073	CACNB4	2.268364764	1.181652651	0.0090557	0.591394599	yes	up
ENSSSCG00000029102	MED24	0.826701885	-0.274560918	0.038723832	0.713927357	yes	down
ENSSSCG00000029189	DCHS1	0.764744757	-0.386949784	0.026775763	0.69011278	yes	down
ENSSSCG00000029199	SCN4B	0.659978654	-0.599508732	0.043325895	0.72213448	yes	down
ENSSSCG00000029236	ZBTB7A	0.786133013	-0.347154659	0.028007694	0.693082926	yes	down
ENSSSCG00000029268	TOPAZ1	0.648125014	-0.62565598	0.022635563	0.675846472	yes	down

ENSSSCG00000029291	PIGZ	1.388119409	0.473131677	0.047212154	0.72213448	yes	up
ENSSSCG00000029388	PDE2A	0.617590868	-0.695276675	0.000954678	0.310915441	yes	down
ENSSSCG00000029414	FCN	2.508722131	1.326952685	0.005824823	0.523737709	yes	up
ENSSSCG00000029570	PXDN	0.676192911	-0.564493204	0.001183028	0.312531581	yes	down
ENSSSCG00000029598	FAM124A	0.33984094	-1.557068434	0.001792305	0.335087945	yes	down
ENSSSCG00000029705	PPIE	10.3377602	3.369851738	0.032909083	1	yes	up
ENSSSCG00000029711	ZNF518B	0.822937734	-0.281144819	0.022356714	0.673830277	yes	down
ENSSSCG00000029745	FAM193A	0.716460977	-0.481039966	0.027669078	0.693082926	yes	down
ENSSSCG00000029813	TSPAN5	1.331673282	0.413240169	0.022158748	0.673830277	yes	up
ENSSSCG00000029843	NPHP4	0.613195248	-0.705581577	0.005900364	0.523737709	yes	down
ENSSSCG00000029857	EIF2S3	1.530464058	0.613969164	0.011341993	0.617982236	yes	up
ENSSSCG00000029944	FASN	0.589315513	-0.762887851	0.008631346	0.58854989	yes	down
ENSSSCG00000030030	FBH1	0.759309255	-0.397240502	0.03890838	0.714516217	yes	down
ENSSSCG00000030095	ZBTB16	2.130239844	1.091015873	0.009821351	0.603302839	yes	up
ENSSSCG00000030137	LMNTD2	0.303321536	-1.721080161	0.024249793	0.67597251	yes	down
ENSSSCG00000030196	CUL9	0.729479318	-0.455061019	0.014064842	0.651736454	yes	down
ENSSSCG00000030277	ENDOV	0.690316652	-0.53466981	0.012889621	0.635895234	yes	down
ENSSSCG00000030368	HSPA1L	0.448252092	-1.157617778	0.003011752	0.419992148	yes	down
ENSSSCG00000030371	SERPINA	0.045159737	-4.468819107	0.016035856	0.67109181	yes	down
ENSSSCG00000030395	ASB5	2.223241826	1.152664881	0.04144456	0.715680171	yes	up
ENSSSCG00000030567	SSR3	1.294937511	0.37288248	0.011723031	0.623024075	yes	up
ENSSSCG00000030577	PBDC1	1.214928933	0.280871926	0.029032903	0.696976005	yes	up
ENSSSCG00000030668	UQCC2	1.23967048	0.309956685	0.04786324	0.72213448	yes	up
ENSSSCG00000030827	FGFR3	0.607936191	-0.718008188	0.006436513	0.527035893	yes	down
ENSSSCG00000030831	DENND3	0.752544933	-0.410150371	0.02397722	0.67597251	yes	down
ENSSSCG00000030857	LENG8	0.807512557	-0.308443401	0.047017167	0.72213448	yes	down
ENSSSCG00000031003	CKB	0.248529673	-2.008509983	0.046565126	0.72213448	yes	down
ENSSSCG00000031103	TBK1	1.2202405	0.28716552	0.016507376	0.67109181	yes	up
ENSSSCG00000031221	FEZF1	0.069605479	-3.844655323	0.01857904	1	yes	down
ENSSSCG00000031302	CTBP1	0.756955208	-0.401720161	0.02825848	0.695002653	yes	down
ENSSSCG00000031360	ADO	0.540009347	-0.888943715	0.005435375	0.516973839	yes	down
ENSSSCG00000031423	UPK3A	2.046468467	1.033136437	4.67E-05	0.070265172	yes	up
ENSSSCG00000031441	GRAMD4	0.742803329	-0.428947814	0.043187248	0.72213448	yes	down
ENSSSCG00000031531	TSPAN10	0.504678897	-0.986562332	0.037813595	0.709061263	yes	down
ENSSSCG00000031580	LLPH	1.249371866	0.321202948	0.026993333	0.690849599	yes	up
ENSSSCG00000031630	DPH7	0.702917193	-0.508573351	0.036817024	0.707740306	yes	down
ENSSSCG00000031657	JUND	0.70129023	-0.511916465	0.037189853	0.707740306	yes	down
ENSSSCG00000031706	MICAL	0.787013269	-0.345540134	0.035454487	0.707740306	yes	down
ENSSSCG00000031776	TULP4	0.782038287	-0.354688854	0.017130776	0.67109181	yes	down
ENSSSCG00000031819	TP53I11	1.233933194	0.303264288	0.026043666	0.684181115	yes	up
ENSSSCG00000031855	CUGBP	7.507396208	2.908312624	0.000237768	0.163328898	yes	up

ENSSSCG00000031870	ZNF783	0.738281189	-0.437757694	0.01974671	0.67109181	yes	down
ENSSSCG00000031876	LDAF1	1.639666897	0.713402757	0.005997203	0.526667284	yes	up
ENSSSCG00000031888	DDIT4	0.702393005	-0.509649617	0.046175715	0.72213448	yes	down
ENSSSCG00000031897	GIMAP7	1.421623499	0.507539434	0.031559642	0.703664106	yes	up
ENSSSCG00000031925	SHANK	0.717407146	-0.47913598	0.036521254	0.707740306	yes	down
ENSSSCG00000031938	NEDD8	1.241920514	0.31257284	0.031395447	0.703664106	yes	up
ENSSSCG00000031941	MED22	0.738807132	-0.436730302	0.019077183	0.67109181	yes	down
ENSSSCG00000031999	GPR18	1.622847314	0.69852727	0.033575564	0.703664106	yes	up
ENSSSCG00000032003	GST	1.218632092	0.285262638	0.020482637	0.67109181	yes	up
ENSSSCG00000032080	ZNF35	1.227849463	0.296133694	0.040927029	0.715680171	yes	up
ENSSSCG00000032082	BMP8	0.391788951	-1.351851382	0.027502988	0.693082926	yes	down
ENSSSCG00000032164	PEA15	1.206162739	0.270424573	0.002945649	0.41873231	yes	up
ENSSSCG00000032177	MYO3B	0.248998813	-2.005789229	0.028414458	0.695002653	yes	down
ENSSSCG00000032202	—	1.354886263	0.438171748	0.010441137	0.617982236	yes	up
ENSSSCG00000032223	EIF1AX	1.351635732	0.434706396	0.015127944	0.656815274	yes	up
ENSSSCG00000032262	IDUA	0.763505213	-0.389290087	0.04919576	0.724153784	yes	down
ENSSSCG00000032279	SAPCD2	0.755076398	-0.405305472	0.011853995	0.623024075	yes	down
ENSSSCG00000032298	ABCC11	0.154646053	-2.692958084	0.03585747	1	yes	down
ENSSSCG00000032341	AIF1	1.552233335	0.634345443	0.023299109	0.675846472	yes	up
ENSSSCG00000032344	KIAA1143	1.518091754	0.60225899	0.000699673	0.268488108	yes	up
ENSSSCG00000032364	SELENOH	1.211757174	0.277100624	0.042703941	0.72213448	yes	up
ENSSSCG00000032422	TCAF2-like	0.602569591	-0.730800227	0.012287408	0.631103805	yes	down
ENSSSCG00000032475	CEP44	1.361476417	0.445171992	0.029307084	0.696976005	yes	up
ENSSSCG00000032532	CHRM2	1.554871063	0.636794951	0.037061358	0.707740306	yes	up
ENSSSCG00000032633	FAM53A	0.71042783	-0.493239996	0.021078614	0.67109181	yes	down
ENSSSCG00000032761	LAMA5	0.675465631	-0.566045731	0.036205377	0.707740306	yes	down
ENSSSCG00000032785	SLC25A41	0.193511064	-2.36951204	0.045494881	0.72213448	yes	down
ENSSSCG00000032820	BRD1	0.756500625	-0.402586821	0.030224385	0.700714589	yes	down
ENSSSCG00000032833	CD320	1.303736734	0.382652572	0.046959968	0.72213448	yes	up
ENSSSCG00000032911	DUSP8	0.574147945	-0.800505559	0.039553874	0.714830695	yes	down
ENSSSCG00000032964	STK11IP	0.71079132	-0.49250203	0.004714471	0.505429436	yes	down
ENSSSCG00000032986	AKAP17A	0.786339004	-0.346776678	0.039071764	0.714830695	yes	down
ENSSSCG00000032990	—	0.18523065	-2.432605253	0.036747609	0.707740306	yes	down
ENSSSCG00000033006	MDP1	60.24787713	5.912838503	0.033257207	0.703664106	yes	up
ENSSSCG00000033027	CIAO2B	1.208722077	0.273482562	0.011610052	0.623024075	yes	up
ENSSSCG00000033059	CASKIN1	0.577384331	-0.79239614	0.005026687	0.513485708	yes	down
ENSSSCG00000033082	HGS	0.739893228	-0.434611001	0.023855491	0.67597251	yes	down
ENSSSCG00000033114	—	0.329119576	-1.603316252	0.013951138	0.651736454	yes	down
ENSSSCG00000033142	ENDOU	0.552586106	-0.855728807	0.036289533	0.707740306	yes	down
ENSSSCG00000033154	ARL6IP5	1.223489656	0.291001904	0.004691629	0.505429436	yes	up
ENSSSCG00000033171	MAPK11	0.499696919	-1.000874771	0.000651861	0.262827653	yes	down

ENSSSCG00000033183	MCEMP1	3.037234008	1.602758067	0.027753494	0.693082926	yes	up
ENSSSCG00000033222	TRIM14	0.811929984	-0.300572771	0.00988706	0.603302839	yes	down
ENSSSCG00000033232	KRAB	1.585125708	0.664597258	0.021470561	0.67241349	yes	up
ENSSSCG00000033299	FLNA	0.768366264	-0.380133918	0.002201074	0.364193212	yes	down
ENSSSCG00000033301	THAP7	0.790004476	-0.340067267	0.022243987	0.673830277	yes	down
ENSSSCG00000033313	SPMIP4	7.282381937	2.864410407	0.025210149	0.679826869	yes	up
ENSSSCG00000033335	AKIP1	0.803894647	-0.314921651	0.028349632	0.695002653	yes	down
ENSSSCG00000033363	MSX	0.436273807	-1.196694234	0.027533717	0.693082926	yes	down
ENSSSCG00000033374	MRPL20	1.33179852	0.413375842	0.002514915	0.379220559	yes	up
ENSSSCG00000033457	CCL15_23	2.010900298	1.007841553	0.031323294	0.703664106	yes	up
ENSSSCG00000033493	CACNA1B	0.497437763	-1.00741206	0.0201171	0.67109181	yes	down
ENSSSCG00000033542	VSTM1	0.663732215	-0.591326797	0.037193622	0.707740306	yes	down
ENSSSCG00000033577	MED30	1.246767723	0.318192711	0.038584817	0.71278147	yes	up
ENSSSCG00000033608	LOXL2	0.670279891	-0.577164442	9.06E-05	0.098892808	yes	down
ENSSSCG00000033643	NLGN	0.381763231	-1.389249937	0.032296404	0.703664106	yes	down
ENSSSCG00000033691	RAB26	0.565409307	-0.822632464	0.012512411	0.632336999	yes	down
ENSSSCG00000033720	INF2	0.705919446	-0.502424532	0.014511825	0.652664121	yes	down
ENSSSCG00000033760	LTBP4	0.760481017	-0.395015859	0.004712851	0.505429436	yes	down
ENSSSCG00000033883	ltaE	0.418067924	-1.258190738	0.0011226	0.312531581	yes	down
ENSSSCG00000033937	DYNC2H1	1.310466717	0.390080713	0.034176384	0.704424911	yes	up
ENSSSCG00000033946	DIDO1	0.784192917	-0.350719483	0.014238542	0.652054887	yes	down
ENSSSCG00000034039	SLC11A2	1.526859009	0.610566849	0.039259543	0.714830695	yes	up
ENSSSCG00000034096	DMAP1	0.761061963	-0.393914177	0.034559484	0.707740306	yes	down
ENSSSCG00000034102	DGAT2	0.565771831	-0.821707745	0.009969748	0.604501428	yes	down
ENSSSCG00000034114	GPR68	0.649771157	-0.62199639	0.020711523	0.67109181	yes	down
ENSSSCG00000034262	FIGN	0.573933013	-0.801045733	0.040074741	0.714830695	yes	down
ENSSSCG00000034266	CACNA1H	0.62059032	-0.6882869	0.006578677	0.527035893	yes	down
ENSSSCG00000034297	HES2	0.585746876	-0.771650741	0.000354218	0.205302825	yes	down
ENSSSCG00000034353	SMG8	1.278490344	0.354441264	0.038340466	0.712525669	yes	up
ENSSSCG00000034360	CELSR2	0.677434446	-0.561846749	0.011131906	0.617982236	yes	down
ENSSSCG00000034440	MMP28	0.609841902	-0.713492814	0.024857834	0.67891626	yes	down
ENSSSCG00000034566	NPDC1	0.588797529	-0.764156477	0.018421225	0.67109181	yes	down
ENSSSCG00000034653	DLX4	0.63228147	-0.661361155	0.024081985	0.67597251	yes	down
ENSSSCG00000034692	SH3BP4	0.682411879	-0.551285335	0.020939799	0.67109181	yes	down
ENSSSCG00000034795	SCAMP1	1.209001877	0.273816484	0.022949044	0.675846472	yes	up
ENSSSCG00000034854	SEC16A	0.771112065	-0.374987555	0.044855024	0.72213448	yes	down
ENSSSCG00000034914	CD163	4.509919072	2.173101545	0.012824265	0.635895234	yes	up
ENSSSCG00000034943	GDF6	0.19120965	-2.386772757	0.021908297	0.673830277	yes	down
ENSSSCG00000034952	DPM1	1.221794437	0.289001577	0.037280216	0.707740306	yes	up
ENSSSCG00000034958	MIS12	1.265648655	0.339876967	0.001750432	0.335087945	yes	up
ENSSSCG00000034994	AKAP1	0.757077021	-0.401488014	0.042679243	0.72213448	yes	down

ENSSSCG00000035066	MYOC	0.488791824	-1.032707941	0.045910675	0.72213448	yes	down
ENSSSCG00000035152	TEF	0.454443662	-1.137826642	0.000288122	0.178126396	yes	down
ENSSSCG00000035284	BMF	1.412773529	0.498530217	0.001777091	0.335087945	yes	up
ENSSSCG00000035477	KRAB	3.676946233	1.87850808	0.015355223	0.657721277	yes	up
ENSSSCG00000035488	WDR44	1.225044943	0.292834679	0.030581979	0.700958898	yes	up
ENSSSCG00000035555	BTBD6	0.664180565	-0.590352588	0.013038186	0.635895234	yes	down
ENSSSCG00000035574	ULBP	0.748843694	-0.417263479	0.029157553	0.696976005	yes	down
ENSSSCG00000035657	DIP2A	0.694811149	-0.52530719	0.00284462	0.417894019	yes	down
ENSSSCG00000035669	AHNAK	0.487136076	-1.037603264	0.005505097	0.52093383	yes	down
ENSSSCG00000035673	HBP1	1.20306986	0.26672042	0.041286093	0.715680171	yes	up
ENSSSCG00000035706	HSD17B12	0.644514665	-0.63371491	0.041331329	0.715680171	yes	down
ENSSSCG00000035733	PLXNB2	0.743042244	-0.42848386	0.031063473	0.703664106	yes	down
ENSSSCG00000035739	SLC26A1	0.653860515	-0.61294519	0.036958257	0.707740306	yes	down
ENSSSCG00000035770	ZNF444	0.681424365	-0.553374562	0.023270196	0.675846472	yes	down
ENSSSCG00000035771	MRPS22	1.219387022	0.286156097	0.037916861	0.709061263	yes	up
ENSSSCG00000035833	NKPD1	0.309566441	-1.691679011	0.04236945	0.721453604	yes	down
ENSSSCG00000035839	TOP3A	0.782322355	-0.354164904	0.043255395	0.72213448	yes	down
ENSSSCG00000035854	RAB11FIP3	0.718577141	-0.476785054	0.017522771	0.67109181	yes	down
ENSSSCG00000035919	—	0.337108662	-1.568714396	0.000574024	0.241964073	yes	down
ENSSSCG00000035934	TXNDC16	1.50656917	0.591266912	0.000172592	0.133112815	yes	up
ENSSSCG00000036027	TPRN	0.710705358	-0.49267652	0.048283188	0.72213448	yes	down
ENSSSCG00000036086	PAX5	0.547444039	-0.869216598	0.026708773	0.69011278	yes	down
ENSSSCG00000036135	COL1A1	0.763055445	-0.390140205	0.012138262	0.628850139	yes	down
ENSSSCG00000036178	CCDC167	1.372517654	0.456824706	0.022217362	0.673830277	yes	up
ENSSSCG00000036206	DIPK2A	1.471965604	0.55774396	0.001768254	0.335087945	yes	up
ENSSSCG00000036237	ITGA7	0.682313276	-0.551493806	0.002514596	0.379220559	yes	down
ENSSSCG00000036307	OAF	0.718264243	-0.477413398	0.019685165	0.67109181	yes	down
ENSSSCG00000036326	LATS2	0.757621351	-0.400451105	0.020467034	0.67109181	yes	down
ENSSSCG00000036363	PDE4C	0.699922054	-0.514733829	0.047283519	0.72213448	yes	down
ENSSSCG00000036377	OVOL2	0.656593087	-0.606928534	0.021716487	0.67241349	yes	down
ENSSSCG00000036385	BET1	1.245402736	0.316612353	0.044689779	0.72213448	yes	up
ENSSSCG00000036482	ZSWIM7	0.768986929	-0.378969018	0.046784522	0.72213448	yes	down
ENSSSCG00000036573	SPDEF	0.640024447	-0.643801082	0.013747535	0.651736454	yes	down
ENSSSCG00000036722	MALSU1	1.221736448	0.288933101	0.022984107	0.675846472	yes	up
ENSSSCG00000036742	KLF15	0.614257564	-0.703084376	0.003706251	0.458265629	yes	down
ENSSSCG00000036776	LRRC75A	0.708590956	-0.496975042	0.032007307	0.703664106	yes	down
ENSSSCG00000036812	MPRIP	0.723428604	-0.467077452	0.025955256	0.683249021	yes	down
ENSSSCG00000036844	GPR171	1.518984808	0.603107441	0.006778971	0.528701837	yes	up
ENSSSCG00000036887	CLPB	0.784220555	-0.350668639	0.033955862	0.703664106	yes	down
ENSSSCG00000036964	FAM83H	0.731948394	-0.45018616	0.032295042	0.703664106	yes	down
ENSSSCG00000037071	—	1.396432562	0.481745903	0.04763404	0.72213448	yes	up

ENSSSCG00000037153	FEM1A	0.721093083	-0.471742591	0.028604628	0.69504785	yes	down
ENSSSCG00000037195	FOXF2	0.752258063	-0.41070043	0.023553004	0.675846472	yes	down
ENSSSCG00000037254	RNPC3	1.486933833	0.57234045	0.007922932	0.571776715	yes	up
ENSSSCG00000037269	LANCL3	0.668416757	-0.581180194	0.047597734	0.72213448	yes	down
ENSSSCG00000037313	MTIF3	0.788545512	-0.34273407	0.027060469	0.690849599	yes	down
ENSSSCG00000037318	TRABD2B	0.697047648	-0.520670817	0.018602027	0.67109181	yes	down
ENSSSCG00000037349	MRGPRX	3.252016485	1.701334571	0.011847646	0.623024075	yes	up
ENSSSCG00000037360	CST3	1.408507721	0.494167472	0.011147463	0.617982236	yes	up
ENSSSCG00000037395	CLIC3	0.48467216	-1.044918879	0.017917061	0.67109181	yes	down
ENSSSCG00000037399	PRORSD1	1.439937408	0.526006101	0.001312509	0.313270149	yes	up
ENSSSCG00000037473	DDX19	4.435330085	2.149041477	0.024826821	0.67891626	yes	up
ENSSSCG00000037514	BMP8	0.399443196	-1.323937742	0.010712538	0.617982236	yes	down
ENSSSCG00000037539	SORCS2	0.580575227	-0.78444508	0.020010403	0.67109181	yes	down
ENSSSCG00000037557	DRC3	0.683747286	-0.548464894	0.019544533	0.67109181	yes	down
ENSSSCG00000037559	ACOT11	0.743011571	-0.428543417	0.011727285	0.623024075	yes	down
ENSSSCG00000037561	ATP11A	0.711601457	-0.490858631	0.00215259	0.362946196	yes	down
ENSSSCG00000037562	SLC2A4RG	0.51805892	-0.948811907	0.001790378	0.335087945	yes	down
ENSSSCG00000037602	SNRPB2	1.249776589	0.32167022	0.045493499	0.72213448	yes	up
ENSSSCG00000037637	GGH	1.330917885	0.412421563	0.048656323	0.72213448	yes	up
ENSSSCG00000037671	PACS2	0.662592138	-0.593807009	0.008507518	0.584403443	yes	down
ENSSSCG00000037682	HFE	0.704566898	-0.5051914	0.008801632	0.589351395	yes	down
ENSSSCG00000037762	TBX1	0.655206874	-0.609977602	0.04542451	0.72213448	yes	down
ENSSSCG00000037878	AHSP	13.86494527	3.793370016	0.002350989	0.379220559	yes	up
ENSSSCG00000037880	NSMF	0.711323892	-0.491421473	0.037373997	0.707740306	yes	down
ENSSSCG00000038025	NRBP2	0.69413994	-0.526701553	0.029272679	0.696976005	yes	down
ENSSSCG00000038044	GYG2	0.612574662	-0.707042402	0.02003843	0.67109181	yes	down
ENSSSCG00000038089	COL18A1	0.566703078	-0.819335055	0.000319726	0.191288684	yes	down
ENSSSCG00000038094	GPX7	0.714811035	-0.484366188	0.003466683	0.447112352	yes	down
ENSSSCG00000038110	TIFAB	1.645963764	0.718932575	0.005565045	0.523737709	yes	up
ENSSSCG00000038149	KCNE4	0.489228753	-1.031418898	0.001059621	0.310915441	yes	down
ENSSSCG00000038164	RAI1	0.641234622	-0.641075772	0.009278439	0.598675994	yes	down
ENSSSCG00000038190	MPST	0.703669566	-0.507029978	0.032182557	0.703664106	yes	down
ENSSSCG00000038220	RXRA	0.725269204	-0.463411504	0.01891802	0.67109181	yes	down
ENSSSCG00000038296	SH3RF3	0.62255636	-0.683723646	0.042281765	0.721435055	yes	down
ENSSSCG00000038437	EHMT1	0.74665236	-0.421491411	0.014373438	0.652664121	yes	down
ENSSSCG00000038460	FOXF1	0.829621818	-0.26947426	0.008376263	0.578565651	yes	down
ENSSSCG00000038487	TMPO	1.436839024	0.522898439	0.039986707	0.714830695	yes	up
ENSSSCG00000038508	SPTBN2	0.666788683	-0.584698477	0.048433982	0.72213448	yes	down
ENSSSCG00000038509	NOTCH1	0.718598306	-0.47674256	0.01697649	0.67109181	yes	down
ENSSSCG00000038521	CHAC1	0.55503271	-0.849355297	0.019327988	0.67109181	yes	down
ENSSSCG00000038526	FBXL18	0.679892202	-0.556622073	0.021096886	0.67109181	yes	down

ENSSSCG00000038549	ZFP36L2	0.787731991	-0.344223228	0.017326717	0.67109181	yes	down
ENSSSCG00000038604	DOCK6	0.714998526	-0.483987827	0.015298624	0.657271396	yes	down
ENSSSCG00000038631	TCFL5	0.659985403	-0.599493979	0.017729076	0.67109181	yes	down
ENSSSCG00000038679	CNTRL	1.476487522	0.562169165	0.00429692	0.475451096	yes	up
ENSSSCG00000038765	FAM89A	0.742631017	-0.429282523	0.036381762	0.707740306	yes	down
ENSSSCG00000038807	SCAF1	0.825050144	-0.27744629	0.039966792	0.714830695	yes	down
ENSSSCG00000038856	TRIM8	0.789260239	-0.341427023	0.015901204	0.67109181	yes	down
ENSSSCG00000038895	DCTN6	1.277976103	0.35386086	0.035036823	0.707740306	yes	up
ENSSSCG00000038914	SEC61B	1.235677205	0.305301919	0.045068747	0.72213448	yes	up
ENSSSCG00000038946	SLC17A8	0.232003307	-2.107782726	0.011335488	0.617982236	yes	down
ENSSSCG00000038964	COX19	0.697236402	-0.520280203	0.041189703	0.715680171	yes	down
ENSSSCG00000038992	TUBB6	0.813952007	-0.296984363	0.040838075	0.715680171	yes	down
ENSSSCG00000039025	FREM1	0.452258047	-1.144781923	0.000145075	0.133112815	yes	down
ENSSSCG00000039057	ALKAL2	1.645649032	0.718656684	0.048649205	0.72213448	yes	up
ENSSSCG00000039094	PLEC	0.651325787	-0.61854875	0.001682784	0.335087945	yes	down
ENSSSCG00000039139	AKT1	0.728418702	-0.457160131	0.021232867	0.672023871	yes	down
ENSSSCG00000039169	CER1	0.476511577	-1.069416828	0.039238341	0.714830695	yes	down
ENSSSCG00000039196	POLR2I	1.458502918	0.544488272	0.001408323	0.326403208	yes	up
ENSSSCG00000039214	IL1B	2.400103552	1.263096652	0.013099276	0.635895234	yes	up
ENSSSCG00000039252	TCN1	0.311760434	-1.681490251	0.026204888	0.684917502	yes	down
ENSSSCG00000039261	WSCD2	0.593918329	-0.751663537	0.043873168	0.72213448	yes	down
ENSSSCG00000039264	ABCA2	0.629290117	-0.668202808	0.010183831	0.615242696	yes	down
ENSSSCG00000039330	ANO8	0.783277158	-0.352405208	0.01453561	0.652664121	yes	down
ENSSSCG00000039332	SEC16B	0.615957116	-0.699098184	0.002513095	0.379220559	yes	down
ENSSSCG00000039338	SUGT1	1.24938402	0.321216982	0.029301382	0.696976005	yes	up
ENSSSCG00000039344	ABCC4	16.8575951	4.075326832	0.00068977	0.268488108	yes	up
ENSSSCG00000039348	H1-0	1.382433301	0.467209875	0.00508827	0.513485708	yes	up
ENSSSCG00000039392	SNPH	0.523530686	-0.933653993	0.005135866	0.513485708	yes	down
ENSSSCG00000039416	CXCR4	1.338374529	0.420481894	0.009973442	0.604501428	yes	up
ENSSSCG00000039472	SLC30A1	1.275091296	0.350600548	0.01791784	0.67109181	yes	up
ENSSSCG00000039476	DHRSX	0.731575631	-0.450921076	0.002957564	0.41873231	yes	down
ENSSSCG00000039500	MADCAM1	1.479144543	0.564763041	0.022356404	0.673830277	yes	up
ENSSSCG00000039539	DHTKD1	0.719078783	-0.475778253	0.031587722	0.703664106	yes	down
ENSSSCG00000039541	ANKRD11	0.738538065	-0.437255813	0.04913155	0.724153784	yes	down
ENSSSCG00000039713	ANKRD9	0.707811925	-0.498562028	0.024224691	0.67597251	yes	down
ENSSSCG00000039714	MIPOL1	1.309076381	0.388549277	0.032671155	0.703664106	yes	up
ENSSSCG00000039731	GLRX2	1.400981206	0.486437603	0.006309778	0.527035893	yes	up
ENSSSCG00000039741	—	4.374336087	2.129064069	0.010923702	0.617982236	yes	up
ENSSSCG00000039745	HMOX1	1.257349666	0.330385916	0.024696298	0.67891626	yes	up
ENSSSCG00000039763	CMBL	1.227056632	0.295201834	0.046396514	0.72213448	yes	up
ENSSSCG00000039767	TH	0.121038621	-3.046460642	0.018113054	0.67109181	yes	down

ENSSSCG00000039774	KRAB	1.725521012	0.787032041	0.016631509	0.67109181	yes	up
ENSSSCG00000039778	—	1.436157067	0.52221354	0.045223347	0.72213448	yes	up
ENSSSCG00000039807	SECTM1	1.409248326	0.494925854	0.032833421	0.703664106	yes	up
ENSSSCG00000039862	TRIB3	0.542409725	-0.882545051	0.017535683	0.67109181	yes	down
ENSSSCG00000039909	ERN1	0.800733749	-0.320605482	0.039719713	0.714830695	yes	down
ENSSSCG00000039930	ZNF446	0.81193869	-0.300557302	0.02592215	0.683249021	yes	down
ENSSSCG00000040003	UAP1L1	0.799858797	-0.322182758	0.021675773	0.67241349	yes	down
ENSSSCG00000040009	—	60.83747814	5.926888446	4.93E-05	0.070265172	yes	up
ENSSSCG00000040035	MUC2	0.502101317	-0.993949585	4.86E-05	0.070265172	yes	down
ENSSSCG00000040100	ITPK	0.743712913	-0.427182273	0.015947118	0.67109181	yes	down
ENSSSCG00000040107	GMEB2	0.751033532	-0.413050773	0.044639382	0.72213448	yes	down
ENSSSCG00000040139	B3GNT9	0.810813791	-0.302557467	0.016217889	0.67109181	yes	down
ENSSSCG00000040503	LRRC45	0.703256992	-0.507876104	0.014690003	0.652664121	yes	down
ENSSSCG00000040533	ACTMAP	0.716257192	-0.481450376	0.03189213	0.703664106	yes	down
ENSSSCG00000040624	REXO1	0.77630133	-0.365311335	0.015089652	0.656815274	yes	down
ENSSSCG00000040631	LPL	0.65364579	-0.613419043	0.006483781	0.527035893	yes	down
ENSSSCG00000040673	TMEM140	1.526396206	0.610129491	0.032260927	0.703664106	yes	up
ENSSSCG00000040690	ZBED6	0.661177324	-0.596890849	0.002022672	0.353910414	yes	down
ENSSSCG00000040713	TXLN	1.22283355	0.29022804	0.037494041	0.707740306	yes	up
ENSSSCG00000040793	CTSD	0.728441297	-0.457115381	0.006804702	0.528701837	yes	down
ENSSSCG00000040795	UBE1	0.120663239	-3.050941882	0.035054044	0.707740306	yes	down
ENSSSCG00000040869	INPP5E	0.677869244	-0.56092108	0.034097242	0.704233345	yes	down
ENSSSCG00000040875	ZFPM1	0.658602974	-0.602519069	0.027643147	0.693082926	yes	down
ENSSSCG00000040885	LY6G6E	6.337225361	2.663851321	0.037137004	0.707740306	yes	up
ENSSSCG00000040944	SKI	0.808011581	-0.307552124	0.046914758	0.72213448	yes	down
ENSSSCG00000040968	DUS3L	0.778225562	-0.361739727	0.012055414	0.628850139	yes	down
ENSSSCG00000040989	GPRC5C	0.586910986	-0.768786381	0.01310405	0.635895234	yes	down
ENSSSCG00000040998	LEAP2	1.352777706	0.435924789	0.012305073	0.631103805	yes	up
ENSSSCG00000041169	—	0.40831627	-1.292241038	0.047546486	0.72213448	yes	down
ENSSSCG00000041198	PARK2	1.616057487	0.692478519	0.032614149	0.703664106	yes	up
ENSSSCG00000041214	—	1.984152186	0.988522685	0.046774982	0.72213448	yes	up
ENSSSCG00000041232	MRGPRX	9.427026093	3.236802721	0.012092797	0.628850139	yes	up
ENSSSCG00000041773	KRAB	1.83103877	0.872662339	0.028857243	0.696293501	yes	up
ENSSSCG00000042341	DEPDC7	0.447980464	-1.158492276	0.041067874	0.715680171	yes	down
ENSSSCG00000042594	KRAB	1.773012455	0.826202671	0.01778801	0.67109181	yes	up
ENSSSCG00000042700	KRAB	0.43445987	-1.202705168	0.030526821	0.700958898	yes	down
ENSSSCG00000042889	—	0.816515971	-0.29244699	0.014739873	0.652664121	yes	down
ENSSSCG00000043340	—	10.0117849	3.323627295	0.011942611	1	yes	up
ENSSSCG00000043418	KRAB	0.509723494	-0.972213244	0.033513612	0.703664106	yes	down
ENSSSCG00000043560	INTS3	0.515322179	-0.956453408	0.036636194	0.707740306	yes	down
ENSSSCG00000043977	COX6B	1.342029015	0.424415863	0.025637547	0.682097083	yes	up

ENSSSCG00000044105	STARD9	0.555866134	-0.847190605	0.034806494	0.707740306	yes	down
ENSSSCG00000044127	STARD9	0.422845303	-1.241798142	0.043623699	0.72213448	yes	down
ENSSSCG00000044404	MED18	1.230702616	0.299482194	0.025557932	0.681067485	yes	up
ENSSSCG00000044544	—	9.166503547	3.19637154	0.022897205	1	yes	up
ENSSSCG00000044675	ERV3-1	1.693516681	0.760022198	0.003463426	0.447112352	yes	up
ENSSSCG00000044700	COL4A1	0.705086373	-0.504128097	0.01247744	0.632336999	yes	down
ENSSSCG00000044967	MRGPRX	0.088605679	-3.496457019	0.038910385	1	yes	down
ENSSSCG00000045079	PARK2	2.19267417	1.132691445	0.001570954	0.335087945	yes	up
ENSSSCG00000045134	FASTKD5	1.225572337	0.293455638	0.008348518	0.578565651	yes	up
ENSSSCG00000045152	Cullin 7	0.591363755	-0.757882273	0.027959891	0.693082926	yes	down
ENSSSCG00000045225	—	2.691570621	1.428448279	0.041844539	0.717656392	yes	up
ENSSSCG00000045267	—	0.399623964	-1.323284996	0.001089637	0.310915441	yes	down
ENSSSCG00000045344	—	0.261325519	-1.936080081	0.046309461	0.72213448	yes	down
ENSSSCG00000045401	NEK1_4_5	2.979649998	1.575142876	0.028024194	0.693082926	yes	up
ENSSSCG00000045423	—	2.135294945	1.094435361	0.0010413	0.310915441	yes	up
ENSSSCG00000045657	—	0.530466033	-0.914667721	0.039608778	0.714830695	yes	down
ENSSSCG00000045950	C1orf54	1.388023472	0.473031964	0.017330711	0.67109181	yes	up
ENSSSCG00000046151	—	1.585153175	0.664622257	0.006669931	0.527035893	yes	up
ENSSSCG00000046391	—	1.853980198	0.890625835	0.020938871	0.67109181	yes	up
ENSSSCG00000046490	CTSG	1.74982266	0.807208716	0.049482683	0.725498277	yes	up
ENSSSCG00000046520	MRGPRX	4.976927198	2.315255284	0.013137468	0.635895234	yes	up
ENSSSCG00000046665	PARK2	0.076256436	-3.712997086	0.011990567	0.628217657	yes	down
ENSSSCG00000046754	KRAB	0.368382189	-1.440724783	0.000171495	0.133112815	yes	down
ENSSSCG00000047413	OTOS	0.680126884	-0.556124176	0.033692114	0.703664106	yes	down
ENSSSCG00000047662	—	2.252438668	1.171487823	0.029042591	0.696976005	yes	up
ENSSSCG00000047897	CTSG	1.633940586	0.708355525	0.008265509	0.578565651	yes	up
ENSSSCG00000048001	—	7.22520139	2.853037801	0.005832212	0.523737709	yes	up
ENSSSCG00000048143	—	2.941667341	1.556634108	0.036769827	0.707740306	yes	up
ENSSSCG00000048201	—	1.598332531	0.67656759	0.048061619	0.72213448	yes	up
ENSSSCG00000048309	CCNB1IP1	2.669290762	1.416456465	0.022320471	0.673830277	yes	up
ENSSSCG00000048341	—	0.134700448	-2.892173446	0.012883607	0.635895234	yes	down
ENSSSCG00000048379	—	2.411673572	1.270034647	0.029385381	0.696976005	yes	up
ENSSSCG00000048411	—	0.226822177	-2.140366394	0.004232361	0.475451096	yes	down
ENSSSCG00000048443	APLN	0.693767721	-0.527475378	0.03576789	0.707740306	yes	down
ENSSSCG00000048650	—	0.088574917	-3.496957982	0.025165739	0.679826869	yes	down
ENSSSCG00000049122	—	0.507871661	-0.977464121	0.014488826	0.652664121	yes	down
ENSSSCG00000049239	KRAB	0.355713872	-1.491210859	0.021710826	0.67241349	yes	down
ENSSSCG00000049512	MRGPRX	3.118349858	1.640782798	0.03515526	0.707740306	yes	up
ENSSSCG00000049545	MRGPRX	2.024470578	1.017544676	0.021602916	0.67241349	yes	up
ENSSSCG00000050031	SLC2A6	0.193267636	-2.371328023	0.016909645	0.67109181	yes	down
ENSSSCG00000050072	NEK1_4_5	7.017903649	2.811040141	0.019593637	0.67109181	yes	up

ENSSSCG00000050213	—	1.582192717	0.661925336	0.041280701	0.715680171	yes	up
ENSSSCG00000050754	MRGPRX	1.583647807	0.663251525	0.043175845	0.72213448	yes	up
ENSSSCG00000050997	KRAB	2.611770129	1.385027926	0.00688105	0.531761788	yes	up
ENSSSCG00000050998	DDIT3	0.44445325	-1.169896419	0.023011017	0.675846472	yes	down
ENSSSCG00000051231	—	0.334830013	-1.578499241	0.007300933	0.551414975	yes	down
ENSSSCG00000051266	—	0.384436583	-1.379182466	0.006562038	0.527035893	yes	down
ENSSSCG00000051290	—	1.451844861	0.5378873	0.046292477	0.72213448	yes	up
ENSSSCG00000051557	—	1.725528004	0.787037888	0.045692474	0.72213448	yes	up
ENSSSCG00000051618	SETD1B	0.810208733	-0.30363446	0.032571628	0.703664106	yes	down
ENSSSCG00000051767	—	1.665592217	0.736035232	0.021025716	0.67109181	yes	up
ENSSSCG00000051779	NEK1_4_5	2.584109363	1.369667128	0.010872645	0.617982236	yes	up
ENSSSCG00000051803	—	5.008733947	2.324445981	0.024891522	0.67891626	yes	up
ENSSSCG00000051841	—	0.340933895	-1.552436058	0.005387567	0.515068066	yes	down
ENSSSCG00000051889	—	0.219755559	-2.186028436	0.034962572	0.707740306	yes	down
ENSSSCG00000051899	B2M	1.372735565	0.457053741	0.046188349	0.72213448	yes	up
ENSSSCG00000051948	—	0.108619345	-3.20264702	0.005114961	0.513485708	yes	down
ENSSSCG00000052003	FSCN2	0.623552	-0.681418218	0.000709329	0.268488108	yes	down
ENSSSCG00000052117	TUBA	1.810663028	0.85651808	0.000572414	0.241964073	yes	up
ENSSSCG00000052122	MUC2	0.524966618	-0.929702409	0.006448155	0.527035893	yes	down
ENSSSCG00000052206	—	2.333240299	1.222334897	0.047826826	0.72213448	yes	up
ENSSSCG00000052227	RBIS	1.28413027	0.360791566	0.025337276	0.679826869	yes	up
ENSSSCG00000052265	—	76.16698482	6.251093881	0.039193763	0.714830695	yes	up
ENSSSCG00000052272	—	0.107433556	-3.218483415	0.043672716	1	yes	down
ENSSSCG00000052292	—	1.627919807	0.703029632	0.035215096	0.707740306	yes	up
ENSSSCG00000052434	ZBTB42	0.713926161	-0.486153226	0.048804559	0.722589262	yes	down
ENSSSCG00000052506	—	13.78986519	3.785536448	0.008128281	0.578565651	yes	up
ENSSSCG00000052881	—	0.069482901	-3.847198205	0.023171759	1	yes	down
ENSSSCG00000053091	MANSC4	0.719568987	-0.474795087	0.019523184	0.67109181	yes	down
ENSSSCG00000053118	—	30.49859982	4.930671105	0.003499869	0.447112352	yes	up
ENSSSCG00000053144	CCNB1IP1	0.527964957	-0.921485918	0.045095509	0.72213448	yes	down
ENSSSCG00000053150	CTSG	2.142735829	1.099453996	0.045761794	0.72213448	yes	up
ENSSSCG00000053180	NDUFAF2	1.26576953	0.340014744	0.028493479	0.695002653	yes	up
ENSSSCG00000053185	GNG5	1.274041413	0.349412173	0.001906864	0.34673138	yes	up
ENSSSCG00000053273	—	2.695677095	1.430647692	0.048549499	0.72213448	yes	up
ENSSSCG00000053317	IFT25	1.252799716	0.325155791	0.033615011	0.703664106	yes	up
ENSSSCG00000053358	RAB40B	0.628813685	-0.669295479	0.034830404	0.707740306	yes	down
ENSSSCG00000053471	—	0.075683957	-3.723868677	0.009888611	0.603302839	yes	down
ENSSSCG00000053520	RGS21	1.650104924	0.722557763	0.030184019	0.700714589	yes	up
ENSSSCG00000053533	NPG3	13.81794053	3.788470703	0.024140213	1	yes	up
ENSSSCG00000053758	CTSG	2.943868258	1.55771311	0.007198454	0.547170992	yes	up
ENSSSCG00000053773	—	2.523792397	1.335593241	0.001653447	0.335087945	yes	up

ENSSSCG00000053814	—	14.7816755	3.885737903	0.000281478	0.178126396	yes	up
ENSSSCG00000053860	KRAB	0.333073187	-1.586088877	0.023634421	0.675846472	yes	down
ENSSSCG00000053885	—	1.491426234	0.576692623	0.045741608	0.72213448	yes	up
ENSSSCG00000054202	SEC61G	1.361266369	0.444949397	0.031109193	0.703664106	yes	up
ENSSSCG00000054435	—	0.109538545	-3.190489479	0.004234835	0.475451096	yes	down
ENSSSCG00000054570	—	0.407818917	-1.293999397	0.045660911	0.72213448	yes	down
ENSSSCG00000054571	CYP2B6B	2.462047188	1.299858413	0.047155242	0.72213448	yes	up
ENSSSCG00000054605	CTSG	10.86294001	3.441342711	6.18E-06	0.038196534	yes	up
ENSSSCG00000054616	—	14.93023821	3.900165278	0.018902536	1	yes	up
ENSSSCG00000054784	—	0.497308332	-1.007787492	0.043266143	0.72213448	yes	down
ENSSSCG00000054796	LRRC31	0.623837636	-0.680757502	0.020778252	0.67109181	yes	down
ENSSSCG00000054912	—	0.135778019	-2.880678148	0.028869882	0.696293501	yes	down
ENSSSCG00000054940	—	5.353748149	2.420549273	0.043403916	0.72213448	yes	up
ENSSSCG00000054999	ZBTB21	0.603450535	-0.728692578	0.001681516	0.335087945	yes	down
ENSSSCG00000055161	NKD2	0.725683717	-0.462587195	0.035263939	0.707740306	yes	down
ENSSSCG00000055296	—	0.252691039	-1.984553592	0.004083196	0.475451096	yes	down
ENSSSCG00000055375	AHNAK	0.671991893	-0.573484268	0.015294692	0.657271396	yes	down
ENSSSCG00000055439	KRAB	0.319597249	-1.645673107	0.048234083	0.72213448	yes	down
ENSSSCG00000055487	COMMD7	1.216135336	0.282303786	0.002425005	0.379220559	yes	up
ENSSSCG00000055506	—	0.205049262	-2.285957544	0.040372877	0.715680171	yes	down
ENSSSCG00000055583	—	0.542562018	-0.882140038	0.005622664	0.523737709	yes	down
ENSSSCG00000055672	—	0.739120222	-0.436119048	0.020057376	0.67109181	yes	down
ENSSSCG00000055775	—	3.844772379	1.942898189	0.022488863	0.673830277	yes	up
ENSSSCG00000056050	CLCN1	0.453936848	-1.139436493	0.046418393	0.72213448	yes	down
ENSSSCG00000056124	—	1.901002897	0.926760731	0.042601031	0.72213448	yes	up
ENSSSCG00000056134	—	0.272387233	-1.87626901	0.047745013	0.72213448	yes	down
ENSSSCG00000056309	SOX4	0.540888613	-0.886596569	0.005898635	0.523737709	yes	down
ENSSSCG00000056445	—	4.679774655	2.226439061	0.042691688	0.72213448	yes	up
ENSSSCG00000056548	CTSG	2.126107015	1.088214215	0.001073658	0.310915441	yes	up
ENSSSCG00000056654	—	13.13071565	3.714873643	2.67E-07	0.002472715	yes	up
ENSSSCG00000056668	UFM1	1.244336091	0.315376205	0.021971872	0.673830277	yes	up
ENSSSCG00000056939	—	1.435782459	0.521837178	0.046459119	0.72213448	yes	up
ENSSSCG00000056986	—	5.935031399	2.569255662	0.026867092	0.690849599	yes	up
ENSSSCG00000057179	—	1.277476662	0.353296935	0.048486626	0.72213448	yes	up
ENSSSCG00000057247	TRIM52	1.401397177	0.486865895	0.025474722	0.679826869	yes	up
ENSSSCG00000057276	RPL27A	2.29466931	1.198286258	0.027850739	0.693082926	yes	up
ENSSSCG00000057336	—	0.323448517	-1.628391995	0.019768071	0.67109181	yes	down
ENSSSCG00000057380	—	2.892909967	1.532521424	0.009459343	0.602894951	yes	up
ENSSSCG00000057461	CCNB1IP1	0.079978678	-3.64424076	0.041793494	1	yes	down
ENSSSCG00000057484	—	2.481325467	1.311110981	0.004767902	0.508219993	yes	up
ENSSSCG00000057510	PLA2G15	0.801673283	-0.3189137	0.040685397	0.715680171	yes	down

ENSSSCG00000057514	—	0.591034488	-0.758685777	0.02154631	0.67241349	yes	down
ENSSSCG00000057599	—	0.125942647	-2.989161198	0.039824163	0.714830695	yes	down
ENSSSCG00000058009	SIGLEC5	2.088068182	1.062168821	0.010667109	0.617982236	yes	up
ENSSSCG00000058187	—	1.725777398	0.787246388	0.025439309	0.679826869	yes	up
ENSSSCG00000058216	IGFBP3	0.678026887	-0.56058561	0.037017279	0.707740306	yes	down
ENSSSCG00000058218	—	0.538714197	-0.892408009	0.020128329	0.67109181	yes	down
ENSSSCG00000058286	—	0.825915846	-0.275933305	0.037291001	0.707740306	yes	down
ENSSSCG00000058312	—	0.597604555	-0.74273695	0.041313664	0.715680171	yes	down
ENSSSCG00000058337	—	3.347742113	1.743188397	0.037086428	0.707740306	yes	up
ENSSSCG00000058372	—	5.835505822	2.544857714	0.044141544	0.72213448	yes	up
ENSSSCG00000058492	ARSD	0.689494465	-0.536389125	0.0447955	0.72213448	yes	down
ENSSSCG00000058502	CYP2J	1.547767801	0.630189052	0.006076857	0.527035893	yes	up
ENSSSCG00000058509	—	0.098165437	-3.348641033	0.021614235	1	yes	down
ENSSSCG00000058585	—	2.254801368	1.173000348	0.016924738	0.67109181	yes	up
ENSSSCG00000058592	—	0.192679843	-2.375722443	0.022728558	0.675846472	yes	down
ENSSSCG00000058619	GBP6	1.588445334	0.667615441	0.04383357	0.72213448	yes	up
ENSSSCG00000058882	—	0.135127442	-2.887607409	0.037813054	0.709061263	yes	down
ENSSSCG00000058958	—	14.46284677	3.854279646	0.001862727	0.342059367	yes	up
ENSSSCG00000059011	—	3.215501006	1.685043541	0.002012931	0.353910414	yes	up
ENSSSCG00000059088	—	20.33089362	4.345601723	0.020673098	0.67109181	yes	up
ENSSSCG00000059119	CHST2	0.700750757	-0.513026697	0.001275717	0.313270149	yes	down
ENSSSCG00000059123	CTU1	0.696273129	-0.522274748	0.025348879	0.679826869	yes	down
ENSSSCG00000059202	CCDC71L	0.53473316	-0.90310895	0.00391209	0.474147655	yes	down
ENSSSCG00000059227	FOXQ	0.184690821	-2.436815926	0.006369006	0.527035893	yes	down
ENSSSCG00000059250	—	0.127238936	-2.974387879	0.046237718	0.72213448	yes	down
ENSSSCG00000059300	—	0.46656656	-1.099845186	0.003274358	0.445536806	yes	down
ENSSSCG00000059328	—	1.718455529	0.781112517	0.016784639	0.67109181	yes	up
ENSSSCG00000059382	—	11.4347131	3.515348264	0.044674816	1	yes	up
ENSSSCG00000059531	—	0.433513709	-1.205850478	0.042975321	0.72213448	yes	down
ENSSSCG00000059711	—	1.273619726	0.348934586	0.031812875	0.703664106	yes	up
ENSSSCG00000059725	NEK1_4_5	0.764820236	-0.386807399	0.041869195	0.717656392	yes	down
ENSSSCG00000059880	GZMB	2.035873013	1.025647577	0.019276244	0.67109181	yes	up
ENSSSCG00000059892	—	3.576490188	1.838544483	0.023385461	0.675846472	yes	up
ENSSSCG00000059920	MEGF8	0.766251129	-0.384110801	0.02077194	0.67109181	yes	down
ENSSSCG00000059930	CLCA3_4	3.402441103	1.766570189	0.006631665	0.527035893	yes	up
ENSSSCG00000060060	KCNA6	0.543408225	-0.879891693	0.011329885	0.617982236	yes	down
ENSSSCG00000060069	ZNF74	0.802542597	-0.317350125	0.032027049	0.703664106	yes	down
ENSSSCG00000060173	—	1.444282138	0.530352597	0.014843119	0.6537276	yes	up
ENSSSCG00000060176	—	3.255133392	1.702716665	0.027004415	0.690849599	yes	up
ENSSSCG00000060482	—	0.665656539	-0.587150117	0.016937596	0.67109181	yes	down
ENSSSCG00000060547	—	7.053673373	2.818374772	0.042618411	0.72213448	yes	up

ENSSSCG00000060584	IGH	0.489940539	-1.029321425	0.006677815	0.527035893	yes	down
ENSSSCG00000060596	—	3.658142719	1.871111361	0.018812907	0.67109181	yes	up
ENSSSCG00000060736	FOXD	0.76312414	-0.39001033	0.01461616	0.652664121	yes	down
ENSSSCG00000060781	—	0.663191345	-0.592502917	0.038539172	0.71278147	yes	down
ENSSSCG00000060807	HYAL1	0.747513117	-0.4198292	0.033141366	0.703664106	yes	down
ENSSSCG00000060830	—	0.316432295	-1.660031245	0.033935012	0.703664106	yes	down
ENSSSCG00000060858	SELENOK	1.237227311	0.307110585	0.014565436	0.652664121	yes	up
ENSSSCG00000060872	—	4.649939902	2.21721207	0.004384231	0.481149946	yes	up
ENSSSCG00000061180	—	0.702868392	-0.508673516	0.0412244	0.715680171	yes	down
ENSSSCG00000061194	RBM33	0.830746295	-0.267520141	0.037858417	0.709061263	yes	down
ENSSSCG00000061433	PITX1	0.688490555	-0.538491231	0.003703353	0.458265629	yes	down
ENSSSCG00000061450	MHC2	1.794958423	0.843950427	0.043283154	0.72213448	yes	up
ENSSSCG00000061491	—	0.535247188	-0.901722784	0.003999567	0.475451096	yes	down
ENSSSCG00000061553	—	15.82107688	3.983775896	0.038918033	1	yes	up
ENSSSCG00000061672	—	10.28898216	3.363028365	0.000477632	0.239136655	yes	up
ENSSSCG00000061676	CYP2J	0.615196733	-0.700880254	0.021095965	0.67109181	yes	down
ENSSSCG00000062085	—	0.677671746	-0.561341473	0.047021119	0.72213448	yes	down
ENSSSCG00000062439	—	0.23573628	-2.08475429	0.011578243	0.623024075	yes	down
ENSSSCG00000062461	—	0.120223457	-3.056209681	0.026652718	1	yes	down
ENSSSCG00000062489	—	0.094948731	-3.396707466	1.51E-07	0.002472715	yes	down
ENSSSCG00000062538	DEXI	0.72857118	-0.456858168	0.043089256	0.72213448	yes	down
ENSSSCG00000062590	GPR31	1.735840639	0.795634505	0.040172023	0.715038878	yes	up
ENSSSCG00000062610	—	0.707559965	-0.499075674	0.000419831	0.229108458	yes	down
ENSSSCG00000062656	GBP5	2.170273561	1.117876904	0.049263007	0.724249616	yes	up
ENSSSCG00000062748	—	0.061394414	-4.025748781	0.014928672	0.6537276	yes	down
ENSSSCG00000062762	FIGNL2	0.447790218	-1.159105081	0.013995908	0.651736454	yes	down
ENSSSCG00000062764	—	1.255654023	0.328439005	0.048766545	0.722589262	yes	up
ENSSSCG00000062887	—	0.259527544	-1.946040436	0.028222658	0.695002653	yes	down
ENSSSCG00000062928	RHOT2	0.802684431	-0.317095179	0.046488059	0.72213448	yes	down
ENSSSCG00000063043	INAFM2	0.477156904	-1.067464347	0.040711347	0.715680171	yes	down
ENSSSCG00000063100	—	29.88265172	4.90123627	0.001669286	0.335087945	yes	up
ENSSSCG00000063102	KIF26	0.201747682	-2.309376	0.018787853	0.67109181	yes	down
ENSSSCG00000063299	SLC7A3	0.32638497	-1.61535347	0.039095279	0.714830695	yes	down
ENSSSCG00000063327	PARK2	1.663216819	0.733976253	0.015394098	0.657867138	yes	up
ENSSSCG00000063342	GBP2	2.025581795	1.018336344	0.021349931	0.67241349	yes	up
ENSSSCG00000063462	TMEM127	0.627787187	-0.671652511	0.04895071	0.722841414	yes	down
ENSSSCG00000063463	GPR146	0.620053706	-0.689534915	0.020999001	0.67109181	yes	down
ENSSSCG00000063553	—	0.585415774	-0.772466476	0.008391339	0.578565651	yes	down

Table S9. List of differentially expressed genes (DEGs) between the LBW-bile powder and LBW-CON groups.

Gene ID	Gene Name	FC	Log2FC	Pvalue	Padjust	Significant	Regulate
ENSSSCG00000000006	PPARA	1.406462397	0.492070981	0.006744703	0.07467161	yes	up
ENSSSCG00000000061	PMM1	0.75878399	-0.398238856	0.029336721	0.593091889	yes	down
ENSSSCG00000000080	GRAP2	0.608092483	-0.717637339	0.021248059	0.51572868	yes	down
ENSSSCG00000000108	TMEM184B	0.823906731	-0.279447066	0.021989162	0.52018032	yes	down
ENSSSCG00000000130	CYTH4	0.720124663	-0.473681418	0.004706055	0.333646722	yes	down
ENSSSCG00000000131	ELFN2	7.557467794	2.917902926	0.01079821	1	yes	up
ENSSSCG00000000135	KCTD17	0.506287974	-0.981969879	0.000810935	0.153884078	yes	down
ENSSSCG00000000136	CSF2RB	0.483024316	-1.049832277	0.000147116	0.068714733	yes	down
ENSSSCG00000000137	NCF4	0.594378068	-0.750547212	0.005205337	0.346310468	yes	down
ENSSSCG00000000142	FOXRED2	0.759387042	-0.397092713	0.034153161	0.627268647	yes	down
ENSSSCG00000000205	FAM186B	0.409596224	-1.28772568	0.029831063	0.598270325	yes	down
ENSSSCG00000000220	POU6F1	1.505353093	0.590101923	0.033807275	0.626076307	yes	up
ENSSSCG00000000223	BIN2	0.602891428	-0.730029878	0.000836123	0.154772348	yes	down
ENSSSCG00000000232	ACVRL1	0.78252832	-0.353785129	0.016187667	0.483087156	yes	down
ENSSSCG00000000257	ITGB7	0.676597075	-0.563631155	0.018419686	0.499624348	yes	down
ENSSSCG00000000291	GPR84	0.34248048	-1.545906333	0.014086367	0.456766144	yes	down
ENSSSCG00000000292	ZNF385A	0.696801253	-0.521180876	0.013109567	0.448599721	yes	down
ENSSSCG00000000295	NCKAP1L	0.618176437	-0.693909431	0.005758189	0.361488691	yes	down
ENSSSCG00000000370	DGKA	0.759557921	-0.396768112	0.007742981	0.387495665	yes	down
ENSSSCG00000000437	ARHGAP9	0.649353681	-0.622923616	0.010435742	0.419919165	yes	down
ENSSSCG00000000464	C12orf56	0.144090748	-2.794950396	0.032589428	1	yes	down
ENSSSCG00000000475	IRAK3	0.6803842	-0.555578455	0.009088031	0.396883703	yes	down
ENSSSCG00000000487	SLC35E3	1.256573604	0.32949518	0.012230517	0.442351644	yes	up
ENSSSCG00000000547	MRPS35	1.310757096	0.390400356	0.030865476	0.607123214	yes	up
ENSSSCG00000000651	CLEC12B	0.089324599	-3.484798664	0.023194995	1	yes	down
ENSSSCG00000000653	CD69	0.651434477	-0.618308019	0.006046156	0.366967563	yes	down
ENSSSCG00000000656	CLEC2B	0.704625266	-0.505071889	0.033967611	0.626076307	yes	down
ENSSSCG00000000664	A2ML1	2.121829658	1.08530884	0.02238282	0.524686071	yes	up
ENSSSCG00000000688	LAG3	0.535657968	-0.900616001	0.009094799	0.396883703	yes	down
ENSSSCG00000000707	SCNN1A	0.631715133	-0.662653963	0.021109533	0.513921244	yes	down
ENSSSCG00000000712	VWF	0.717399724	-0.479150904	0.002050434	0.232926459	yes	down
ENSSSCG00000000734	TSPAN11	0.77105688	-0.375090805	0.041811286	0.667913669	yes	down
ENSSSCG00000000811	PCED1B	0.554637148	-0.850383848	0.000716611	0.145734681	yes	down
ENSSSCG00000000837	CHST11	0.730807503	-0.452436649	0.013732685	0.451421485	yes	down
ENSSSCG00000000866	MYBPC1	0.129868352	-2.9448782	0.044001561	1	yes	down
ENSSSCG00000000907	PLXNC1	0.577065448	-0.793193142	0.012022707	0.439315508	yes	down
ENSSSCG00000000915	DCN	0.800891269	-0.320321702	0.000989316	0.172248434	yes	down
ENSSSCG00000000916	LUM	0.758185434	-0.399377355	0.012747271	0.447694574	yes	down

ENSSSCG00000000997	PPP1R3G	2.559935964	1.356107722	0.035773396	0.633787122	yes	up
ENSSSCG00000001052	PHACTR1	0.648779055	-0.624200851	0.015171742	0.467600207	yes	down
ENSSSCG00000001064	GMPR	0.759917912	-0.396084512	0.033633671	0.626076307	yes	down
ENSSSCG00000001073	TPMT	1.344911032	0.427510739	0.006523125	0.366967563	yes	up
ENSSSCG00000001097	RIPOR2	0.658883601	-0.601904475	0.017795555	0.492991747	yes	down
ENSSSCG00000001203	ZSCAN9	0.397312502	-1.331653905	0.03538079	0.633787122	yes	down
ENSSSCG00000001422	C2	0.706134057	-0.501985995	0.016809626	0.485997014	yes	down
ENSSSCG00000001439	GPSM3	0.761936648	-0.392257047	0.049364674	0.696771975	yes	down
ENSSSCG00000001453	HLA-DRA	0.567863247	-0.816384554	0.007955609	0.387495665	yes	down
ENSSSCG00000001455	MHC2	0.548026513	-0.867682405	0.002803656	0.2721991	yes	down
ENSSSCG00000001457	SLA-DQB1	0.540880244	-0.886618892	0.006044066	0.366967563	yes	down
ENSSSCG00000001469	SLA-DMB	0.651592258	-0.617958632	0.016058726	0.480581846	yes	down
ENSSSCG00000001470	SLA-DMA	0.664158078	-0.590401432	0.013457647	0.449667171	yes	down
ENSSSCG00000001479	HMGCLL1	2.617778047	1.388342781	0.002935863	0.280148416	yes	up
ENSSSCG00000001500	COL21A1	0.696013522	-0.522812759	0.017747178	0.492991747	yes	down
ENSSSCG00000001660	PTK7	0.79346065	-0.333769418	0.046042558	0.687457963	yes	down
ENSSSCG00000001703	NFKBIE	0.807732878	-0.308049831	0.026514817	0.569054167	yes	down
ENSSSCG00000001720	SLC25A27	2.051121558	1.036412994	7.07E-06	0.011201019	yes	up
ENSSSCG00000001723	PLA2G7	0.565540072	-0.822298842	0.000218196	0.082219172	yes	down
ENSSSCG00000001750	PAQR8	0.738497729	-0.437334611	0.013053988	0.448536088	yes	down
ENSSSCG00000001787	IL16	0.600433342	-0.735924003	0.001625424	0.209336848	yes	down
ENSSSCG00000001849	ANPEP	0.364620545	-1.455532242	0.03899668	0.657025111	yes	down
ENSSSCG00000001887	SCAMP5	0.679059576	-0.558389942	0.000894547	0.16062405	yes	down
ENSSSCG00000002032	SLC7A8	0.507375968	-0.978872908	0.008630492	0.389694612	yes	down
ENSSSCG00000002260	ARHGEF13	1.817014608	0.861570018	0.008460204	0.389033038	yes	up
ENSSSCG00000002263	SLCO3A1	0.709954082	-0.494202376	0.000176823	0.070304103	yes	down
ENSSSCG00000002268	AKAP5	0.553527549	-0.853272974	0.025044608	0.554668316	yes	down
ENSSSCG00000002408	ADCK1	1.326749564	0.407896075	0.020146483	0.508965388	yes	up
ENSSSCG00000002432	KCNK13	0.271264621	-1.882227195	0.019800742	0.504766715	yes	down
ENSSSCG00000002451	RIN3	0.680183467	-0.556004155	0.001790893	0.215227738	yes	down
ENSSSCG00000002452	LGMN	0.670753821	-0.576144726	0.003917918	0.308609991	yes	down
ENSSSCG00000002479	SERPINA11	5.537609206	2.469263245	0.001670651	0.211347945	yes	up
ENSSSCG00000002515	SLC25A47	0.603738595	-0.728004065	0.02713092	0.571323123	yes	down
ENSSSCG00000002520	SLC25A20_29	0.657495388	-0.604947319	0.01472541	0.46161769	yes	down
ENSSSCG00000002554	PLD4	0.651195259	-0.618837899	0.026903451	0.571323123	yes	down
ENSSSCG00000002620	EFHC1	0.583230839	-0.77786109	0.017890472	0.492991747	yes	down
ENSSSCG00000002672	MEAK7	1.232203796	0.301240885	0.041280854	0.667123021	yes	up
ENSSSCG00000002712	LDHD	1.959218749	0.970278485	0.000755529	0.148430406	yes	up
ENSSSCG00000002714	WDR59	1.211663755	0.276989397	0.036857325	0.642463958	yes	up
ENSSSCG00000002746	DHODH	1.304325401	0.383303836	0.003174824	0.288459657	yes	up
ENSSSCG00000002825	CES1	0.484966525	-1.044042926	0.000715228	0.145734681	yes	down

ENSSSCG00000002829	MMP2	0.770000948	-0.377067874	0.033938067	0.626076307	yes	down
ENSSSCG00000002879	GRAMD1A	0.807881206	-0.307784927	0.028855564	0.587633011	yes	down
ENSSSCG00000002893	CD22	0.705329647	-0.503630414	0.04705149	0.691288485	yes	down
ENSSSCG00000002916	APLP1	0.529727606	-0.916677401	0.005698265	0.359764441	yes	down
ENSSSCG00000002919	TYROBP	0.666927338	-0.584398507	0.011228244	0.434053184	yes	down
ENSSSCG00000002957	GGN	0.62744569	-0.672437506	0.034245105	0.627268647	yes	down
ENSSSCG00000002960	RASGRP4	0.621383824	-0.686443411	0.021142817	0.513921244	yes	down
ENSSSCG00000002962	MAP4K1	0.643555061	-0.635864509	0.030449418	0.603889357	yes	down
ENSSSCG00000003048	CXCL17	0.501926361	-0.994452377	0.015421203	0.468837737	yes	down
ENSSSCG00000003090	PVRL2	0.802065394	-0.318208227	0.001034899	0.178162665	yes	down
ENSSSCG00000003146	NTN5	1.635670185	0.709881874	0.003487747	0.296453856	yes	up
ENSSSCG00000003170	SLC17A7	0.490059577	-1.028970946	0.013984837	0.456766144	yes	down
ENSSSCG00000003192	IL4I1	0.505253958	-0.984919376	0.011137572	0.434053184	yes	down
ENSSSCG00000003231	NKG7	0.607182306	-0.719798345	0.008894641	0.394634056	yes	down
ENSSSCG00000003253	—	0.683326913	-0.549352147	0.012908918	0.448536088	yes	down
ENSSSCG00000003331	TNFRSF4	0.383752509	-1.381751913	0.00073644	0.146402578	yes	down
ENSSSCG00000003334	UBE2J2	0.818650394	-0.288680617	0.047446678	0.691288485	yes	down
ENSSSCG00000003395	PIK3CD	0.614797601	-0.701816559	0.032094066	0.614363628	yes	down
ENSSSCG00000003400	NMNAT1	1.368651255	0.452754881	0.001116828	0.184652595	yes	up
ENSSSCG00000003439	DHRS3	0.729716619	-0.454591785	7.38E-06	0.011201019	yes	down
ENSSSCG00000003451	PDPN	0.503652695	-0.989498861	0.008353388	0.389033038	yes	down
ENSSSCG00000003458	EFHD2	0.733089161	-0.447939421	0.037599	0.647166387	yes	down
ENSSSCG00000003486	ARHGEF10L	1.230241611	0.298941679	0.013462833	0.449667171	yes	up
ENSSSCG00000003514	HSPG2	0.756922626	-0.401782261	0.022433114	0.524686071	yes	down
ENSSSCG00000003524	C1QA	0.534458445	-0.903850313	0.004535707	0.326473146	yes	down
ENSSSCG00000003582	SMPDL3B	0.692217027	-0.530703665	0.005123956	0.346159768	yes	down
ENSSSCG00000003584	THEMIS2	0.553505223	-0.853331164	0.00016209	0.068714733	yes	down
ENSSSCG00000003589	MECR	1.226010839	0.293971734	0.008547473	0.389033038	yes	up
ENSSSCG00000003608	LCK	0.663215989	-0.592449306	0.017817222	0.492991747	yes	down
ENSSSCG00000003620	TRIM62	0.67953016	-0.557390511	0.006781423	0.368533767	yes	down
ENSSSCG00000003663	HPCAL1	1.360443202	0.444076726	0.04569654	0.687457963	yes	up
ENSSSCG00000003684	MTCL1	0.633533698	-0.658506735	0.025550333	0.5583584	yes	down
ENSSSCG00000003694	EMILIN2	0.574097456	-0.800632432	0.016477285	0.484426375	yes	down
ENSSSCG00000003708	ANKRD29	1.590647252	0.669613934	0.011212333	0.434053184	yes	up
ENSSSCG00000003715	ZNF521	0.732772034	-0.448563651	0.008649678	0.389694612	yes	down
ENSSSCG00000003776	ACADM	1.263325719	0.337226652	0.040198371	0.665285024	yes	up
ENSSSCG00000003780	CRYZ	1.35859978	0.442120526	0.01930677	0.504239817	yes	up
ENSSSCG00000003783	FPGT	1.204584051	0.268535063	0.045753819	0.687457963	yes	up
ENSSSCG00000003815	ALG6	1.316350709	0.39654391	0.02933276	0.593091889	yes	up
ENSSSCG00000003825	CYP2J	0.768505483	-0.379872542	0.01603776	0.480581846	yes	down
ENSSSCG00000003875	TTC39A	1.486470102	0.571890446	0.001618325	0.209336848	yes	up

ENSSSCG00000003882	SLC5A9	2.620346471	1.389757582	0.02993565	0.598270325	yes	up
ENSSSCG00000003926	BTBD19	0.641239015	-0.641065888	0.020596879	0.511065793	yes	down
ENSSSCG00000003928	PLK3	0.657292045	-0.605393569	0.034049119	0.6268867	yes	down
ENSSSCG00000003989	KRAB	1.377434779	0.461984009	0.004314424	0.323023561	yes	up
ENSSSCG00000004052	FNDC1	0.551700725	-0.858042219	0.000618797	0.145734681	yes	down
ENSSSCG00000004053	TAGAP	0.659378235	-0.600821827	0.020513552	0.511065793	yes	down
ENSSSCG00000004057	SYTL3	0.695428009	-0.52402692	0.016715594	0.48592959	yes	down
ENSSSCG00000004101	LRP11	1.52838119	0.612004407	0.010112275	0.416773906	yes	up
ENSSSCG00000004114	ADGB	0.61554148	-0.700072014	0.043348743	0.678952552	yes	down
ENSSSCG00000004154	TNFAIP3	0.732507967	-0.449083645	0.012961615	0.448536088	yes	down
ENSSSCG00000004156	IFNGR1	0.744204304	-0.426229362	0.001112219	0.184652595	yes	down
ENSSSCG00000004157	IL20RA	0.656170779	-0.607856746	0.001190184	0.192960066	yes	down
ENSSSCG00000004216	ECHDC1	0.64702994	-0.628095623	0.015444501	0.468837737	yes	down
ENSSSCG00000004374	QRSL1	1.262840408	0.33667233	0.021082029	0.513921244	yes	up
ENSSSCG00000004379	SOBP	0.727060256	-0.45985316	0.03746337	0.647166387	yes	down
ENSSSCG00000004485	CD109	0.78783783	-0.344029402	0.046693404	0.691252799	yes	down
ENSSSCG00000004534	CCDC68	1.252732728	0.325078647	0.032464938	0.61746241	yes	up
ENSSSCG00000004547	PLEKHO2	0.713793498	-0.486421335	0.01459757	0.460803078	yes	down
ENSSSCG00000004550	OAZ2	0.788907141	-0.342072598	0.027355804	0.572788782	yes	down
ENSSSCG00000004646	ATP8B4	0.669693553	-0.578427016	0.049111091	0.695594658	yes	down
ENSSSCG00000004676	DUOXA2	0.602527819	-0.730900241	0.030085016	0.598769792	yes	down
ENSSSCG00000004678	DUOX2	0.577336144	-0.792516548	0.000843421	0.154772348	yes	down
ENSSSCG00000004679	SORD	0.760675479	-0.394646995	0.006701265	0.368533767	yes	down
ENSSSCG00000004732	PLA2G4F	1.504962358	0.589727402	0.038204533	0.64849429	yes	up
ENSSSCG00000004758	RHOV	0.653589007	-0.613544376	0.005313258	0.348080505	yes	down
ENSSSCG00000004856	NFATC1	0.643943054	-0.634994983	0.008980071	0.394634056	yes	down
ENSSSCG00000004889	SERPINB10	0.578214092	-0.790324326	0.027519639	0.572788782	yes	down
ENSSSCG00000005067	CCDC198	1.281183074	0.357476643	0.027100606	0.571323123	yes	up
ENSSSCG00000005095	PRKCH	0.810306259	-0.30346081	0.007170168	0.381350169	yes	down
ENSSSCG00000005180	BNC2	1.590167205	0.669178472	0.047968907	0.691288485	yes	up
ENSSSCG00000005311	CD72	0.556634442	-0.845197916	0.00586344	0.362642915	yes	down
ENSSSCG00000005312	SIT1	0.617560614	-0.69534735	0.008461026	0.389033038	yes	down
ENSSSCG00000005314	ARHGEF39	1.253144785	0.32555311	0.015224402	0.467600207	yes	up
ENSSSCG00000005360	IGFBPL1	1.667675443	0.737838544	0.043103445	0.678952552	yes	up
ENSSSCG00000005426	FKTN	1.366646403	0.450640018	0.000236333	0.082219172	yes	up
ENSSSCG00000005494	TNC	0.535318358	-0.901530966	0.008107041	0.387495665	yes	down
ENSSSCG00000005498	PAPPA	1.885091082	0.914634232	0.037357101	0.647166387	yes	up
ENSSSCG00000005503	TLR4	0.65075298	-0.619818082	0.044690725	0.679681619	yes	down
ENSSSCG00000005511	TRAF1	0.779882731	-0.358670889	0.044166308	0.679567425	yes	down
ENSSSCG00000005518	GGTA1	0.737086416	-0.440094324	0.00475525	0.333646722	yes	down
ENSSSCG00000005533	PTGS1	0.661696066	-0.595759392	0.020705557	0.512240136	yes	down

ENSSSCG00000005593	OLFML2A	0.779335465	-0.359683624	0.020116444	0.508965388	yes	down
ENSSSCG00000005620	SH2D3C	0.730558101	-0.45292908	0.017569774	0.492991747	yes	down
ENSSSCG00000005638	LCN2	0.30613283	-1.707770326	0.005311551	0.348080505	yes	down
ENSSSCG00000005650	CERCAM	0.811824944	-0.300759426	0.023910815	0.539576619	yes	down
ENSSSCG00000005665	PHYHD1	0.636484418	-0.651802899	0.044125571	0.679567425	yes	down
ENSSSCG00000005689	FNBP1	0.70036492	-0.513821271	0.001629666	0.209336848	yes	down
ENSSSCG00000005761	PPP1R26	1.26819016	0.342771088	0.041348124	0.667123021	yes	up
ENSSSCG00000005844	NRARP	1.395632615	0.480919218	0.007979571	0.387495665	yes	up
ENSSSCG00000005896	LRRC24	1.803851959	0.851080943	0.004834923	0.33781751	yes	up
ENSSSCG00000005944	NDRG1	1.277295419	0.353092237	0.005010452	0.345450877	yes	up
ENSSSCG00000005948	TG	0.173586169	-2.526276096	0.018260924	1	yes	down
ENSSSCG00000006035	ANGPT1	1.348542027	0.431400483	0.013729163	0.451421485	yes	up
ENSSSCG00000006060	GRHL2	1.250466913	0.322466884	0.000691402	0.145734681	yes	up
ENSSSCG00000006106	CDH17	1.204077614	0.26792839	0.023089399	0.52942603	yes	up
ENSSSCG00000006140	CA2	0.737059656	-0.440146702	0.04040015	0.667123021	yes	down
ENSSSCG00000006141	CA3	2.263735075	1.178705129	0.015329762	0.467825317	yes	up
ENSSSCG00000006163	PKIA	0.806059634	-0.311041519	0.007351892	0.385211341	yes	down
ENSSSCG00000006164	WNT8B	1.847646122	0.885688465	0.035640687	0.633787122	yes	up
ENSSSCG00000006197	SULF1	0.736985464	-0.440291931	0.018547665	0.501177103	yes	down
ENSSSCG00000006233	CA8	1.600338703	0.678377276	0.017872792	0.492991747	yes	up
ENSSSCG00000006287	SELL	0.633950482	-0.65755794	0.044454483	0.679681619	yes	down
ENSSSCG00000006324	ALDH9A1	1.281478959	0.357809791	0.04395257	0.679567425	yes	up
ENSSSCG00000006329	LMX1A	1.85965544	0.895035341	0.03787471	0.647166387	yes	up
ENSSSCG00000006350	FCGR2B	0.57844731	-0.789742542	0.04757058	0.691288485	yes	down
ENSSSCG00000006357	FCER1G	0.572467085	-0.80473535	0.002502697	0.257978607	yes	down
ENSSSCG00000006372	ARHGAP30	0.653403077	-0.613954845	0.01392724	0.456021526	yes	down
ENSSSCG00000006374	CD244	0.601511776	-0.733335113	0.006074739	0.366967563	yes	down
ENSSSCG00000006376	—	0.465596285	-1.102848548	0.001309424	0.198589146	yes	down
ENSSSCG00000006379	CD48	0.691929218	-0.531303633	0.016258158	0.483087156	yes	down
ENSSSCG00000006380	SLAMF1	0.433234197	-1.206780971	0.000658401	0.145734681	yes	down
ENSSSCG00000006382	SLAMF6	0.607986695	-0.717888344	0.041746579	0.667913669	yes	down
ENSSSCG00000006398	SLAMF8	0.5057293	-0.98356273	0.014406641	0.457630538	yes	down
ENSSSCG00000006413	FCER1A	0.597378618	-0.743282495	0.012597435	0.44697551	yes	down
ENSSSCG00000006524	THBS3	0.687850654	-0.539832734	0.020458401	0.511065793	yes	down
ENSSSCG00000006562	GATAD2B	0.801218626	-0.319732135	0.017514158	0.492991747	yes	down
ENSSSCG00000006634	TNFAIP8L2	0.654723943	-0.611041356	0.035589134	0.633787122	yes	down
ENSSSCG00000006643	ANXA9	1.560489195	0.641998368	0.022225492	0.522737311	yes	up
ENSSSCG00000006688	ANKRD35	0.621285267	-0.686672252	0.049071604	0.695594658	yes	down
ENSSSCG00000006690	NUDT17	1.438379053	0.524443916	0.013625462	0.449667171	yes	up
ENSSSCG00000006729	TENT5C	0.676541727	-0.563749177	0.00219549	0.238317669	yes	down
ENSSSCG00000006736	CD2	0.701602733	-0.511273727	0.020964539	0.513921244	yes	down

ENSSSCG00000006750	SYCP1	0.350750061	-1.511484738	0.003412585	0.296453856	yes	down
ENSSSCG00000006788	ADORA3	0.630735099	-0.664893878	0.023182773	0.52942603	yes	down
ENSSSCG00000006800	CD53	0.63102724	-0.664225811	0.016821742	0.485997014	yes	down
ENSSSCG00000006828	ATXN7L2	1.371905897	0.456181527	0.018823072	0.504122241	yes	up
ENSSSCG00000006836	ELAPOR1	1.562165086	0.643546922	0.00854788	0.389033038	yes	up
ENSSSCG00000006912	HFM1	0.543892551	-0.878606427	0.040734169	0.667123021	yes	down
ENSSSCG00000006917	LRRC8C	0.814368947	-0.296245545	0.04122313	0.667123021	yes	down
ENSSSCG00000006935	CLCA2	1.76264224	0.817739683	0.00070823	0.145734681	yes	up
ENSSSCG00000006987	SLC7A2	1.691138047	0.757994431	0.011755808	0.436740665	yes	up
ENSSSCG00000007032	PLAT	0.708124512	-0.497925038	0.014322762	0.457630538	yes	down
ENSSSCG00000007072	SPTLC3	1.295805906	0.373849639	0.043764281	0.678952552	yes	up
ENSSSCG00000007131	CST7	0.444548823	-1.169586221	0.000724353	0.145734681	yes	down
ENSSSCG00000007140	SMOX	0.704960778	-0.504385102	0.009336961	0.39876703	yes	down
ENSSSCG00000007179	STK35	1.227271936	0.295454953	0.004332273	0.323023561	yes	up
ENSSSCG00000007323	DSN1	1.33160185	0.41316278	0.003327204	0.295537114	yes	up
ENSSSCG00000007336	NNAT	1.453595214	0.539625575	0.002388239	0.25031708	yes	up
ENSSSCG00000007344	KIAA1755	0.44312708	-1.174207599	0.001508304	0.208200082	yes	down
ENSSSCG00000007350	PPP1R16B	0.672547256	-0.572292453	0.003696617	0.296453856	yes	down
ENSSSCG00000007382	PABPC1L	3.138865605	1.650243259	0.002754662	0.269792437	yes	up
ENSSSCG00000007435	PLTP	0.716334822	-0.481294019	0.035433615	0.633787122	yes	down
ENSSSCG00000007436	MMP9	0.270999952	-1.883635496	0.031246207	0.609556562	yes	down
ENSSSCG00000007456	SULF2	0.745217559	-0.424266428	0.020971635	0.513921244	yes	down
ENSSSCG00000007476	KCNG1	0.178407389	-2.486752725	0.025515298	1	yes	down
ENSSSCG00000007477	NFATC2	0.644977457	-0.632679358	0.000310771	0.098438311	yes	down
ENSSSCG00000007485	BCAS1	1.302734549	0.381543144	0.015020542	0.467600207	yes	up
ENSSSCG00000007507	PCK1	1.700901801	0.766299852	0.044636868	0.679681619	yes	up
ENSSSCG00000007522	CTSZ	0.665762681	-0.586920091	0.006370811	0.366967563	yes	down
ENSSSCG00000007543	DNAAF5	1.256648517	0.329581186	0.004486726	0.326473146	yes	up
ENSSSCG00000007574	SDK1	1.681877889	0.750072964	0.034296445	0.627268647	yes	up
ENSSSCG00000007718	CLDN4	0.631828125	-0.662395936	0.008105754	0.387495665	yes	down
ENSSSCG00000007722	NCF1	0.515969202	-0.95464314	0.001826841	0.215227738	yes	down
ENSSSCG00000007800	SEPTIN1	0.598453744	-0.740688352	0.005851215	0.362642915	yes	down
ENSSSCG00000007872	XYLT1	0.658227462	-0.603341876	0.023977461	0.540350362	yes	down
ENSSSCG00000007874	ABCC1	0.751704427	-0.411762595	0.027560473	0.572788782	yes	down
ENSSSCG00000007980	FAM234A	1.239723445	0.310018323	0.003948634	0.30956918	yes	up
ENSSSCG00000008056	ATPeV0C	0.748442027	-0.418037524	0.005150207	0.346159768	yes	down
ENSSSCG00000008073	OMD	2.210874138	1.144616897	0.037890436	0.647166387	yes	up
ENSSSCG00000008103	MERTK	0.599146467	-0.739019369	0.016694929	0.48592959	yes	down
ENSSSCG00000008123	ARID5A	0.705109708	-0.504080352	0.021530204	0.518074755	yes	down
ENSSSCG00000008141	ST6GAL2	1.847531419	0.885598899	0.000420026	0.120931382	yes	up
ENSSSCG00000008157	IL18RAP	0.322651451	-1.631951582	0.011033738	0.433668642	yes	down

ENSSSCG00000008159	IL18R1	0.723389125	-0.467156185	0.047346932	0.691288485	yes	down
ENSSSCG00000008228	GNLY	0.578319268	-0.790061925	0.041453433	0.667532188	yes	down
ENSSSCG00000008344	ARHGAP25	0.702756406	-0.508903396	0.000342989	0.104137791	yes	down
ENSSSCG00000008388	REL	0.750245717	-0.414564917	0.020272832	0.509931129	yes	down
ENSSSCG00000008392	BCL11A	1.385406104	0.470308935	0.01260707	0.44697551	yes	up
ENSSSCG00000008394	FANCL	1.46934485	0.555173031	0.007592555	0.387495665	yes	up
ENSSSCG00000008535	CLIP4	0.489471055	-1.030704546	0.047804235	0.691288485	yes	down
ENSSSCG00000008593	KLHL29	0.699874098	-0.514832679	0.035583905	0.633787122	yes	down
ENSSSCG00000008617	CYRIA	0.665597969	-0.587277063	0.012990695	0.448536088	yes	down
ENSSSCG00000008722	SH3TC1	1.314276847	0.394269205	0.043844189	0.679178206	yes	up
ENSSSCG00000008723	HTRA3	0.688788748	-0.53786652	0.00870955	0.390119267	yes	down
ENSSSCG00000008765	PCDH7	1.74325007	0.80177954	0.01417208	0.456766144	yes	up
ENSSSCG00000008769	CD68	0.316913104	-1.65784078	0.02521516	0.554956872	yes	down
ENSSSCG00000008794	RHOH	0.594184743	-0.751016534	0.009917155	0.414016408	yes	down
ENSSSCG00000008842	KIT	0.691116327	-0.532999533	0.001858137	0.215227738	yes	down
ENSSSCG00000008874	TMEM144	1.208419766	0.273121687	0.008241405	0.387495665	yes	up
ENSSSCG00000008937	AMBN	0.464080393	-1.107553348	0.011361398	0.436147095	yes	down
ENSSSCG00000008973	NAAA	0.525860117	-0.927249012	0.006597992	0.367294106	yes	down
ENSSSCG00000009002	TLR2	0.708818359	-0.496512123	0.028664585	0.58539213	yes	down
ENSSSCG00000009004	SFRP2	1.615475084	0.691958499	0.020946077	0.513921244	yes	up
ENSSSCG00000009015	gatB	1.215598846	0.28166721	0.019910617	0.506069104	yes	up
ENSSSCG00000009053	RNF150	1.227159159	0.295322375	0.010309344	0.417658959	yes	up
ENSSSCG00000009089	ADAD1	7.522242693	2.911162853	0.046557948	1	yes	up
ENSSSCG00000009122	ARSJ	0.638905977	-0.646324458	0.029889892	0.598270325	yes	down
ENSSSCG00000009138	CFI	0.600422057	-0.735951119	0.00108221	0.182543644	yes	down
ENSSSCG00000009140	CASP6	1.235251503	0.304804811	0.030776192	0.606766974	yes	up
ENSSSCG00000009148	LEF1	0.528132564	-0.921027996	0.006242926	0.366967563	yes	down
ENSSSCG00000009151	CYP2U1	0.786945894	-0.345663647	0.011099882	0.434053184	yes	down
ENSSSCG00000009166	MANBA	0.668414241	-0.581185623	0.028675415	0.58539213	yes	down
ENSSSCG00000009225	HSD17B11	1.227577965	0.295814655	0.00348618	0.296453856	yes	up
ENSSSCG00000009276	XPO4	1.218366389	0.284948048	0.043382795	0.678952552	yes	up
ENSSSCG00000009314	FLT3	0.648754714	-0.624254978	0.046066558	0.687457963	yes	down
ENSSSCG00000009316	PRHOXNB	1.554310376	0.63627462	0.030668961	0.606072765	yes	up
ENSSSCG00000009348	STARD13	1.211818389	0.277173503	0.006739315	0.368533767	yes	up
ENSSSCG00000009370	FOXO1	0.746446964	-0.421888337	0.015253454	0.467600207	yes	down
ENSSSCG00000009378	CKAP2	1.264631987	0.338717616	0.040660698	0.667123021	yes	up
ENSSSCG00000009407	ESD	0.715207876	-0.48356547	0.005141185	0.346159768	yes	down
ENSSSCG00000009412	LCP1	0.622444152	-0.683983696	0.006478082	0.366967563	yes	down
ENSSSCG00000009490	DCT	1.508788086	0.593390189	0.041868326	0.667913669	yes	up
ENSSSCG00000009498	ABCC4	1.35320771	0.436383303	0.008466839	0.389033038	yes	up
ENSSSCG00000009517	GPR183	0.675709272	-0.565525444	0.013002788	0.448536088	yes	down

ENSSSCG00000009519	CLYBL	1.317000256	0.397255626	0.005533333	0.358143884	yes	up
ENSSSCG00000009527	FGF14	3.86105594	1.948995456	0.009316182	0.39876703	yes	up
ENSSSCG00000009567	RASA3	0.794654105	-0.33160107	0.022184001	0.522737311	yes	down
ENSSSCG00000009612	DOK2	0.631395507	-0.663384101	0.003453468	0.296453856	yes	down
ENSSSCG00000009650	DOCK5	1.220967338	0.288024608	0.028576483	0.58539213	yes	up
ENSSSCG00000009665	CHRNA2	0.080829912	-3.628966919	0.037306138	1	yes	down
ENSSSCG00000009768	TCTN2	1.299327564	0.377765184	0.01764605	0.492991747	yes	up
ENSSSCG00000009784	ABCB9	1.310454215	0.390066949	0.006782468	0.368533767	yes	up
ENSSSCG00000009807	RHOF	0.676951049	-0.562876581	0.000954935	0.169643116	yes	down
ENSSSCG00000009833	SH2B3	0.794586625	-0.331723585	0.017533072	0.492991747	yes	down
ENSSSCG00000009836	ACAD10	1.506323888	0.59103201	3.00E-07	0.001003188	yes	up
ENSSSCG00000009859	TESC	0.670045923	-0.577668119	0.04138842	0.667128602	yes	down
ENSSSCG00000009866	SCAN	8.5798126	3.100946137	0.037921264	1	yes	up
ENSSSCG00000009877	IQCD	0.614959866	-0.701435836	0.004597365	0.32808287	yes	down
ENSSSCG00000009879	TPCN1	0.821922795	-0.28292521	0.006330543	0.366967563	yes	down
ENSSSCG00000009919	HNF1A	1.238533724	0.308633152	0.019435713	0.504239817	yes	up
ENSSSCG00000009943	SSH1	0.812306056	-0.299904694	0.020006298	0.507728224	yes	down
ENSSSCG00000009944	CORO1C	0.778646465	-0.360959656	1.84E-05	0.025633123	yes	down
ENSSSCG00000009963	TPST2	0.756629978	-0.402340156	0.030680898	0.606072765	yes	down
ENSSSCG00000009999	TBC1D10A	0.771148901	-0.374918638	0.025223802	0.554956872	yes	down
ENSSSCG00000010101	P2RX6	2.048112061	1.034294654	0.039123166	0.65792321	yes	up
ENSSSCG00000010161	COA6	1.217288254	0.283670839	0.020304812	0.509931129	yes	up
ENSSSCG00000010188	ABCB10	1.221269001	0.288381008	0.009124708	0.396883703	yes	up
ENSSSCG00000010283	SPOCK2	0.695062919	-0.524784514	0.001563386	0.209336848	yes	down
ENSSSCG00000010302	USP54	1.242090398	0.312770175	0.00456429	0.327120488	yes	up
ENSSSCG00000010352	GRID1	0.358835645	-1.478604886	0.047507266	0.691288485	yes	down
ENSSSCG00000010391	ERCC6	1.351190289	0.434230865	0.014669007	0.46161769	yes	up
ENSSSCG00000010450	LIPA	0.610158584	-0.712743837	0.001506909	0.208200082	yes	down
ENSSSCG00000010451	IFIT2	4.236679982	2.082934159	0.003760565	0.29903657	yes	up
ENSSSCG00000010459	HTR7	0.541476954	-0.885028159	0.047735097	0.691288485	yes	down
ENSSSCG00000010493	PDLIM1	0.83119093	-0.266748183	0.011037138	0.433668642	yes	down
ENSSSCG00000010497	ENTPD1	0.805805335	-0.311496738	0.047117275	0.691288485	yes	down
ENSSSCG00000010533	PYROXD2	1.458019616	0.544010129	0.014393037	0.457630538	yes	up
ENSSSCG00000010601	CALHM1	0.104696332	-3.255717196	0.010046721	1	yes	down
ENSSSCG00000010639	HABP2	5.158656254	2.366995316	0.029992721	1	yes	up
ENSSSCG00000010699	ATE1	1.328195406	0.409467413	0.004198765	0.323023561	yes	up
ENSSSCG00000010816	TGFB2	0.676440656	-0.563964723	0.017548866	0.492991747	yes	down
ENSSSCG00000010833	TAF1A	1.327573848	0.408792115	0.047884344	0.691288485	yes	up
ENSSSCG00000010837	FAM177B	1.428166811	0.514164497	0.015611176	0.471412346	yes	up
ENSSSCG00000010853	EPHX1	0.790834085	-0.338553042	0.02563432	0.5583584	yes	down
ENSSSCG00000010861	COQ8A	1.374456061	0.458860787	0.000679679	0.145734681	yes	up

ENSSSCG00000010893	CFH	0.619588234	-0.690618348	0.011984687	0.439315508	yes	down
ENSSSCG00000010908	PTPRC	0.671578839	-0.574371322	0.022557422	0.524686071	yes	down
ENSSSCG00000010948	CTSL	0.662359721	-0.594313151	0.036454567	0.639448339	yes	down
ENSSSCG00000011033	VIM	0.785642742	-0.348054676	0.014414502	0.457630538	yes	down
ENSSSCG00000011043	C1QL3	3.446082936	1.784957423	0.001794254	0.215227738	yes	up
ENSSSCG00000011129	ITIH5	0.737838059	-0.438623887	0.012691681	0.447694574	yes	down
ENSSSCG00000011198	RFTN1	0.736520427	-0.441202557	0.00016791	0.068714733	yes	down
ENSSSCG00000011299	CLEC3B	1.242659966	0.313431579	0.01335228	0.449667171	yes	up
ENSSSCG00000011322	CCR1	0.613982005	-0.703731722	0.036726341	0.640849708	yes	down
ENSSSCG00000011361	SLC26A6	0.667608819	-0.582925083	0.02636739	0.566678299	yes	down
ENSSSCG00000011398	SEMA3F	0.821875804	-0.283007694	0.043788399	0.678952552	yes	down
ENSSSCG00000011399	GNAI2	0.771294893	-0.374645536	0.000304801	0.098438311	yes	down
ENSSSCG00000011423	GRM2	1.596045662	0.674501927	0.022579812	0.524686071	yes	up
ENSSSCG00000011434	PPM1M	0.787225459	-0.345151216	0.008657806	0.389694612	yes	down
ENSSSCG00000011436	TLR9	0.584930223	-0.77366356	0.013304076	0.449667171	yes	down
ENSSSCG00000011441	TNNC1	0.249139078	-2.004976765	0.005169684	0.346159768	yes	down
ENSSSCG00000011471	FLNB	1.26874974	0.343407526	0.006430764	0.366967563	yes	up
ENSSSCG00000011511	FRMD4B	0.68864103	-0.538175954	0.017822194	0.492991747	yes	down
ENSSSCG00000011570	IRAK2	0.674347916	-0.568434983	0.009980686	0.414485269	yes	down
ENSSSCG00000011592	PLXND1	0.716618874	-0.480722053	0.000141774	0.068714733	yes	down
ENSSSCG00000011826	TMEM44	1.21699877	0.283327709	0.020091186	0.508965388	yes	up
ENSSSCG00000011860	SLC12A8	1.841662972	0.88100907	7.91E-05	0.062885185	yes	up
ENSSSCG00000011884	HCLS1	0.652469048	-0.616018631	0.008224849	0.387495665	yes	down
ENSSSCG00000011895	PLA1A	1.320332555	0.40090135	0.027125953	0.571323123	yes	up
ENSSSCG00000012000	GBE1	1.338983574	0.421138263	0.010828827	0.429526338	yes	up
ENSSSCG00000012001	ROBO1	0.80582481	-0.311461871	0.021999695	0.52018032	yes	down
ENSSSCG00000012020	NCAM2	2.604299971	1.380895632	0.011530155	0.436740665	yes	up
ENSSSCG00000012030	CLDN8	16.48735257	4.043287854	7.86E-05	0.062885185	yes	up
ENSSSCG00000012117	TLR7	0.633064616	-0.659575335	0.046583669	0.690853194	yes	down
ENSSSCG00000012124	TRAPPC2	1.247463184	0.318997238	0.02182431	0.52018032	yes	up
ENSSSCG00000012125	OFD1	0.731148801	-0.451763047	0.003611774	0.296453856	yes	down
ENSSSCG00000012127	GEMIN8	0.777373051	-0.363321	0.021119277	0.513921244	yes	down
ENSSSCG00000012132	ASB9	0.503395762	-0.990235023	0.011591819	0.436740665	yes	down
ENSSSCG00000012155	SCML2	1.491119236	0.576395626	0.009751371	0.412248475	yes	up
ENSSSCG00000012278	CFP	0.753711619	-0.407915461	0.049248009	0.696771975	yes	down
ENSSSCG00000012292	SYP	1.51432796	0.598677685	0.031035832	0.607254547	yes	up
ENSSSCG00000012335	FGD1	0.769148373	-0.378666165	0.036552885	0.63982874	yes	down
ENSSSCG00000012352	KLF8	2.317885572	1.212809346	0.003422124	0.296453856	yes	up
ENSSSCG00000012365	MSN	0.797077198	-0.327208636	0.000318323	0.098438311	yes	down
ENSSSCG00000012397	IL2RG	0.601002897	-0.734556149	0.000667758	0.145734681	yes	down
ENSSSCG00000012446	P2RY10	0.680630669	-0.555055936	0.035993689	0.634028076	yes	down

ENSSSCG00000012517	TMSB15	0.659354196	-0.600874425	0.002836259	0.273772805	yes	down
ENSSSCG00000012545	IL1RAPL2	10.01430323	3.32399014	0.006940278	1	yes	up
ENSSSCG00000012548	PWWP3B	1.457124096	0.54312375	0.049419177	0.696771975	yes	up
ENSSSCG00000012584	CAPN6	0.089696152	-3.478810095	0.010060908	1	yes	down
ENSSSCG00000012634	DOCK11	0.699437362	-0.515733233	0.033823928	0.626076307	yes	down
ENSSSCG00000012652	SASH3	0.706571166	-0.501093219	0.029045425	0.590778982	yes	down
ENSSSCG00000012790	ARHGAP4	0.706549819	-0.501136806	0.022716811	0.524686071	yes	down
ENSSSCG00000012824	GAB3	0.672990186	-0.571342629	0.014389078	0.457630538	yes	down
ENSSSCG00000012844	SLC25A22	0.80735892	-0.308717913	0.035904103	0.633787122	yes	down
ENSSSCG00000012909	PTPRCAP	0.624720813	-0.678716499	0.004938068	0.343586664	yes	down
ENSSSCG00000012912	TBC1D10C	0.633539356	-0.658493852	0.025854901	0.561131752	yes	down
ENSSSCG00000012934	CCS	0.794348963	-0.332155162	0.049033653	0.695594658	yes	down
ENSSSCG00000012960	CST6	1.34579549	0.428459192	0.02421538	0.543511592	yes	up
ENSSSCG00000012968	CCDC85B	0.794217505	-0.332393936	0.021949071	0.52018032	yes	down
ENSSSCG00000012975	SNX32	2.399780507	1.262902458	0.044228074	0.679567425	yes	up
ENSSSCG00000013030	PRDX5	0.716567201	-0.480826086	0.001278625	0.19820514	yes	down
ENSSSCG00000013042	CCDC88B	0.441247984	-1.180338408	0.000227311	0.082219172	yes	down
ENSSSCG00000013111	CD6	0.651767643	-0.617570363	0.046478224	0.690193347	yes	down
ENSSSCG00000013115	CD5	0.606922219	-0.720416456	0.006255828	0.366967563	yes	down
ENSSSCG00000013181	SERPING1	0.740894573	-0.432659828	0.035247278	0.633787122	yes	down
ENSSSCG00000013226	PTPMT1	1.268063036	0.342626464	0.025874091	0.561131752	yes	up
ENSSSCG00000013237	SPI1	0.620443065	-0.688629267	0.002466781	0.255855774	yes	down
ENSSSCG00000013246	CSTPP1	0.633641565	-0.65826112	0.002189986	0.238317669	yes	down
ENSSSCG00000013260	MDK	0.733709477	-0.446719175	0.00750458	0.385596862	yes	down
ENSSSCG00000013273	CHST1	0.739644026	-0.435096993	0.035710209	0.633787122	yes	down
ENSSSCG00000013276	PRDM11	1.347776324	0.430581088	0.044658028	0.679681619	yes	up
ENSSSCG00000013297	CD44	0.770038791	-0.37699697	0.005805969	0.361768194	yes	down
ENSSSCG00000013360	TMEM86A	0.491641054	-1.024322703	6.44E-05	0.060359762	yes	down
ENSSSCG00000013369	SAA	0.093064265	-3.425628881	0.033325592	1	yes	down
ENSSSCG00000013382	PLEKHA7	1.313470391	0.393383679	0.002212063	0.238317669	yes	up
ENSSSCG00000013408	ADM	0.750146384	-0.414755943	0.041710471	0.667913669	yes	down
ENSSSCG00000013498	EBI3	0.581226707	-0.782827099	0.011968954	0.439315508	yes	down
ENSSSCG00000013545	TUBB4A	0.592099996	-0.756087251	0.012300637	0.442351644	yes	down
ENSSSCG00000013550	SLC25A23	0.824422615	-0.278544014	0.033724365	0.626076307	yes	down
ENSSSCG00000013553	VAV1	0.685925885	-0.543875394	0.014574459	0.460803078	yes	down
ENSSSCG00000013579	CD209	0.338839336	-1.561326726	0.009863885	0.413744596	yes	down
ENSSSCG00000013599	ANGPTL4	0.528030049	-0.921308063	2.38E-05	0.030519227	yes	down
ENSSSCG00000013604	MYO1F	0.616291036	-0.698316288	0.002284837	0.241901682	yes	down
ENSSSCG00000013620	CCDC159	0.529259329	-0.9179533	0.00650714	0.366967563	yes	down
ENSSSCG00000013642	PDE4A	0.716329508	-0.481304722	0.002753768	0.269792437	yes	down
ENSSSCG00000013649	ICAM3	0.543532279	-0.87956238	0.003652266	0.296453856	yes	down

ENSSSCG00000013655	ICAM1	0.711369117	-0.491329751	0.027303156	0.572783175	yes	down
ENSSSCG00000013731	DNASE2	0.619528069	-0.690758448	0.001695947	0.211347945	yes	down
ENSSSCG00000013758	ZSWIM4	0.757405753	-0.400861715	0.026207877	0.56470366	yes	down
ENSSSCG00000013862	MED26	0.602427695	-0.73114	0.039823212	0.662872245	yes	down
ENSSSCG00000013885	FCHO1	0.655250053	-0.60988253	0.013315676	0.449667171	yes	down
ENSSSCG00000013888	JAK3	0.70240215	-0.509630834	0.037848768	0.647166387	yes	down
ENSSSCG00000013895	MAST3	0.810505977	-0.303105269	0.017555535	0.492991747	yes	down
ENSSSCG00000013901	IFI30	0.6311347	-0.66398015	0.001258034	0.198187821	yes	down
ENSSSCG00000013973	LYPD8	0.521586639	-0.939021182	0.001687266	0.211347945	yes	down
ENSSSCG00000014012	GFPT2	0.600349782	-0.736124791	0.039893802	0.662872245	yes	down
ENSSSCG00000014170	CAST	1.202778995	0.266371579	0.007438593	0.385459407	yes	up
ENSSSCG00000014274	PDLIM4	0.626836002	-0.673840052	0.000111329	0.068003035	yes	down
ENSSSCG00000014303	JADE2	1.216232297	0.282418806	0.004718259	0.333646722	yes	up
ENSSSCG00000014316	TGFBI	0.638899563	-0.646338941	0.006168011	0.366967563	yes	down
ENSSSCG00000014348	SPATA24	0.736887118	-0.440484461	0.013259107	0.449667171	yes	down
ENSSSCG00000014349	STING1	0.753196495	-0.408901808	0.017434967	0.492991747	yes	down
ENSSSCG00000014361	CYSTM1	0.690859705	-0.533535327	1.21E-06	0.002608747	yes	down
ENSSSCG00000014369	CD14	0.550051776	-0.862360669	0.001379504	0.198589146	yes	down
ENSSSCG00000014431	AFAP1L1	0.737375987	-0.43952766	0.035188518	0.633787122	yes	down
ENSSSCG00000014441	CSF1R	0.615072228	-0.701172258	0.01152061	0.436740665	yes	down
ENSSSCG00000014540	FTH1	0.777004408	-0.364005312	0.045916276	0.687457963	yes	down
ENSSSCG00000014812	FOLR2	0.758257906	-0.39923946	0.040613015	0.667123021	yes	down
ENSSSCG00000014813	PHOX2A	0.101251979	-3.303977994	0.013663482	1	yes	down
ENSSSCG00000014874	B3GNT6	1.732425367	0.792793203	0.003589786	0.296453856	yes	up
ENSSSCG00000014917	CCDC81	1.449415484	0.535471211	0.032971057	0.621426271	yes	up
ENSSSCG00000014924	CTSC	0.648440683	-0.624953487	0.006538088	0.366967563	yes	down
ENSSSCG00000014960	AMOTL1	1.397257384	0.482597799	0.036985781	0.643359952	yes	up
ENSSSCG00000015016	POU2AF1	0.563718193	-0.826953967	0.007855573	0.387495665	yes	down
ENSSSCG00000015022	LAYN	0.70774966	-0.498688943	0.027078514	0.571323123	yes	down
ENSSSCG00000015069	APOC3	0.145259859	-2.78329201	0.000559405	0.144078411	yes	down
ENSSSCG00000015082	DSCAML1	1.432815204	0.518852552	0.035863456	0.633787122	yes	up
ENSSSCG00000015083	FXYD6	0.794026583	-0.332740786	0.003308876	0.295480817	yes	down
ENSSSCG00000015085	IL10RA	0.604314431	-0.726628701	0.019798631	0.504766715	yes	down
ENSSSCG00000015093	CD3D	0.688930478	-0.537569691	0.0417967	0.667913669	yes	down
ENSSSCG00000015113	ABCG4	0.321050571	-1.639127532	0.026756077	0.57062545	yes	down
ENSSSCG00000015137	CRTAM	0.498801215	-1.003463115	0.030498661	0.604148451	yes	down
ENSSSCG00000015138	JHY	0.376732708	-1.4083868	0.048624942	0.694006759	yes	down
ENSSSCG00000015144	GRAMD1B	0.651728356	-0.617657329	9.23E-05	0.065519059	yes	down
ENSSSCG00000015145	ZNF202	1.324446832	0.40538993	0.026869356	0.571323123	yes	up
ENSSSCG00000015235	ETS1	0.77963654	-0.359126387	0.007735294	0.387495665	yes	down
ENSSSCG00000015237	FLI1	0.789629746	-0.340751757	0.000787022	0.15198493	yes	down

ENSSSCG00000015249	ADAMTS8	0.682788298	-0.550489762	0.034664733	0.629887241	yes	down
ENSSSCG00000015268	FMO1	2.279899649	1.188970325	0.000831342	0.154772348	yes	up
ENSSSCG00000015270	FMOD	0.826378673	-0.275125074	0.039821121	0.662872245	yes	down
ENSSSCG00000015281	PLEKHA6	1.280650618	0.35687694	0.016432404	0.484426375	yes	up
ENSSSCG00000015299	STEAP4	0.375287935	-1.413930185	0.00652782	0.366967563	yes	down
ENSSSCG00000015363	AGR2	1.463667792	0.549588143	0.00566755	0.359764441	yes	up
ENSSSCG00000015405	CD36	0.578744296	-0.789002026	0.001362949	0.198589146	yes	down
ENSSSCG00000015492	DARS2	1.227712788	0.295973096	0.023717432	0.536663133	yes	up
ENSSSCG00000015512	PAPPA2	1.76456058	0.819308961	0.008062232	0.387495665	yes	up
ENSSSCG00000015559	NCF2	0.686226432	-0.543243398	0.006792421	0.368533767	yes	down
ENSSSCG00000015563	RGL1	0.744209502	-0.426219285	0.017592254	0.492991747	yes	down
ENSSSCG00000015569	SWT1	1.223345077	0.290831411	0.045629962	0.687082715	yes	up
ENSSSCG00000015589	VASH2	0.736246305	-0.441739607	0.03287159	0.621426271	yes	down
ENSSSCG00000015595	ATF3	0.406308263	-1.299353391	0.049610648	0.696771975	yes	down
ENSSSCG00000015603	LPGAT1	1.200497765	0.263632717	0.023168738	0.52942603	yes	up
ENSSSCG00000015638	RHEX	0.513587109	-0.961319103	0.002273487	0.241901682	yes	down
ENSSSCG00000015656	FCMR	0.532109059	-0.910206128	0.019715937	0.504766715	yes	down
ENSSSCG00000015662	C4BPA	0.27045921	-1.886517066	0.005709179	0.359764441	yes	down
ENSSSCG00000015664	CD55	0.552045263	-0.857141536	0.026120123	0.563539972	yes	down
ENSSSCG00000015711	DPP10	3.745706996	1.905238051	0.004502453	0.326473146	yes	up
ENSSSCG00000015781	ENPP6	0.662343459	-0.594348573	0.043678279	0.678952552	yes	down
ENSSSCG00000015792	LRP2BP	1.676348246	0.745321886	0.02538899	0.557856247	yes	up
ENSSSCG00000015795	—	0.219677899	-2.186538361	0.043607165	1	yes	down
ENSSSCG00000015801	TLR3	1.269749044	0.344543388	0.005915024	0.364483343	yes	up
ENSSSCG00000015826	yggS	1.29123412	0.368750607	0.001369231	0.198589146	yes	up
ENSSSCG00000015828	ZNF703	1.204200937	0.268076145	0.008161402	0.387495665	yes	up
ENSSSCG00000015846	RBPMS	0.752796469	-0.409668234	0.032879041	0.621426271	yes	down
ENSSSCG00000015917	XIRP2	0.415858312	-1.265836027	0.010155999	0.416773906	yes	down
ENSSSCG00000015949	SLC25A12	0.775848077	-0.366153917	0.047523102	0.691288485	yes	down
ENSSSCG00000016059	STAT4	0.649317274	-0.623004505	0.013470342	0.449667171	yes	down
ENSSSCG00000016095	CLK1	1.211592189	0.276904183	0.024150814	0.543299782	yes	up
ENSSSCG00000016125	INO80D	1.22966472	0.298265005	0.032085222	0.614363628	yes	up
ENSSSCG00000016131	ADAM23	0.601763765	-0.732730857	0.000310013	0.098438311	yes	down
ENSSSCG00000016159	CPS1	0.152457358	-2.713522318	0.031510536	1	yes	down
ENSSSCG00000016174	FN1	0.814085172	-0.296748354	0.006426552	0.366967563	yes	down
ENSSSCG00000016184	CXCR2	0.334550945	-1.579702177	0.000145555	0.068714733	yes	down
ENSSSCG00000016298	INPP5D	0.646307798	-0.629706697	0.003686898	0.296453856	yes	down
ENSSSCG00000016314	TRPM8	2.988203834	1.579278562	0.03562837	0.633787122	yes	up
ENSSSCG00000016322	ACKR3	0.73936341	-0.435644446	0.006148846	0.366967563	yes	down
ENSSSCG00000016436	CHPF2	0.802831002	-0.316831766	0.015120401	0.467600207	yes	down
ENSSSCG00000016443	—	0.737445908	-0.439390864	0.032201859	0.614363628	yes	down

ENSSSCG00000016461	ZYX	0.804015124	-0.314705454	0.042651129	0.67382328	yes	down
ENSSSCG00000016469	TRPV6	0.618538166	-0.693065478	0.03821297	0.64849429	yes	down
ENSSSCG00000016475	TRBV	0.646718237	-0.628790802	0.001488943	0.208200082	yes	down
ENSSSCG00000016513	KIAA1549	1.520263601	0.604321497	0.031478154	0.611936786	yes	up
ENSSSCG00000016522	PTN	1.544968257	0.627577196	0.027936595	0.578083275	yes	up
ENSSSCG00000016557	CPA1	0.470630384	-1.087333631	0.044393503	0.679681619	yes	down
ENSSSCG00000016619	ING3	1.273972225	0.349333824	0.04960336	0.696771975	yes	up
ENSSSCG00000016642	GPR85	2.074804038	1.052975083	0.003253049	0.292057385	yes	up
ENSSSCG00000016646	IFRD1	0.718305738	-0.477330054	0.040697468	0.667123021	yes	down
ENSSSCG00000016703	HOXA5	1.309218615	0.38870602	0.037438834	0.647166387	yes	up
ENSSSCG00000016742	MYO1G	0.666598527	-0.585109965	0.016373167	0.484426375	yes	down
ENSSSCG00000016751	GCK	0.571357261	-0.807534971	0.006939419	0.373810834	yes	down
ENSSSCG00000016756	BLVRA	0.777525673	-0.363037783	0.001521074	0.208200082	yes	down
ENSSSCG00000016763	GLI3	0.772825054	-0.37178623	0.046482752	0.690193347	yes	down
ENSSSCG00000016832	IL7R	0.501927794	-0.994448258	2.75E-11	2.30E-07	yes	down
ENSSSCG00000016843	CPLANE1	1.424458502	0.510413593	0.000168711	0.068714733	yes	up
ENSSSCG00000016859	C7	1.555503216	0.637381378	0.002094162	0.234700735	yes	up
ENSSSCG00000016867	CCDC152	0.643896416	-0.635099476	0.009406216	0.400352029	yes	down
ENSSSCG00000016892	FST	1.598555128	0.676768498	0.036508197	0.63971709	yes	up
ENSSSCG00000016898	CSPG4	2.031211289	1.022340318	0.004991896	0.345450877	yes	up
ENSSSCG00000016900	ESM1	0.553772405	-0.852634931	0.019156201	0.504239817	yes	down
ENSSSCG00000016943	ADAMTS6	1.395741659	0.481031934	0.047543407	0.691288485	yes	up
ENSSSCG00000016976	ZNF366	0.747567181	-0.419724861	0.007757054	0.387495665	yes	down
ENSSSCG00000017006	LCP2	0.642204387	-0.638895575	0.009885867	0.413744596	yes	down
ENSSSCG00000017044	IL12B	0.204043199	-2.293053467	0.024143501	1	yes	down
ENSSSCG00000017052	ADAM19	0.60631887	-0.721851372	0.000791825	0.15198493	yes	down
ENSSSCG00000017054	CYFIP2	0.662997486	-0.592924694	0.013299561	0.449667171	yes	down
ENSSSCG00000017062	TIMD4	0.467372944	-1.097353876	0.016518843	0.484750143	yes	down
ENSSSCG00000017082	SPARC	0.810800018	-0.302581973	0.002762711	0.269792437	yes	down
ENSSSCG00000017089	ANXA6	0.755950645	-0.403636048	0.001771477	0.215227738	yes	down
ENSSSCG00000017095	SEMA5A	1.398826875	0.484217419	0.006314965	0.366967563	yes	up
ENSSSCG00000017164	TIMP2	0.789437316	-0.34110338	0.020558344	0.511065793	yes	down
ENSSSCG00000017203	GALK1	0.751561874	-0.412036213	0.003562323	0.296453856	yes	down
ENSSSCG00000017230	TMEM104	0.734474382	-0.445215924	0.00570027	0.359764441	yes	down
ENSSSCG00000017233	RAB37	0.630768657	-0.66481712	0.02757783	0.572788782	yes	down
ENSSSCG00000017236	CD300C	0.592099969	-0.756087317	0.020360875	0.510519907	yes	down
ENSSSCG00000017237	CD300LB	0.411218684	-1.28202228	0.037528589	0.647166387	yes	down
ENSSSCG00000017297	CYB561	1.346838738	0.429577122	0.001281882	0.19820514	yes	up
ENSSSCG00000017333	FMNL1	0.732416753	-0.449263304	0.032464722	0.61746241	yes	down
ENSSSCG00000017355	GRN	0.787997062	-0.343737844	0.036610073	0.640114472	yes	down
ENSSSCG00000017392	CCR10	0.604602333	-0.725941549	0.044790779	0.679964741	yes	down

ENSSSCG00000017466	CCR7	0.528041572	-0.92127658	0.023168664	0.52942603	yes	down
ENSSSCG00000017494	IKZF3	0.651356861	-0.61847992	0.032902915	0.621426271	yes	down
ENSSSCG00000017507	TFCP2	1.868404356	0.901806714	0.005255732	0.348080505	yes	up
ENSSSCG00000017539	HOXB6	1.310423876	0.390033549	0.044209668	0.679567425	yes	up
ENSSSCG00000017540	HOXB5	1.708154096	0.772438129	0.000675692	0.145734681	yes	up
ENSSSCG00000017551	FAM117A	0.765386756	-0.385739158	0.033787906	0.626076307	yes	down
ENSSSCG00000017589	DLX3	0.327280603	-1.611399995	0.038762671	0.654030728	yes	down
ENSSSCG00000017605	MMD	1.304556985	0.383559965	0.046006925	0.687457963	yes	up
ENSSSCG00000017617	SCPEP1	0.605880429	-0.722894989	0.004260868	0.323023561	yes	down
ENSSSCG00000017682	MYO19	1.330318352	0.411771532	0.011812674	0.437383259	yes	up
ENSSSCG00000017705	CCL5	0.545298828	-0.87488104	0.003145308	0.288459657	yes	down
ENSSSCG00000017738	ADAP2	0.626202673	-0.675298427	0.01362236	0.449667171	yes	down
ENSSSCG00000017749	EVI2A	0.628006965	-0.671147536	0.015275084	0.467600207	yes	down
ENSSSCG00000017750	EVI2B	0.644814535	-0.633043829	0.004256332	0.323023561	yes	down
ENSSSCG00000017753	KSR1	1.225413962	0.293269194	0.033920382	0.626076307	yes	up
ENSSSCG00000017781	PIPOX	3.753142778	1.908099175	0.019475322	0.504239817	yes	up
ENSSSCG00000017797	SLC6A4	0.299000312	-1.741781103	0.019790704	0.504766715	yes	down
ENSSSCG00000017803	TIMM22	0.792266069	-0.335943079	0.044922014	0.680188307	yes	down
ENSSSCG00000017818	SERPINF1	0.709334482	-0.495462014	0.002566502	0.259185208	yes	down
ENSSSCG00000017873	CAMKK1	0.725479219	-0.462993806	0.025187299	0.554956872	yes	down
ENSSSCG00000017890	PITPNM3	1.264481611	0.338546057	0.011084558	0.434053184	yes	up
ENSSSCG00000017896	SCIMP	0.594097666	-0.751227975	0.005177508	0.346159768	yes	down
ENSSSCG00000017918	ARRB2	0.751626731	-0.411911718	0.006797317	0.368533767	yes	down
ENSSSCG00000017925	SLC16A11	0.692748184	-0.529597072	0.013579694	0.449667171	yes	down
ENSSSCG00000017938	YBX2	0.705985404	-0.502289738	0.023509455	0.532678954	yes	down
ENSSSCG00000017956	CD68	0.487020755	-1.037944839	6.99E-05	0.061429922	yes	down
ENSSSCG00000017963	DNAH2	0.292288457	-1.774535241	0.047564626	0.691288485	yes	down
ENSSSCG00000018061	—	0.639449213	-0.645098314	0.028561968	0.58539213	yes	down
ENSSSCG00000018063	—	0.599997523	-0.736971551	0.008158363	0.387495665	yes	down
ENSSSCG00000020749	KCNAB2	0.713423548	-0.487169259	0.013039133	0.448536088	yes	down
ENSSSCG00000020783	SLC41A1	0.767960724	-0.380895566	0.010901357	0.430808481	yes	down
ENSSSCG00000020813	FAM20C	1.523435784	0.607328689	0.007837227	0.387495665	yes	up
ENSSSCG00000020823	BSCL2	0.757277736	-0.401105581	0.007691409	0.387495665	yes	down
ENSSSCG00000020963	EPDR1	0.671805703	-0.573884051	0.041161315	0.667123021	yes	down
ENSSSCG00000021158	MCF2L	1.493196764	0.578404288	5.50E-08	0.000229747	yes	up
ENSSSCG00000021220	CKB	0.816084682	-0.293209232	0.045576663	0.687082715	yes	down
ENSSSCG00000021238	STX1B	0.672827525	-0.571691368	0.025968773	0.562454648	yes	down
ENSSSCG00000021467	EME2	1.364720431	0.448605439	0.017992018	0.494973152	yes	up
ENSSSCG00000021569	MMP25	0.494979268	-1.014559994	0.003178428	0.288459657	yes	down
ENSSSCG00000021624	—	1.263561023	0.337495341	0.006324893	0.366967563	yes	up
ENSSSCG00000021702	XRRA1	1.443641296	0.529712317	0.044785288	0.679964741	yes	up

ENSSSCG00000021706	KIF21B	0.709068902	-0.496002271	0.019304838	0.504239817	yes	down
ENSSSCG00000021818	ENOX1	1.954074066	0.966485152	0.044447286	0.679681619	yes	up
ENSSSCG00000021997	ALS2CL	1.520428347	0.604477829	0.008598643	0.389694612	yes	up
ENSSSCG00000022004	SH3BP2	0.784615204	-0.349942803	0.048998489	0.695594658	yes	down
ENSSSCG00000022029	RAP1GAP	1.29716462	0.37536158	0.035469487	0.633787122	yes	up
ENSSSCG00000022039	STARD9	0.77617229	-0.365551166	0.049428788	0.696771975	yes	down
ENSSSCG00000022176	—	0.762093339	-0.391960389	0.001820999	0.215227738	yes	down
ENSSSCG00000022236	FOLR	0.624191992	-0.679938247	0.021722951	0.519793188	yes	down
ENSSSCG00000022292	NPL	0.592892338	-0.754157941	0.005026922	0.345450877	yes	down
ENSSSCG00000022309	GPR34	0.65724817	-0.605489875	0.02267508	0.524686071	yes	down
ENSSSCG00000022337	SERPINB11	0.172737401	-2.533347605	0.034038294	1	yes	down
ENSSSCG00000022506	COL6A1	0.778056972	-0.362052297	0.033531108	0.626076307	yes	down
ENSSSCG00000022614	DQX1	1.504621004	0.589400135	0.049021158	0.695594658	yes	up
ENSSSCG00000022675	NCR1	0.416666031	-1.263036606	0.041034732	0.667123021	yes	down
ENSSSCG00000022689	GADD45B	0.780477558	-0.357570945	0.040057105	0.66360476	yes	down
ENSSSCG00000022925	SLC2A3	0.768051179	-0.380725647	0.020838389	0.513921244	yes	down
ENSSSCG00000022989	ZNF704	2.285414433	1.192455805	0.04464545	0.679681619	yes	up
ENSSSCG00000023033	PARP3	0.784932842	-0.349358871	0.026674048	0.570332821	yes	down
ENSSSCG00000023080	TPP1	0.818634662	-0.288708342	0.024663909	0.548462011	yes	down
ENSSSCG00000023169	CA10	1.835157494	0.875903881	0.031893149	0.612165162	yes	up
ENSSSCG00000023235	MAN1C1	0.749653777	-0.415703645	0.006756436	0.368533767	yes	down
ENSSSCG00000023279	SH3TC2	0.658488352	-0.602770173	0.035892589	0.633787122	yes	down
ENSSSCG00000023298	SRXN1	0.657411552	-0.605131286	0.019583241	0.504766715	yes	down
ENSSSCG00000023325	ACY1	0.730326337	-0.453386837	0.000141714	0.068714733	yes	down
ENSSSCG00000023329	APC2	1.634721293	0.709044689	0.012408232	0.443569791	yes	up
ENSSSCG00000023333	FCN2	1.817830271	0.862217503	0.000480611	0.129447139	yes	up
ENSSSCG00000023374	SRGN	0.589954664	-0.761324002	0.003103297	0.288459657	yes	down
ENSSSCG00000023377	EMP3	0.825366051	-0.276893996	0.040388061	0.667123021	yes	down
ENSSSCG00000023498	HSPB6	0.827899253	-0.272472878	0.019105507	0.504239817	yes	down
ENSSSCG00000023533	ZNF236	1.215179548	0.281169494	0.041999537	0.669023907	yes	up
ENSSSCG00000023548	GSTCD	1.338919397	0.421069113	0.015352313	0.467825317	yes	up
ENSSSCG00000023557	CCRL2	0.69182244	-0.531526284	0.022465411	0.524686071	yes	down
ENSSSCG00000023585	SERINC2	0.71864918	-0.476640427	0.000120673	0.068714733	yes	down
ENSSSCG00000023611	TNXB	0.59457257	-0.750075186	0.005616006	0.359764441	yes	down
ENSSSCG00000023630	CPM	0.696900795	-0.520974795	0.022619256	0.524686071	yes	down
ENSSSCG00000023666	CTSB	0.702429314	-0.509575042	0.000128005	0.068714733	yes	down
ENSSSCG00000023806	LRRN1	4.228581979	2.080173948	0.031670055	1	yes	up
ENSSSCG00000023852	CCL17	0.209326741	-2.256171468	0.03309609	0.622377939	yes	down
ENSSSCG00000023894	AKNA	0.706112665	-0.502029701	0.014033651	0.456766144	yes	down
ENSSSCG00000023915	SLC2A4	0.366430178	-1.448389772	0.006344219	0.366967563	yes	down
ENSSSCG00000023955	RAD51C	1.3829076	0.467704765	0.034452382	0.628078963	yes	up

ENSSSCG00000024043	ADAMTS2	0.764221755	-0.387936768	0.018783898	0.504122241	yes	down
ENSSSCG00000024045	CASTOR2	0.713239118	-0.487542264	0.023450525	0.532678954	yes	down
ENSSSCG00000024052	GPX4	0.794504123	-0.331873389	0.002000985	0.228866078	yes	down
ENSSSCG00000024108	SLC43A2	0.641560385	-0.640343034	0.008472398	0.389033038	yes	down
ENSSSCG00000024131	MINDY2	1.292676229	0.370360975	0.021948706	0.52018032	yes	up
ENSSSCG00000024300	TMEM254	1.339089157	0.421252019	0.014414855	0.457630538	yes	up
ENSSSCG00000024429	SIDT1	1.45042065	0.536471371	0.029971174	0.598270325	yes	up
ENSSSCG00000024480	SFMBT2	0.713077787	-0.487868632	0.033633423	0.626076307	yes	down
ENSSSCG00000024495	CD162	0.585279525	-0.772802285	0.010354561	0.417658959	yes	down
ENSSSCG00000024549	LYL1	0.746945183	-0.420925725	0.021758893	0.519816541	yes	down
ENSSSCG00000024562	CCR6	0.533571574	-0.906246285	0.025645901	0.5583584	yes	down
ENSSSCG00000024610	KRT4	3.618977323	1.855582068	0.033863068	0.626076307	yes	up
ENSSSCG00000024663	SPICE1	1.248403019	0.320083751	0.031690185	0.612165162	yes	up
ENSSSCG00000024780	ZFAND1	0.682291512	-0.551539826	0.043788963	0.678952552	yes	down
ENSSSCG00000024784	CACNA1S	0.101696512	-3.297657898	0.01943243	1	yes	down
ENSSSCG00000024881	TCP11L2	0.772129983	-0.373084359	0.017188421	0.491886525	yes	down
ENSSSCG00000025005	B4GALT6	0.489165557	-1.03160527	0.012344204	0.442351644	yes	down
ENSSSCG00000025034	LAPTM5	0.650690534	-0.619956528	3.67E-05	0.043794099	yes	down
ENSSSCG00000025042	ICOS	0.625531074	-0.676846541	0.020296854	0.509931129	yes	down
ENSSSCG00000025108	G6PD	0.76849802	-0.379886553	0.007999686	0.387495665	yes	down
ENSSSCG00000025114	FMNL3	0.762486527	-0.39121625	0.018194979	0.497279781	yes	down
ENSSSCG00000025133	ITGB2	0.620475237	-0.688554461	0.011756937	0.436740665	yes	down
ENSSSCG00000025188	LEPR	0.215490232	-2.214305623	0.046022195	1	yes	down
ENSSSCG00000025243	SGIP1	1.54263135	0.625393335	0.02550898	0.558288932	yes	up
ENSSSCG00000025401	CDK10	1.202707535	0.266285862	0.01026612	0.417658959	yes	up
ENSSSCG00000025485	—	0.31812636	-1.652328178	0.03935008	0.659812673	yes	down
ENSSSCG00000025499	FXYD5	0.743834867	-0.426945719	0.012000974	0.439315508	yes	down
ENSSSCG00000025561	VASN	0.659178188	-0.601259591	0.009848206	0.413744596	yes	down
ENSSSCG00000025592	TP63	0.450626095	-1.149997236	0.042926509	0.676981304	yes	down
ENSSSCG00000025602	DOCK4	0.655265119	-0.609849359	0.000163376	0.068714733	yes	down
ENSSSCG00000025698	SERPINE1	0.565269018	-0.822990468	0.013106779	0.448599721	yes	down
ENSSSCG00000025711	MEIOB	1.362026933	0.445755232	0.043338554	0.678952552	yes	up
ENSSSCG00000025717	HIC1	0.702971942	-0.508460988	0.023147882	0.52942603	yes	down
ENSSSCG00000025741	SNX20	0.765112722	-0.386255784	0.018360335	0.499431886	yes	down
ENSSSCG00000025795	CSF3R	0.530292111	-0.91514081	0.02376811	0.537082089	yes	down
ENSSSCG00000025822	SFRP1	0.542463202	-0.882402819	0.006199519	0.366967563	yes	down
ENSSSCG00000025885	ITLN	5.475517734	2.452995384	0.016654603	0.48592959	yes	up
ENSSSCG00000025955	SSBP4	0.782629242	-0.353599078	0.035973215	0.634028076	yes	down
ENSSSCG00000025981	SLC38A5	0.806479957	-0.310289416	0.045455531	0.686312765	yes	down
ENSSSCG00000026068	GRHL1	2.874059905	1.523090133	0.023159495	0.52942603	yes	up
ENSSSCG00000026084	SLC49A3	0.762189619	-0.391778135	0.011405478	0.436740665	yes	down

ENSSSCG00000026146	GALNT5	1.510524218	0.595049315	0.013600072	0.449667171	yes	up
ENSSSCG00000026210	CD307	0.738240678	-0.437836861	0.049009599	0.695594658	yes	down
ENSSSCG00000026297	KLB	1.708096304	0.772389318	0.000871147	0.158122669	yes	up
ENSSSCG00000026547	SLC45A3	1.268802591	0.343467623	0.046807674	0.691288485	yes	up
ENSSSCG00000026653	PILR	0.5969277	-0.744371893	0.038397898	0.650310844	yes	down
ENSSSCG00000026733	HIPK2	0.787018666	-0.345530242	0.024254012	0.543647983	yes	down
ENSSSCG00000026996	ABCC4	0.829964157	-0.268879062	0.047299547	0.691288485	yes	down
ENSSSCG00000027070	ENTPD6	1.38709756	0.472069262	0.001243442	0.197754624	yes	up
ENSSSCG00000027076	ZFTRAF1	1.291519564	0.369069497	0.027692086	0.573734675	yes	up
ENSSSCG00000027124	—	0.683307883	-0.549392325	0.001227449	0.197088207	yes	down
ENSSSCG00000027160	DSG3	0.36443623	-1.456261703	0.02513794	0.554956872	yes	down
ENSSSCG00000027232	—	5.125069144	2.357571469	0.005584462	0.359764441	yes	up
ENSSSCG00000027299	PANK4	1.29972434	0.378205673	0.008949874	0.394634056	yes	up
ENSSSCG00000027348	WIPF	0.750677135	-0.413735555	0.010325926	0.417658959	yes	down
ENSSSCG00000027357	CSTB	0.822105929	-0.282603797	0.025501526	0.558288932	yes	down
ENSSSCG00000027387	MS4A12	0.714644605	-0.484702132	0.017301516	0.492991747	yes	down
ENSSSCG00000027410	CBFA2T2	1.272866765	0.348081416	0.005520208	0.358143884	yes	up
ENSSSCG00000027486	TRIP6	0.321918127	-1.635234279	0.000636866	0.145734681	yes	down
ENSSSCG00000027487	LAT2	0.75604326	-0.403459308	0.002520983	0.258269334	yes	down
ENSSSCG00000027557	CDC14	1.248446692	0.320134221	0.028228718	0.581964637	yes	up
ENSSSCG00000027568	BLK	0.437293696	-1.193325544	0.001377071	0.198589146	yes	down
ENSSSCG00000027628	IL6R	0.672769583	-0.571815615	0.016392256	0.484426375	yes	down
ENSSSCG00000027796	SLC22A7	0.17895016	-2.48237026	0.014235781	1	yes	down
ENSSSCG00000027826	GIMAP4	0.65828984	-0.603205164	0.024482812	0.54657551	yes	down
ENSSSCG00000028104	HOXC11	0.560225304	-0.835920946	0.021530863	0.518074755	yes	down
ENSSSCG00000028157	CASP8	0.783034544	-0.352852141	0.019476297	0.504239817	yes	down
ENSSSCG00000028210	NT5C	0.81042256	-0.303253759	0.015288922	0.467600207	yes	down
ENSSSCG00000028331	IL1R2	0.371095806	-1.430136397	0.010557339	0.421763186	yes	down
ENSSSCG00000028461	CD172	0.685517056	-0.544735534	0.016864625	0.486305882	yes	down
ENSSSCG00000028501	ALDH3	1.750094544	0.807432862	0.01473395	0.46161769	yes	up
ENSSSCG00000028523	HUS1	1.219295582	0.286047907	0.042378856	0.671710115	yes	up
ENSSSCG00000028571	SLX9	1.373032322	0.457365588	0.0005384	0.142710178	yes	up
ENSSSCG00000028593	TEPSIN	1.311896261	0.391653643	0.031014037	0.607254547	yes	up
ENSSSCG00000028944	TPCN3	2.425435	1.278243517	0.048182221	0.691288485	yes	up
ENSSSCG00000028996	ALDH1A1	0.685440009	-0.544897691	0.001802644	0.215227738	yes	down
ENSSSCG00000029037	DRD1	1.226894177	0.295010818	0.008201168	0.387495665	yes	up
ENSSSCG00000029088	MTG1	1.263769988	0.33773391	0.016732111	0.48592959	yes	up
ENSSSCG00000029163	BCAT1	0.613499993	-0.704864768	0.009422022	0.400352029	yes	down
ENSSSCG00000029239	MZB1	0.563770423	-0.826820303	0.003670668	0.296453856	yes	down
ENSSSCG00000029267	TIGIT	0.645539554	-0.631422598	0.039855613	0.662872245	yes	down
ENSSSCG00000029291	PIGZ	0.713978772	-0.486046915	0.007195911	0.381474671	yes	down

ENSSSCG00000029474	AVEN	1.313595131	0.393520685	0.019713043	0.504766715	yes	up
ENSSSCG00000029484	EMB	0.647591236	-0.626844634	0.000405303	0.11873958	yes	down
ENSSSCG00000029621	BMPR1B	4.075972906	2.027144461	0.036144582	0.635345659	yes	up
ENSSSCG00000029753	CYTIP	0.701877631	-0.510708568	0.016890677	0.486305882	yes	down
ENSSSCG00000029886	LYVE1	0.767643491	-0.381491644	0.048212446	0.691288485	yes	down
ENSSSCG00000030013	LCT	0.290737906	-1.782208914	0.034189348	0.627268647	yes	down
ENSSSCG00000030048	PLEKHG2	0.783863151	-0.351326288	0.029320684	0.593091889	yes	down
ENSSSCG00000030140	AMY	1.303460147	0.382346474	0.008398869	0.389033038	yes	up
ENSSSCG00000030156	APBB1IP	0.623384342	-0.681806176	0.012955697	0.448536088	yes	down
ENSSSCG00000030172	CD40LG	0.475440469	-1.072663387	0.012432366	0.443569791	yes	down
ENSSSCG00000030209	MFNG	0.727039511	-0.459894324	0.008058168	0.387495665	yes	down
ENSSSCG00000030241	—	0.653345086	-0.614082895	6.51E-05	0.060359762	yes	down
ENSSSCG00000030277	ENDOV	1.392346266	0.477518043	0.007590331	0.387495665	yes	up
ENSSSCG00000030326	TCP11L1	0.71208593	-0.489876747	9.81E-05	0.065519059	yes	down
ENSSSCG00000030358	—	0.731411368	-0.451245045	0.008947212	0.394634056	yes	down
ENSSSCG00000030432	RAB42	0.662946803	-0.593034987	0.0426306	0.67382328	yes	down
ENSSSCG00000030460	ARHGEF6	0.796174168	-0.32884403	0.014206419	0.456766144	yes	down
ENSSSCG00000030502	CEP170	0.779518933	-0.359344032	0.012255997	0.442351644	yes	down
ENSSSCG00000030585	HOXC6	1.59636322	0.674788945	0.007478822	0.385459407	yes	up
ENSSSCG00000030680	TCF7	0.668865816	-0.580211281	0.042483055	0.672440312	yes	down
ENSSSCG00000030681	MYBPH	0.383325717	-1.383357306	0.035699931	0.633787122	yes	down
ENSSSCG00000030827	FGFR3	1.319565758	0.400063247	0.003177708	0.288459657	yes	up
ENSSSCG00000030879	MS4A8	0.386741907	-1.370556993	0.012163496	0.441694027	yes	down
ENSSSCG00000030998	WIF1	2.859406299	1.51571563	0.043603547	0.678952552	yes	up
ENSSSCG00000031037	—	0.281415616	-1.829225707	0.02720025	0.571342115	yes	down
ENSSSCG00000031085	—	0.206941536	-2.27270485	0.009975819	1	yes	down
ENSSSCG00000031111	CBR1	4.135280874	2.047985323	0.021134423	0.513921244	yes	up
ENSSSCG00000031118	PREX1	0.695145537	-0.52461304	0.020306857	0.509931129	yes	down
ENSSSCG00000031144	RAMP3	0.696894102	-0.52098865	0.013569212	0.449667171	yes	down
ENSSSCG00000031155	RASD2	0.540032318	-0.888882348	0.008549921	0.389033038	yes	down
ENSSSCG00000031170	—	0.809709335	-0.304523985	0.031803598	0.612165162	yes	down
ENSSSCG00000031324	ZYG11A	0.079993036	-3.643981788	0.043076079	1	yes	down
ENSSSCG00000031349	ZNF580	0.790722957	-0.338755784	0.026714704	0.570471672	yes	down
ENSSSCG00000031361	CELSR1	0.684684061	-0.546489667	0.022563455	0.524686071	yes	down
ENSSSCG00000031465	PRRT1B	1.283796756	0.36041682	0.00803926	0.387495665	yes	up
ENSSSCG00000031476	KLHL6	0.768393294	-0.380083167	0.031447312	0.611936786	yes	down
ENSSSCG00000031487	LSP1	0.586360399	-0.770140425	0.002069703	0.233526814	yes	down
ENSSSCG00000031492	PPP1R18	0.778096867	-0.361978324	0.027037018	0.571323123	yes	down
ENSSSCG00000031694	DIRAS2	0.093870121	-3.413190177	0.029290821	1	yes	down
ENSSSCG00000031819	TP53I11	0.617316828	-0.695916975	9.54E-07	0.002608747	yes	down
ENSSSCG00000031849	NAP1L2	0.45773471	-1.1274164	0.01886798	0.504122241	yes	down

ENSSSCG00000031866	TIMP3	0.7866587	-0.346190252	0.041175298	0.667123021	yes	down
ENSSSCG00000031897	—	0.56721829	-0.818024041	0.001610453	0.209336848	yes	down
ENSSSCG00000031898	CALHM5	0.777188131	-0.363664226	0.032875361	0.621426271	yes	down
ENSSSCG00000031924	NKX3-1	1.691439989	0.758251992	0.016997886	0.487710813	yes	up
ENSSSCG00000031970	RASSF5	0.643549573	-0.63587681	0.003710333	0.296453856	yes	down
ENSSSCG00000032003	GST	0.796095298	-0.328986953	0.021355759	0.516840311	yes	down
ENSSSCG00000032019	DNASE1L3	0.402790573	-1.311898175	0.041320509	0.667123021	yes	down
ENSSSCG00000032084	CCDC102	2.688652288	1.42688319	0.003606712	0.296453856	yes	up
ENSSSCG00000032108	SH2D1B	0.39964085	-1.323224036	0.003539882	0.296453856	yes	down
ENSSSCG00000032124	GABRR3	14.77727909	3.885308748	0.023990452	1	yes	up
ENSSSCG00000032160	FNDC7	0.149590358	-2.740910906	0.049496579	1	yes	down
ENSSSCG00000032162	P2RY13	0.558152657	-0.841268336	0.047941172	0.691288485	yes	down
ENSSSCG00000032341	AIF1	0.60800524	-0.717844336	0.003419556	0.296453856	yes	down
ENSSSCG00000032374	SULT1B1	1.267403223	0.341875589	0.00021087	0.081891134	yes	up
ENSSSCG00000032419	—	0.295388142	-1.759316182	0.002201745	0.238317669	yes	down
ENSSSCG00000032428	ARSA	0.75267847	-0.40989439	0.013499185	0.449667171	yes	down
ENSSSCG00000032525	RAET1	0.514867521	-0.957726831	0.001683365	0.211347945	yes	down
ENSSSCG00000032536	B3GNT8	0.738232509	-0.437852825	0.046831015	0.691288485	yes	down
ENSSSCG00000032561	PDCD1LG2	0.534231487	-0.904463085	0.021609609	0.51847537	yes	down
ENSSSCG00000032578	TNFRSF17	0.653979902	-0.612681796	0.007381737	0.385211341	yes	down
ENSSSCG00000032599	—	1.445025029	0.531094481	0.005167125	0.346159768	yes	up
ENSSSCG00000032601	RASSF2	0.669549628	-0.5787371	0.002188453	0.238317669	yes	down
ENSSSCG00000032686	RUNX3	0.582270568	-0.780238398	0.005397799	0.352100944	yes	down
ENSSSCG00000032698	TPPP	0.686021031	-0.54367529	0.001311646	0.198589146	yes	down
ENSSSCG00000032721	TRAF3IP3	0.68385342	-0.548240971	0.025636372	0.5583584	yes	down
ENSSSCG00000032796	GRINA	0.761527001	-0.393032904	0.019439173	0.504239817	yes	down
ENSSSCG00000032803	CYP2S1	0.818848809	-0.288330996	0.044221283	0.679567425	yes	down
ENSSSCG00000032956	BMERB1	0.628394733	-0.670257007	0.007738314	0.387495665	yes	down
ENSSSCG00000032964	STK11IP	1.265603324	0.339825294	0.028490531	0.58539213	yes	up
ENSSSCG00000033003	WFDC2	0.673357424	-0.570555592	0.037283329	0.647166387	yes	down
ENSSSCG00000033009	GCNT2	0.795188802	-0.330630655	0.030878803	0.607123214	yes	down
ENSSSCG00000033015	B3GNT7	2.288795662	1.194588669	0.026831488	0.571323123	yes	up
ENSSSCG00000033037	SLC25A28_37	1.341368327	0.423705442	0.013548012	0.449667171	yes	up
ENSSSCG00000033116	YBEY	1.48601173	0.571445504	0.030704686	0.606072765	yes	up
ENSSSCG00000033183	—	0.315452474	-1.664505429	0.044600982	0.679681619	yes	down
ENSSSCG00000033189	—	1.200559637	0.26370707	0.02479511	0.549871908	yes	up
ENSSSCG00000033222	TRIM14	1.239123244	0.309319686	0.005673519	0.359764441	yes	up
ENSSSCG00000033262	CD74	0.562679282	-0.829615252	0.000148504	0.068714733	yes	down
ENSSSCG00000033266	C7orf57	1.752245717	0.809205098	0.009717084	0.4118416	yes	up
ENSSSCG00000033297	AP5S1	1.208275947	0.272949976	0.031763979	0.612165162	yes	up
ENSSSCG00000033335	AKIP1	1.252590549	0.324914899	0.016956223	0.487352774	yes	up

ENSSSCG00000033337	ARHGDIB	0.631004688	-0.66427737	0.000110303	0.068003035	yes	down
ENSSSCG00000033381	IRF4	0.590575823	-0.759805799	0.012167151	0.441694027	yes	down
ENSSSCG00000033382	—	0.030948553	-5.013984214	0.000154361	0.068714733	yes	down
ENSSSCG00000033452	—	0.448218744	-1.157725112	0.041299548	0.667123021	yes	down
ENSSSCG00000033512	C1QB	0.579306598	-0.787600999	0.008283231	0.387495665	yes	down
ENSSSCG00000033558	GABRB2	1.471731468	0.557514462	0.014700054	0.46161769	yes	up
ENSSSCG00000033585	DUSP23	1.56108931	0.642553077	0.000228936	0.082219172	yes	up
ENSSSCG00000033586	DBNDD2	1.200312988	0.263410646	0.008275507	0.387495665	yes	up
ENSSSCG00000033592	TMC8	0.631954708	-0.66210693	0.003826568	0.302842922	yes	down
ENSSSCG00000033623	—	0.571887218	-0.806197434	0.001868856	0.215227738	yes	down
ENSSSCG00000033691	RAB26	1.482550685	0.568081428	0.015068578	0.467600207	yes	up
ENSSSCG00000033721	TRAV	0.688488982	-0.538494529	0.037564081	0.647166387	yes	down
ENSSSCG00000033849	CD37	0.712757985	-0.488515799	0.041090704	0.667123021	yes	down
ENSSSCG00000033880	TSPAN32	0.390462075	-1.356745668	0.003432539	0.296453856	yes	down
ENSSSCG00000033909	—	0.668068526	-0.581932003	0.010261421	0.417658959	yes	down
ENSSSCG00000033937	DYNC2H1	0.664583947	-0.589476651	0.011675558	0.436740665	yes	down
ENSSSCG00000033945	RGS18	0.609893469	-0.713370829	0.002706446	0.269017486	yes	down
ENSSSCG00000034074	ERI2	1.333277218	0.41497678	0.019469907	0.504239817	yes	up
ENSSSCG00000034102	DGAT2	1.894851141	0.922084515	0.005182343	0.346159768	yes	up
ENSSSCG00000034220	CES1	0.174721172	-2.516873652	2.13E-10	1.19E-06	yes	down
ENSSSCG00000034262	FIGN	1.769180906	0.823081577	0.04090893	0.667123021	yes	up
ENSSSCG00000034284	CD305_6	0.612707192	-0.706730308	0.017872659	0.492991747	yes	down
ENSSSCG00000034293	ARL4C	0.678278382	-0.560050582	0.014496043	0.459334774	yes	down
ENSSSCG00000034297	HES2	1.812794008	0.858214997	0.004454969	0.326473146	yes	up
ENSSSCG00000034378	IFNGR2	0.79749737	-0.326448333	0.046640215	0.691078036	yes	down
ENSSSCG00000034515	CD200	1.236640542	0.306426209	0.002576486	0.259185208	yes	up
ENSSSCG00000034555	CLECL1	0.684320697	-0.547255513	0.038774203	0.654030728	yes	down
ENSSSCG00000034615	TNFAIP8L1	0.771040091	-0.375122218	0.008713964	0.390119267	yes	down
ENSSSCG00000034746	CAPN8	1.91582046	0.937962366	0.00726717	0.38403314	yes	up
ENSSSCG00000034752	SUPT4H1	0.726546287	-0.460873382	0.001610204	0.209336848	yes	down
ENSSSCG00000034846	—	1.47040717	0.556215707	0.00772251	0.387495665	yes	up
ENSSSCG00000034914	CD163	0.206844043	-2.273384689	0.006598493	0.367294106	yes	down
ENSSSCG00000034937	SH3BGRL3	0.824846496	-0.277802437	0.005797709	0.361768194	yes	down
ENSSSCG00000034980	IRF8	0.722244823	-0.469440138	0.027440951	0.572788782	yes	down
ENSSSCG00000035012	ELOVL7	1.370192006	0.454378073	0.009282748	0.398489984	yes	up
ENSSSCG00000035073	ABCB1	0.014058743	-6.152388575	0.042396698	0.671710115	yes	down
ENSSSCG00000035081	CCDC106	0.593871894	-0.751776338	0.015643826	0.471545583	yes	down
ENSSSCG00000035102	LYPD1	2.224360062	1.153390339	0.022264493	0.522918108	yes	up
ENSSSCG00000035170	VSIG4	0.588372324	-0.765198708	0.041832623	0.667913669	yes	down
ENSSSCG00000035186	ADCYAP1	1.553438296	0.635464937	0.043478811	0.678952552	yes	up
ENSSSCG00000035218	ADA2	0.414654985	-1.270016659	0.00099023	0.172248434	yes	down

ENSSSCG00000035224	ABI3	0.75917691	-0.397491981	0.022206435	0.522737311	yes	down
ENSSSCG00000035249	GADD45G	0.653480887	-0.613783055	0.01018287	0.416773906	yes	down
ENSSSCG00000035256	SPN	0.609363776	-0.714624354	0.010002848	0.414485269	yes	down
ENSSSCG00000035284	BMF	0.700938905	-0.512639393	0.003641999	0.296453856	yes	down
ENSSSCG00000035337	—	0.597485646	-0.74302404	0.048148282	0.691288485	yes	down
ENSSSCG00000035443	—	0.274294824	-1.866200696	0.048711822	1	yes	down
ENSSSCG00000035489	IFTAP	1.281003366	0.357274266	0.041135329	0.667123021	yes	up
ENSSSCG00000035537	RUNX1	0.808629061	-0.306450043	0.032228429	0.614363628	yes	down
ENSSSCG00000035594	PRSS22	1.349761108	0.43270409	0.043345788	0.678952552	yes	up
ENSSSCG00000035617	—	0.203144387	-2.299422592	1.25E-06	0.002608747	yes	down
ENSSSCG00000035657	DIP2A	1.265342414	0.339527845	0.011965194	0.439315508	yes	up
ENSSSCG00000035669	AHNAK	1.719247672	0.781777392	0.007170726	0.381350169	yes	up
ENSSSCG00000035696	MCEE	1.205206118	0.269279902	0.049611334	0.696771975	yes	up
ENSSSCG00000035940	SPSB1	0.702101568	-0.510248345	0.019076587	0.504239817	yes	down
ENSSSCG00000035983	TPPP3	0.601706065	-0.732869195	0.043572248	0.678952552	yes	down
ENSSSCG00000035999	NUDT16L1	1.233507132	0.302766057	0.034289397	0.627268647	yes	up
ENSSSCG00000036078	KCNJ3	6.048413033	2.596556661	0.037313431	1	yes	up
ENSSSCG00000036113	GSAP	0.759226144	-0.397398423	0.039472595	0.659812673	yes	down
ENSSSCG00000036118	UGT	0.532375037	-0.90948517	0.025748397	0.559860005	yes	down
ENSSSCG00000036135	COL1A1	0.774802757	-0.368099007	0.029214056	0.593091889	yes	down
ENSSSCG00000036223	ACKR1	0.648791848	-0.624172402	0.01517974	0.467600207	yes	down
ENSSSCG00000036224	—	0.572123368	-0.805601824	0.011343703	0.436147095	yes	down
ENSSSCG00000036233	—	0.321548646	-1.636891082	0.024540611	0.547134398	yes	down
ENSSSCG00000036246	GP91-PHOX	0.503698704	-0.989367077	0.037649432	0.647166387	yes	down
ENSSSCG00000036482	ZSWIM7	1.358566702	0.4420854	0.000663455	0.145734681	yes	up
ENSSSCG00000036556	IFNAR2	0.740396358	-0.433630296	0.002675573	0.267541231	yes	down
ENSSSCG00000036573	SPDEF	1.343815185	0.426334738	0.019829149	0.504766715	yes	up
ENSSSCG00000036612	TNFAIP2	0.653496969	-0.61374755	0.03183579	0.612165162	yes	down
ENSSSCG00000036618	FCGR3	0.656753834	-0.606575377	0.01422351	0.456766144	yes	down
ENSSSCG00000036673	PPT1	0.831999043	-0.265346226	0.003031756	0.287655106	yes	down
ENSSSCG00000036747	WAS	0.623868877	-0.680685256	0.004001867	0.312276519	yes	down
ENSSSCG00000036755	TENT5B	0.710430248	-0.493235086	0.026084134	0.563539972	yes	down
ENSSSCG00000036844	GPR171	0.615913622	-0.699200058	0.001592748	0.209336848	yes	down
ENSSSCG00000036865	SDS	0.375096432	-1.414666556	0.014999395	0.467600207	yes	down
ENSSSCG00000036884	—	0.120872627	-3.048440532	0.018244883	1	yes	down
ENSSSCG00000036963	LRRC25	0.642881908	-0.637374343	0.009241342	0.398489984	yes	down
ENSSSCG00000037016	ID1	1.22210482	0.289368031	0.043576671	0.678952552	yes	up
ENSSSCG00000037019	CAMK4	0.608036034	-0.71777127	0.01623776	0.483087156	yes	down
ENSSSCG00000037071	—	0.677681137	-0.56132148	0.048088743	0.691288485	yes	down
ENSSSCG00000037132	POU2F2	0.6583778	-0.603012405	0.008545421	0.389033038	yes	down
ENSSSCG00000037153	FEM1A	1.218909165	0.285590618	0.020869737	0.513921244	yes	up

ENSSSCG00000037264	RAB31	0.784777228	-0.349644916	0.026321939	0.566430493	yes	down
ENSSSCG00000037269	LANCL3	1.592161058	0.670986281	0.015469764	0.468837737	yes	up
ENSSSCG00000037272	FABP2	0.254024491	-1.976960498	0.03482164	0.632050612	yes	down
ENSSSCG00000037318	TRABD2B	1.498953912	0.583956026	0.002288788	0.241901682	yes	up
ENSSSCG00000037360	CST3	0.717285133	-0.479381365	0.04423557	0.679567425	yes	down
ENSSSCG00000037425	NFAM1	0.40267156	-1.312324515	0.00186102	0.215227738	yes	down
ENSSSCG00000037450	SSC5D	0.813217686	-0.298286503	0.041869581	0.667913669	yes	down
ENSSSCG00000037466	SIGLEC5	0.485586881	-1.042198649	0.004494593	0.326473146	yes	down
ENSSSCG00000037468	GNE	1.300040306	0.378556353	0.008925404	0.394634056	yes	up
ENSSSCG00000037561	ATP11A	1.238745645	0.308879985	0.04746726	0.691288485	yes	up
ENSSSCG00000037562	SLC2A4RG	1.264677116	0.338769098	0.044869958	0.680188307	yes	up
ENSSSCG00000037775	IGHM	0.604270285	-0.726734096	0.009126495	0.396883703	yes	down
ENSSSCG00000037987	MS4A10	0.187234262	-2.417083635	0.011454507	1	yes	down
ENSSSCG00000038055	CORO1A	0.669763458	-0.578276429	0.025439358	0.558228445	yes	down
ENSSSCG00000038062	SAMSN1	0.57202683	-0.805845279	0.010672043	0.42532802	yes	down
ENSSSCG00000038110	TIFAB	0.469310926	-1.091384047	0.004530798	0.326473146	yes	down
ENSSSCG00000038128	CDYL2	1.436794018	0.522853248	0.041019968	0.667123021	yes	up
ENSSSCG00000038204	—	0.233397745	-2.099137473	0.000586491	0.145734681	yes	down
ENSSSCG00000038322	DGAT2	2.73106102	1.449461549	0.001066789	0.181778619	yes	up
ENSSSCG00000038345	IKZF1	0.569877549	-0.811276138	0.004187591	0.323023561	yes	down
ENSSSCG00000038383	DYNC2LI1	1.756436857	0.812651714	0.0091658	0.397557662	yes	up
ENSSSCG00000038420	PERP	1.232737308	0.301865399	0.026579981	0.569054167	yes	up
ENSSSCG00000038439	PRX	0.680322357	-0.555709594	0.023480659	0.532678954	yes	down
ENSSSCG00000038462	CEP70	2.578092454	1.366304002	0.018865858	0.504122241	yes	up
ENSSSCG00000038500	TRIB1	0.800932546	-0.320247351	0.040033645	0.66360476	yes	down
ENSSSCG00000038610	INHBB	0.652407662	-0.61615437	0.015288067	0.467600207	yes	down
ENSSSCG00000038631	TCFL5	1.294169326	0.372026389	0.047807397	0.691288485	yes	up
ENSSSCG00000038706	C1QC	0.563318094	-0.827978282	0.00922933	0.398489984	yes	down
ENSSSCG00000038713	CRIP2	0.797874667	-0.325765954	0.035783988	0.633787122	yes	down
ENSSSCG00000038719	IGLL1	0.605451538	-0.723916609	0.035862314	0.633787122	yes	down
ENSSSCG00000038732	AGAP2	0.668780298	-0.580395747	0.01877868	0.504122241	yes	down
ENSSSCG00000038890	SMIM31	1.681595893	0.749831051	0.01227681	0.442351644	yes	up
ENSSSCG00000038918	CTSF	0.711434978	-0.491196189	0.002398391	0.25031708	yes	down
ENSSSCG00000038940	GNPDA1	1.387766878	0.472765239	0.000589398	0.145734681	yes	up
ENSSSCG00000038953	UBE2B	0.827022554	-0.274001422	0.003078607	0.288459657	yes	down
ENSSSCG00000039025	FREM1	2.304178601	1.204252547	0.023252557	0.529733208	yes	up
ENSSSCG00000039052	ZNF397	1.314729748	0.394766274	0.012841829	0.447694574	yes	up
ENSSSCG00000039056	GAS7	0.743047811	-0.428473053	0.010555934	0.421763186	yes	down
ENSSSCG00000039057	ALKAL2	0.499746183	-1.000732548	0.012457886	0.443569791	yes	down
ENSSSCG00000039214	IL1B	0.408190454	-1.292685652	0.006526156	0.366967563	yes	down
ENSSSCG00000039222	BASP1	0.750563033	-0.413954859	0.011474539	0.436740665	yes	down

ENSSSCG00000039243	CD81	0.790589263	-0.338999734	0.006442246	0.366967563	yes	down
ENSSSCG00000039261	WSCD2	1.71946366	0.781958626	0.0155175	0.469432486	yes	up
ENSSSCG00000039276	UGT2B31	0.53400547	-0.905073574	0.000560818	0.144078411	yes	down
ENSSSCG00000039405	TNFRSF17	8.658807645	3.114168373	0.016489801	1	yes	up
ENSSSCG00000039416	CXCR4	0.626338274	-0.674986055	0.000114024	0.068003035	yes	down
ENSSSCG00000039480	ERLIN2	1.208745727	0.273510789	0.031363458	0.611129971	yes	up
ENSSSCG00000039508	REC114	3.266861007	1.707905075	0.008284086	0.387495665	yes	up
ENSSSCG00000039542	—	0.665608231	-0.58725482	0.000317075	0.098438311	yes	down
ENSSSCG00000039644	TRPM5	1.60549004	0.683013715	0.030940904	0.607147067	yes	up
ENSSSCG00000039656	RHOG	0.821600313	-0.283491364	0.0021846	0.238317669	yes	down
ENSSSCG00000039715	FAHD1	1.92227266	0.942812986	0.035882254	0.633787122	yes	up
ENSSSCG00000039731	GLRX2	0.699543372	-0.515514587	0.011621604	0.436740665	yes	down
ENSSSCG00000039741	—	0.25559533	-1.968066617	0.021864361	0.52018032	yes	down
ENSSSCG00000039756	FOXC1	0.225370814	-2.1496274	0.001847817	0.215227738	yes	down
ENSSSCG00000039763	CMBL	1.362592144	0.446353794	0.003209968	0.289747326	yes	up
ENSSSCG00000039770	SLC6A9	0.672797577	-0.571755585	0.011872697	0.438633129	yes	down
ENSSSCG00000039779	CHGB	1.543701166	0.626393498	0.047339099	0.691288485	yes	up
ENSSSCG00000039815	—	0.685482179	-0.544808934	0.029884649	0.598270325	yes	down
ENSSSCG00000039821	—	0.637085025	-0.650442169	0.044389167	0.679681619	yes	down
ENSSSCG00000039874	CCL26	0.152710545	-2.711128406	0.045588437	1	yes	down
ENSSSCG00000039880	—	0.370588111	-1.432111497	0.009274469	0.398489984	yes	down
ENSSSCG00000039888	STXBP5L	2.834996341	1.503346873	0.006190653	0.366967563	yes	up
ENSSSCG00000039914	ZNF470	1.541006357	0.623872813	0.02835645	0.583877135	yes	up
ENSSSCG00000039921	AMY	3.880605687	1.956281847	0.03793908	0.647166387	yes	up
ENSSSCG00000039985	CES1	0.555342446	-0.848550427	0.017350193	0.492991747	yes	down
ENSSSCG00000039997	ZNF41	1.262410342	0.33618093	0.039367207	0.659812673	yes	up
ENSSSCG00000040010	BCL2A1	0.586297996	-0.77029397	0.048722539	0.69421304	yes	down
ENSSSCG00000040035	MUC2	1.368196121	0.452275044	0.011152708	0.434053184	yes	up
ENSSSCG00000040140	CD3E	0.612456097	-0.707321666	0.002543367	0.258973707	yes	down
ENSSSCG00000040147	SHISA3	0.491559026	-1.024563429	0.021487328	0.518074755	yes	down
ENSSSCG00000040182	RHBDD2	0.712592475	-0.488850846	0.000611323	0.145734681	yes	down
ENSSSCG00000040275	HEBP1	0.652715012	-0.615474874	0.007460189	0.385459407	yes	down
ENSSSCG00000040293	MRGPRX	2.875872316	1.523999624	0.00312122	0.288459657	yes	up
ENSSSCG00000040296	EHHADH	1.233105384	0.302296101	0.048074737	0.691288485	yes	up
ENSSSCG00000040507	PDLIM7	0.782991069	-0.352932243	0.020570356	0.511065793	yes	down
ENSSSCG00000040509	LIMD2	0.662919663	-0.59309405	0.039030416	0.657025111	yes	down
ENSSSCG00000040524	ISYNA1	0.708410511	-0.497342477	0.010332769	0.417658959	yes	down
ENSSSCG00000040535	LY6E	0.516956471	-0.951885288	0.000443042	0.123306032	yes	down
ENSSSCG00000040638	DIO2	1.915841809	0.937978442	0.019231179	0.504239817	yes	up
ENSSSCG00000040681	FABP4	2.045102137	1.032172896	0.012819524	0.447694574	yes	up
ENSSSCG00000040687	CDH8	10.35461691	3.372202273	0.026827924	1	yes	up

ENSSSCG00000040688	KRAB	1.392987241	0.478182044	0.016546355	0.484750143	yes	up
ENSSSCG00000040689	APOA4	0.333317823	-1.585029634	0.038679446	0.65375311	yes	down
ENSSSCG00000040698	PRR7	0.727850086	-0.458286763	0.031725958	0.612165162	yes	down
ENSSSCG00000040707	RASEF	1.316960192	0.397211737	0.043428794	0.678952552	yes	up
ENSSSCG00000040711	STRA6	3.010347155	1.589929869	0.000233123	0.082219172	yes	up
ENSSSCG00000040793	CTSD	0.678089045	-0.560453359	0.003144785	0.288459657	yes	down
ENSSSCG00000040824	CNTN2	0.376746063	-1.40833566	0.024728189	0.549117058	yes	down
ENSSSCG00000040849	COL3A	0.317112557	-1.65693309	0.016457654	0.484426375	yes	down
ENSSSCG00000040947	REG4	1.496450439	0.581544499	0.039373374	0.659812673	yes	up
ENSSSCG00000040977	SNX8	0.832346364	-0.264744094	0.020540968	0.511065793	yes	down
ENSSSCG00000040980	UGT	0.492804408	-1.020912935	0.000462705	0.126667354	yes	down
ENSSSCG00000040981	GMFG	0.578444712	-0.789749023	0.00026113	0.088992204	yes	down
ENSSSCG00000040986	—	0.206166466	-2.278118402	0.031881051	0.612165162	yes	down
ENSSSCG00000040998	LEAP2	0.75121511	-0.412702013	0.047188873	0.691288485	yes	down
ENSSSCG00000041169	—	2.927087607	1.549465926	0.013441465	0.449667171	yes	up
ENSSSCG00000041214	—	0.338607112	-1.562315821	0.007981145	0.387495665	yes	down
ENSSSCG00000041692	TRBV	0.266643868	-1.907013943	0.017673677	0.492991747	yes	down
ENSSSCG00000042026	—	1.251032785	0.323119597	0.019795175	0.504766715	yes	up
ENSSSCG00000042062	—	2.433685418	1.283142695	0.017463053	0.492991747	yes	up
ENSSSCG00000042226	CTSG	3.345501654	1.742222558	0.04170275	0.667913669	yes	up
ENSSSCG00000042466	MRGPRX	1.745962244	0.804022361	0.015770801	0.473662964	yes	up
ENSSSCG00000042589	—	0.100705254	-3.311789135	0.017092745	1	yes	down
ENSSSCG00000042820	—	11.23872973	3.490407077	0.022187175	1	yes	up
ENSSSCG00000043551	MRGPRX	0.498595195	-1.004059116	0.006548676	0.366967563	yes	down
ENSSSCG00000043562	—	0.663256287	-0.592361649	0.041494479	0.667549428	yes	down
ENSSSCG00000043814	—	3.264748969	1.706972065	0.013861592	0.454763701	yes	up
ENSSSCG00000043911	MRGPRX	4.505013132	2.171531311	0.019471169	0.504239817	yes	up
ENSSSCG00000044127	STARD9	2.475834745	1.307915022	0.048714208	0.69421304	yes	up
ENSSSCG00000044150	TRBV	0.643047811	-0.637002088	0.018287039	0.498979186	yes	down
ENSSSCG00000044690	FMN1	1.266524144	0.34087458	0.040744197	0.667123021	yes	up
ENSSSCG00000044713	—	0.206812246	-2.27360648	0.039415626	1	yes	down
ENSSSCG00000044736	—	0.657467866	-0.605007712	0.0445849	0.679681619	yes	down
ENSSSCG00000044752	—	1.89941329	0.925553853	0.003397977	0.296453856	yes	up
ENSSSCG00000045079	PARK2	0.50830977	-0.976220135	0.011228875	0.434053184	yes	down
ENSSSCG00000045225	—	0.387949118	-1.366060649	0.046351466	0.690193347	yes	down
ENSSSCG00000045267	—	1.974132104	0.981218535	0.041012709	0.667123021	yes	up
ENSSSCG00000045313	—	9.803845947	3.293347815	0.01741879	1	yes	up
ENSSSCG00000045343	—	2.133273008	1.093068608	0.041026824	0.667123021	yes	up
ENSSSCG00000045344	—	4.093701763	2.033406002	0.033172727	1	yes	up
ENSSSCG00000045405	KRAB	2.236852983	1.161470438	0.033384792	0.625693204	yes	up
ENSSSCG00000045414	—	0.491585735	-1.024485044	0.000360965	0.107638444	yes	down

ENSSSCG00000045502	WDFY4	0.684439483	-0.547005109	0.016236377	0.483087156	yes	down
ENSSSCG00000045950	C1orf54	0.56978636	-0.811507008	5.36E-05	0.05593289	yes	down
ENSSSCG00000045990	CCNB1IP1	3.181988738	1.669928729	0.022023324	0.52018032	yes	up
ENSSSCG00000046214	—	1.604902501	0.682485655	0.024665846	0.548462011	yes	up
ENSSSCG00000046319	IPCEF1	0.609020015	-0.715438454	0.008798419	0.392847057	yes	down
ENSSSCG00000046403	MRGPRX	0.435043549	-1.200768269	0.00634005	0.366967563	yes	down
ENSSSCG00000046490	CTSG	0.647246749	-0.627612281	0.049373572	0.696771975	yes	down
ENSSSCG00000046498	—	2.291707224	1.196422745	0.027681007	0.573734675	yes	up
ENSSSCG00000046754	KRAB	2.510599505	1.328031906	0.003617846	0.296453856	yes	up
ENSSSCG00000046958	—	0.233244396	-2.100085676	0.012814961	0.447694574	yes	down
ENSSSCG00000046978	—	0.159289764	-2.650274529	0.046600536	1	yes	down
ENSSSCG00000047060	CCER2	3.409874473	1.76971863	0.01421895	0.456766144	yes	up
ENSSSCG00000047187	—	0.24827569	-2.00998509	0.016635115	0.48592959	yes	down
ENSSSCG00000047753	KRAB	1.965985925	0.975252993	0.032160107	0.614363628	yes	up
ENSSSCG00000047799	—	0.37337704	-1.421294881	0.03609861	0.635206211	yes	down
ENSSSCG00000047897	—	0.530800787	-0.913757585	0.000712772	0.145734681	yes	down
ENSSSCG00000048168	KRAB	0.117033195	-3.09501031	0.026507318	1	yes	down
ENSSSCG00000048179	—	0.406920464	-1.297181259	0.043621321	0.678952552	yes	down
ENSSSCG00000048614	—	11.21197562	3.486968607	0.048987921	1	yes	up
ENSSSCG00000048650	—	9.068990249	3.180941929	0.029056022	1	yes	up
ENSSSCG00000049044	—	1.60684705	0.68423261	0.048227504	0.691288485	yes	up
ENSSSCG00000049172	PLA2G	0.665802147	-0.586834573	0.049611466	0.696771975	yes	down
ENSSSCG00000049683	—	0.164881967	-2.60049447	0.000159797	0.068714733	yes	down
ENSSSCG00000049689	CCNB1IP1	4.482317248	2.164244763	0.000437714	0.123306032	yes	up
ENSSSCG00000049831	—	5.417914772	2.437737699	0.000638959	0.145734681	yes	up
ENSSSCG00000050076	—	0.059976636	-4.059455593	0.004686285	1	yes	down
ENSSSCG00000050383	P3R3URF	0.530176621	-0.91545504	0.030903331	0.607123214	yes	down
ENSSSCG00000050531	CCNB1IP1	0.194389413	-2.362978449	0.036101439	1	yes	down
ENSSSCG00000050648	—	0.713770041	-0.486468746	0.014150727	0.456766144	yes	down
ENSSSCG00000050765	—	1.824449097	0.867460899	0.004736324	0.333646722	yes	up
ENSSSCG00000051136	CCNB1IP1	3.736644407	1.901743278	0.036386479	1	yes	up
ENSSSCG00000051278	—	0.30218166	-1.72651199	0.021593328	0.51847537	yes	down
ENSSSCG00000051300	—	0.5207079	-0.941453801	0.039470006	0.659812673	yes	down
ENSSSCG00000051565	—	0.017670898	-5.822480841	4.88E-06	0.009054952	yes	down
ENSSSCG00000051710	—	0.46278539	-1.111584774	0.029402861	0.593710253	yes	down
ENSSSCG00000051840	CCDC153	1.741258744	0.800130598	0.004323035	0.323023561	yes	up
ENSSSCG00000051862	KRAB	4.218853966	2.07685115	0.009839367	0.413744596	yes	up
ENSSSCG00000051948	—	21.57715994	4.43143308	0.010181159	0.416773906	yes	up
ENSSSCG00000052111	—	0.658664975	-0.602383259	0.048141844	0.691288485	yes	down
ENSSSCG00000052122	MUC2	1.404586171	0.490145136	0.042287967	0.671710115	yes	up
ENSSSCG00000052303	RAB32	0.751726009	-0.411721175	0.019333902	0.504239817	yes	down

ENSSSCG00000052350	TRBV	0.535918403	-0.899914736	0.042026833	0.669023907	yes	down
ENSSSCG00000052399	RDH16	7.885781091	2.979253662	0.014666788	1	yes	up
ENSSSCG00000052416	KRAB	0.382016564	-1.3882929	0.039967986	0.663444728	yes	down
ENSSSCG00000052432	MHC1	0.610317218	-0.712368804	0.011769166	0.436740665	yes	down
ENSSSCG00000052747	—	0.128329183	-2.962078811	0.038018582	1	yes	down
ENSSSCG00000052748	—	6.317251456	2.659296999	0.04165146	1	yes	up
ENSSSCG00000052785	SGK223	0.805999465	-0.311149213	0.01170048	0.436740665	yes	down
ENSSSCG00000052972	—	0.011335651	-6.46298888	0.032943748	0.621426271	yes	down
ENSSSCG00000053039	NIPSNAP3A	1.26157717	0.335228458	0.033022824	0.621700277	yes	up
ENSSSCG00000053043	—	4.290094004	2.10100926	0.017202331	0.491886525	yes	up
ENSSSCG00000053058	CXCL4	0.263449244	-1.924403053	9.23E-05	0.065519059	yes	down
ENSSSCG00000053077	TTLL10	1.584237441	0.663788579	0.011454273	0.436740665	yes	up
ENSSSCG00000053150	CTSG	1.766914587	0.821232301	0.045833155	0.687457963	yes	up
ENSSSCG00000053199	UGT	11281956.49	23.42751394	1.00E-14	1.67E-10	yes	up
ENSSSCG00000053320	CSTA_B	0.296176789	-1.755469513	0.022777586	0.525363143	yes	down
ENSSSCG00000053482	GST	0.454538981	-1.137524071	0.004394559	0.326154387	yes	down
ENSSSCG00000053507	UGT	20.9472492	4.388688896	0.007674951	0.387495665	yes	up
ENSSSCG00000053758	CTSG	0.367100396	-1.445753425	0.014066622	0.456766144	yes	down
ENSSSCG00000053773	—	0.339741732	-1.557489652	0.007367594	0.385211341	yes	down
ENSSSCG00000053834	—	12.40555445	3.632914311	0.040017826	1	yes	up
ENSSSCG00000053885	—	0.57221042	-0.805382326	0.016410569	0.484426375	yes	down
ENSSSCG00000053975	—	0.405069193	-1.303759728	0.027389332	0.572788782	yes	down
ENSSSCG00000054006	PARK2	2.813972945	1.492608458	0.046028491	0.687457963	yes	up
ENSSSCG00000054082	TRBV	0.347630206	-1.524374651	0.004166821	0.323023561	yes	down
ENSSSCG00000054264	BRI3BP	1.21608291	0.282241592	0.032884193	0.621426271	yes	up
ENSSSCG00000054412	CTSG	6.032431994	2.592739746	0.04981254	1	yes	up
ENSSSCG00000054480	SHISA4	0.636027885	-0.652838076	0.001328256	0.198589146	yes	down
ENSSSCG00000054636	—	2.356154708	1.236434272	0.031863447	0.612165162	yes	up
ENSSSCG00000054658	—	6.070586177	2.60183583	0.006001874	1	yes	up
ENSSSCG00000054869	—	2.402466912	1.264516562	0.00137499	0.198589146	yes	up
ENSSSCG00000054936	RBP5	0.655226464	-0.609934467	0.004333031	0.323023561	yes	down
ENSSSCG00000054957	—	2.031307496	1.022408649	0.012708444	0.447694574	yes	up
ENSSSCG00000054999	ZBTB21	1.424205755	0.510157587	0.014176194	0.456766144	yes	up
ENSSSCG00000055012	—	0.570223077	-0.810401668	0.018430361	0.499624348	yes	down
ENSSSCG00000055147	ITGAX	0.635719381	-0.653538023	0.038392877	0.650310844	yes	down
ENSSSCG00000055209	—	14.51856032	3.859826496	0.028044091	1	yes	up
ENSSSCG00000055221	—	1.369787655	0.453952264	0.04701277	0.691288485	yes	up
ENSSSCG00000055296	—	3.485654281	1.801429485	0.006741461	0.368533767	yes	up
ENSSSCG00000055506	KRAB	5.484721081	2.455418256	0.04411637	1	yes	up
ENSSSCG00000055646	NEK1_4_5	6.978158681	2.802846404	0.048911261	1	yes	up
ENSSSCG00000055702	CENPO	1.325928015	0.407002453	0.02943981	0.593738399	yes	up

ENSSSCG00000055724	—	0.612651049	-0.70686251	0.018501137	0.50073013	yes	down
ENSSSCG00000055825	RP-L26e	2.070884483	1.05024708	0.024377978	0.545694169	yes	up
ENSSSCG00000055872	—	0.440645718	-1.182308911	0.031055475	0.607254547	yes	down
ENSSSCG00000056251	CCL22	0.465785505	-1.102262351	0.03693544	0.643154238	yes	down
ENSSSCG00000056525	IGH	0.277027845	-1.8518971	9.68E-05	0.065519059	yes	down
ENSSSCG00000056544	MRGPRX	4.164739317	2.058226196	0.029926655	0.598270325	yes	up
ENSSSCG00000056642	—	0.75155225	-0.412054687	0.026114583	0.563539972	yes	down
ENSSSCG00000056653	MRGPRX	2.224757246	1.153647926	0.048134152	0.691288485	yes	up
ENSSSCG00000056729	C5AR1	0.602553294	-0.730839246	0.008980235	0.394634056	yes	down
ENSSSCG00000056929	—	5.241039488	2.389852979	0.038146956	1	yes	up
ENSSSCG00000056939	KRAB	0.692773764	-0.5295438	0.032901325	0.621426271	yes	down
ENSSSCG00000057001	CTSG	3.511892414	1.812248649	0.043243838	0.678952552	yes	up
ENSSSCG00000057040	—	0.761258281	-0.393542078	0.018705142	0.504122241	yes	down
ENSSSCG00000057146	—	1.230531419	0.299281494	0.047759587	0.691288485	yes	up
ENSSSCG00000057278	CT55	1.566526355	0.647569042	0.011660668	0.436740665	yes	up
ENSSSCG00000057366	—	0.115696139	-3.111587378	0.047856502	1	yes	down
ENSSSCG00000057393	HSD17B13	2.311483172	1.20881886	0.042259136	0.671710115	yes	up
ENSSSCG00000057430	JMJD7	1.778885443	0.830973607	0.007958566	0.387495665	yes	up
ENSSSCG00000057482	—	2.229495093	1.156717024	0.001569009	0.209336848	yes	up
ENSSSCG00000057599	KRAB	7.468321011	2.90078394	0.036943554	1	yes	up
ENSSSCG00000057701	CES1	0.29070996	-1.782347593	0.000662954	0.145734681	yes	down
ENSSSCG00000057703	—	0.255180142	-1.970412032	0.019788946	0.504766715	yes	down
ENSSSCG00000057730	—	7.150358802	2.838015638	0.017262699	1	yes	up
ENSSSCG00000057900	—	1.984377813	0.988686732	0.041877095	0.667913669	yes	up
ENSSSCG00000057970	PLBD1	0.616397745	-0.69806651	0.044007401	0.679567425	yes	down
ENSSSCG00000057986	—	0.24175794	-2.048364825	0.034502883	1	yes	down
ENSSSCG00000058009	SIGLEC5	0.500373309	-0.99892326	0.00716349	0.381350169	yes	down
ENSSSCG00000058031	—	0.598207766	-0.741281454	0.016819955	0.485997014	yes	down
ENSSSCG00000058123	PARK2	0.129636922	-2.947451424	0.027182956	1	yes	down
ENSSSCG00000058218	—	2.835958029	1.503836181	0.001518042	0.208200082	yes	up
ENSSSCG00000058226	UGT	2.767895688	1.468789574	0.032177809	0.614363628	yes	up
ENSSSCG00000058261	TEX12	0.158320671	-2.659078459	0.013567459	0.449667171	yes	down
ENSSSCG00000058286	KRAB	1.265483958	0.339689219	0.011355661	0.436147095	yes	up
ENSSSCG00000058313	—	1.678450958	0.747130384	0.033684015	0.626076307	yes	up
ENSSSCG00000058337	—	0.213138	-2.230140263	0.046037766	0.687457963	yes	down
ENSSSCG00000058473	—	5.482637322	2.454870042	0.01945006	1	yes	up
ENSSSCG00000058502	CYP2J	0.672997003	-0.571328015	0.015213132	0.467600207	yes	down
ENSSSCG00000058553	—	2.256751855	1.174247793	0.035844211	0.633787122	yes	up
ENSSSCG00000058587	—	0.053673631	-4.219642691	0.006847783	1	yes	down
ENSSSCG00000058669	GPR15	0.557149349	-0.843863989	0.018754181	0.504122241	yes	down
ENSSSCG00000058723	CD1D	0.629029125	-0.668801277	0.031731314	0.612165162	yes	down

ENSSSCG00000058826	—	1.63537153	0.70961843	0.00745324	0.385459407	yes	up
ENSSSCG00000058919	MAFB	0.603936461	-0.727531321	0.010912749	0.430808481	yes	down
ENSSSCG00000058971	FAM219A	0.779058645	-0.360196161	0.028636534	0.58539213	yes	down
ENSSSCG00000059002	—	6.17520398	2.626486793	0.031186031	1	yes	up
ENSSSCG00000059227	FOXQ	3.753526121	1.908246523	0.024173408	0.543299782	yes	up
ENSSSCG00000059249	CD200R	0.139856229	-2.837983583	0.027287392	1	yes	down
ENSSSCG00000059250	—	13.92134237	3.799226425	0.006011631	0.366967563	yes	up
ENSSSCG00000059519	—	1.300636014	0.379217277	0.047961132	0.691288485	yes	up
ENSSSCG00000059535	CCNB1IP1	3.664894692	1.873771744	0.040416124	1	yes	up
ENSSSCG00000059684	MRGPRX	9.325124649	3.22112301	0.007123739	1	yes	up
ENSSSCG00000059697	—	4.222140772	2.077974681	0.038075598	0.648139058	yes	up
ENSSSCG00000059775	LILR	0.519373641	-0.945155298	0.008190561	0.387495665	yes	down
ENSSSCG00000059854	—	2.642531042	1.401920419	0.045598165	0.687082715	yes	up
ENSSSCG00000059937	HOX_4	1.592817715	0.671581171	0.01808561	0.496729606	yes	up
ENSSSCG00000059948	PRG3	20.40712377	4.351000955	0.006895017	1	yes	up
ENSSSCG00000059961	—	1.93655701	0.953493973	0.012320354	0.442351644	yes	up
ENSSSCG00000059997	MALL	1.205430852	0.269548895	0.018116876	0.496771298	yes	up
ENSSSCG00000060016	—	3.860819712	1.948907187	0.002873702	0.275792815	yes	up
ENSSSCG00000060070	CD33	0.584518688	-0.774678944	0.011724185	0.436740665	yes	down
ENSSSCG00000060097	NEK1_4_5	0.661308985	-0.596603592	0.004506036	0.326473146	yes	down
ENSSSCG00000060176	CTSG	0.331974503	-1.590855653	0.022716022	0.524686071	yes	down
ENSSSCG00000060422	—	2.01610384	1.011569947	0.018363446	0.499431886	yes	up
ENSSSCG00000060610	—	3.95786421	1.984722116	0.036340605	1	yes	up
ENSSSCG00000060736	FOXD	1.295134502	0.373101932	0.017783284	0.492991747	yes	up
ENSSSCG00000060872	—	0.300752264	-1.733352497	0.020657994	0.511821734	yes	down
ENSSSCG00000061010	—	12.21717742	3.610839108	0.02500757	1	yes	up
ENSSSCG00000061341	—	0.476209882	-1.070330537	0.048829618	0.695145602	yes	down
ENSSSCG00000061491	—	2.085630114	1.060483319	0.001174806	0.192334242	yes	up
ENSSSCG00000061763	—	0.539710935	-0.889741176	0.021394024	0.517017084	yes	down
ENSSSCG00000061823	KRAB	4.874052315	2.285121736	0.027217155	1	yes	up
ENSSSCG00000061832	KRAB	1.762569223	0.817679919	0.043725325	0.678952552	yes	up
ENSSSCG00000062029	FUT4	1.247638313	0.319199761	0.028654683	0.58539213	yes	up
ENSSSCG00000062134	TRPV5	0.615943744	-0.699129504	0.012806128	0.447694574	yes	down
ENSSSCG00000062169	IL26	0.260334619	-1.941560923	0.036423185	1	yes	down
ENSSSCG00000062212	—	4.440412368	2.150693662	0.017449404	1	yes	up
ENSSSCG00000062238	—	0.667481456	-0.583200338	0.027419014	0.572788782	yes	down
ENSSSCG00000062259	ICAM2	0.722011117	-0.469907043	0.008119491	0.387495665	yes	down
ENSSSCG00000062411	—	3.804587532	1.927740055	0.040258945	1	yes	up
ENSSSCG00000062424	PDF	1.497032856	0.582105885	0.01011348	0.416773906	yes	up
ENSSSCG00000062467	—	0.251056497	-1.993916035	0.001482246	0.208200082	yes	down
ENSSSCG00000062585	RNASE6	0.413890717	-1.272678204	0.019325087	0.504239817	yes	down

ENSSSCG00000062590	GPR31	0.561545411	-0.8325254	0.028134608	0.580741434	yes	down
ENSSSCG00000062593	—	0.076930439	-3.700301644	0.017523242	1	yes	down
ENSSSCG00000062758	—	2.417673381	1.273619355	0.008056555	0.387495665	yes	up
ENSSSCG00000062784	—	6.150729407	2.620757508	0.044422422	1	yes	up
ENSSSCG00000063043	INAFM2	1.931278152	0.949555963	0.040741078	0.667123021	yes	up
ENSSSCG00000063100	—	0.024221216	-5.3675849	0.041164213	1	yes	down
ENSSSCG00000063120	C4orf48	0.501260476	-0.996367612	0.00531532	0.348080505	yes	down
ENSSSCG00000063177	—	1.590996409	0.66993058	0.045146699	0.682884715	yes	up
ENSSSCG00000063361	—	4.0712922	2.025486769	0.046787358	1	yes	up
ENSSSCG00000063387	AKR1C8	0.525770978	-0.927493585	0.006505037	0.366967563	yes	down
ENSSSCG00000063391	—	5.177741401	2.372322913	0.020227762	1	yes	up
ENSSSCG00000063395	—	0.414513778	-1.270508039	0.012147547	0.441694027	yes	down
ENSSSCG00000063413	—	6.342538659	2.665060408	0.013378441	1	yes	up
ENSSSCG00000063511	IGH	0.26898405	-1.89440747	4.40E-05	0.049022187	yes	down
